# Diverse reactivity of a tricoordinate organoboron L_2_PhB: (L = oxazol-2-ylidene) towards alkali metal, group 9 metal, and coinage metal precursors†
†Electronic supplementary information (ESI) available: Experimental and calculation details, and crystallographic information for **2**, **3**, **4**, **6**, **8**. CCDC 1038665, 1038666, 1038667, 1011534, and 1011533. For ESI and crystallographic data in CIF or other electronic format see DOI: 10.1039/c5sc00404g



**DOI:** 10.1039/c5sc00404g

**Published:** 2015-02-23

**Authors:** Lingbing Kong, Rakesh Ganguly, Yongxin Li, Rei Kinjo

**Affiliations:** a Division of Chemistry and Biological Chemistry , School of Physical and Mathematical Sciences , Nanyang Technological University , Singapore 637371 , Singapore . Email: rkinjo@ntu.edu.sg; b NTU-CBC Crystallography Facility , Nanyang Technological University , Singapore 637371 , Singapore

## Abstract

The reactivity of a tricoordinate organoboron L_2_PhB: (L = oxazol-2-ylidene) **1** towards metal precursors and its coordination chemistry were comprehensively studied. While the boron center in **1** is reluctant to coordinate to the alkali metals in their trifluoromethanesulfonate salts (MOTf) (M = Li, Na, K), the unprecedented compound **2** containing two L_2_PhB: units linked by a cyclic Li(OTf)_2_Li spacer was obtained from the reaction of **1** with LiOTf. Treatment of **1** with group 9 metal complexes [MCl(COD)]_2_ (M = Rh, Ir) afforded the first zwitterionic rhodium(i)–boronium complex **3** and the iridium(iii)–borane complex **4**, respectively. The reaction pathway may involve C–H activation followed by proton migration from the metals to the boron center, demonstrating the first example of the deprotonation of metal hydrides by a basic boron. In the reactions with coinage metals, **1** could act as a two-electron reducing agent towards the metal chlorides MCl (M = Cu, Ag, Au). Meanwhile, the reaction of **1** with gold chloride supported by a N-heterocyclic carbene (NHC) produced a heteroleptic cationic gold complex [(L_2_PhB)Au(NHC)]Cl (**6**) featuring both carbene and L_2_PhB: ligands on the gold atom. In contrast, an isolable gold chloride complex (L_2_PhB)AuCl (**8**) was obtained by direct complexation between **1** and triphenylphosphine-gold chloride *via* ligand exchange. X-ray diffraction analysis and computational studies revealed the nature of the B:→Au bonding interaction in complexes **6** and **8**. Natural Population Analysis (NPA) and Natural Bond Orbital (NBO) analysis support the strong σ-donating property of the L_2_PhB: ligand. Moreover, preliminary studies showed that complex **8** can serve as an efficient precatalyst for the addition of X–H (X = N, O, C) to alkynes under ambient conditions, demonstrating the first application of a metal complex featuring a neutral boron-based ligand in catalysis.

## Abstract 2

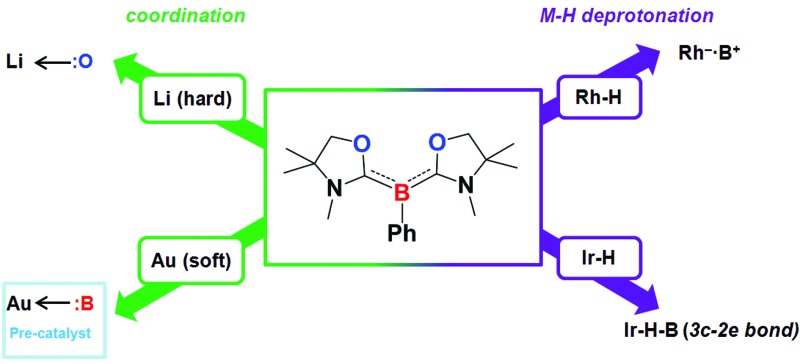
The reactivity of a tricoordinate organoboron L_2_PhB: (L = oxazol-2-ylidene) **1** towards metal precursors and its coordination chemistry were comprehensively studied.

## Introduction

**Fig. 1:**
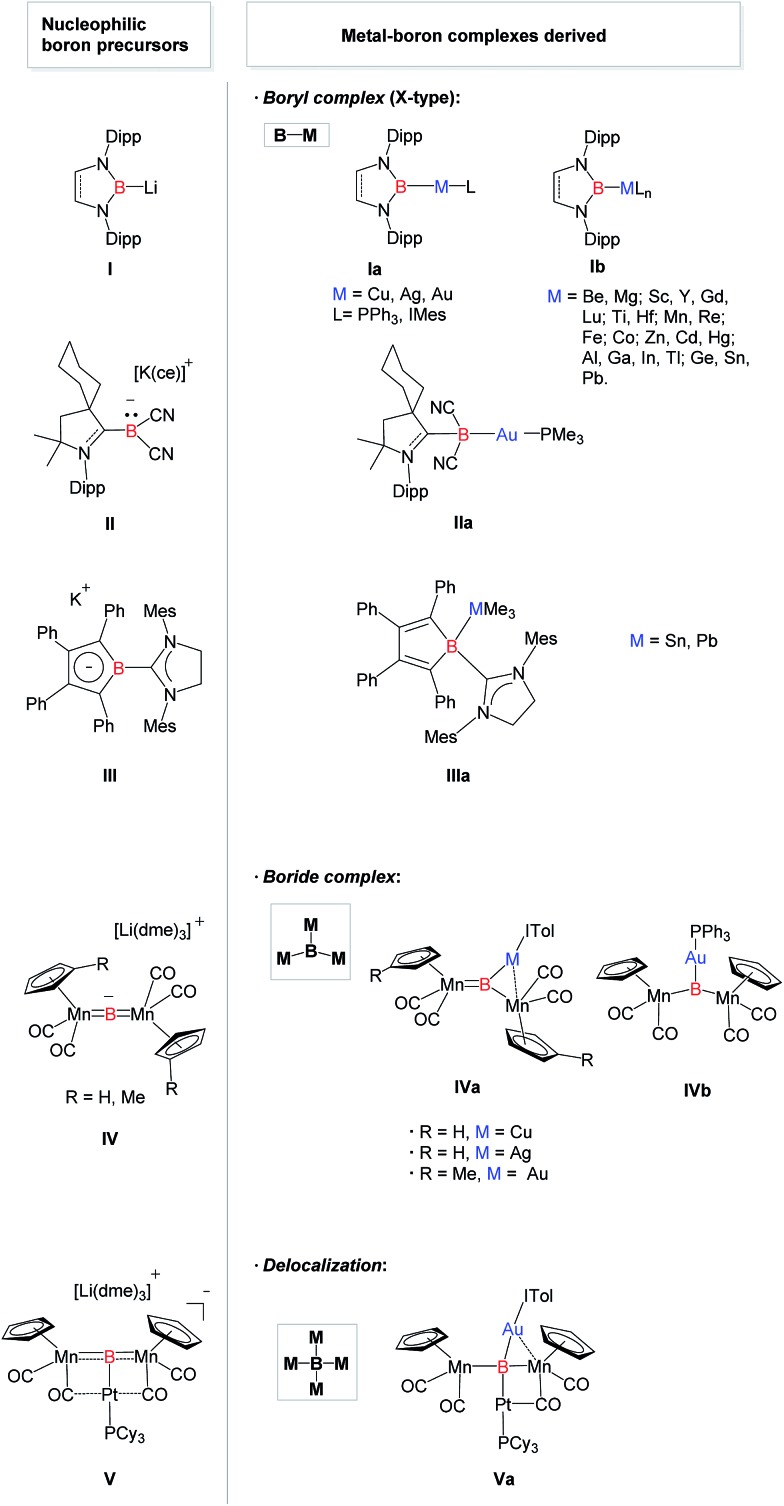
Structurally characterized metal complexes **Ia–Va** featuring a boron-centered ligand derived from the corresponding nucleophilic boron species **I–V**. [Cy = cyclohexyl, Dipp = 2,6-diisopropylphenyl, IMes = 1,3-bis(mesityl)imidazol-2-ylidene, ITol = 1,3-bis(tolyl)imidazol-2-ylidene, Mes = 2,4,6-trimethylphenyl, dme = 1,2-dimethoxyethane, ce = dibenzo-18-crown-6].

Transition metal complexes featuring boron-based ligands have attracted significant attention in boron chemistry because of the fundamental importance of understanding basic metal–boron bonding properties and their potential applications as catalysts in organic synthesis.^1^ Depending on the coordination number of boron and the metal–boron bonding mode, they are classified as boron cluster, borane, boryl, borylene, and boride complexes. In addition to several conventional strategies for the preparation of complexes featuring unique metal–boron bonding interactions, a new general methodology, namely direct transmetalation between isolable nucleophilic boron species and metal precursors, has been developed in recent years (Fig. 1). In 2007, Nozaki, Yamashita *et al.* reported the first nucleophilic attack of boryl lithium **I** ^2^ on coinage metal chlorides supported by triphenylphosphine (Ph_3_P) or N-heterocyclic carbene (NHC) ligands which afforded the corresponding boryl complexes **Ia** possessing two-center two-electron M–B (M = Cu, Ag, Au) bonds.^3^ Since then, a large number of boryl complexes **Ib** have been synthesized by a similar approach, in which the metals varied extensively from rare earth metals and transition metals to main group metals.^4^ Similarly, Bertrand *et al.* illustrated that CAAC-stabilized boryl anion **II** can also be used as a nucleophile to synthesize the gold–boryl complex **IIa**.^5^ Braunschweig *et al.* revealed the radical reactivity of the boryl anion **III** towards heavier tetrel halides to produce **IIIa** possessing rare B–Sn and B–Pb bonds.^6^ They also showed that salt elimination between anionic dimetalloborylene **IV** and NHC-coinage metal chlorides afforded the trimetallic boride complexes **IVa** that contain delocalized Mn–B–M (M = Cu, Ag, Au) bonding,^7,8^ whereas **IVb** was obtained when Ph_3_P–gold chloride was used.^8^ In 2012, the same group isolated a novel planar tetra-metalated boron complex **Va** from the reaction of **V** with (NHC)AuCl, and proposed unique σ and π metal–boron bonding interactions in **Va**.^9^

**Fig. 2:**
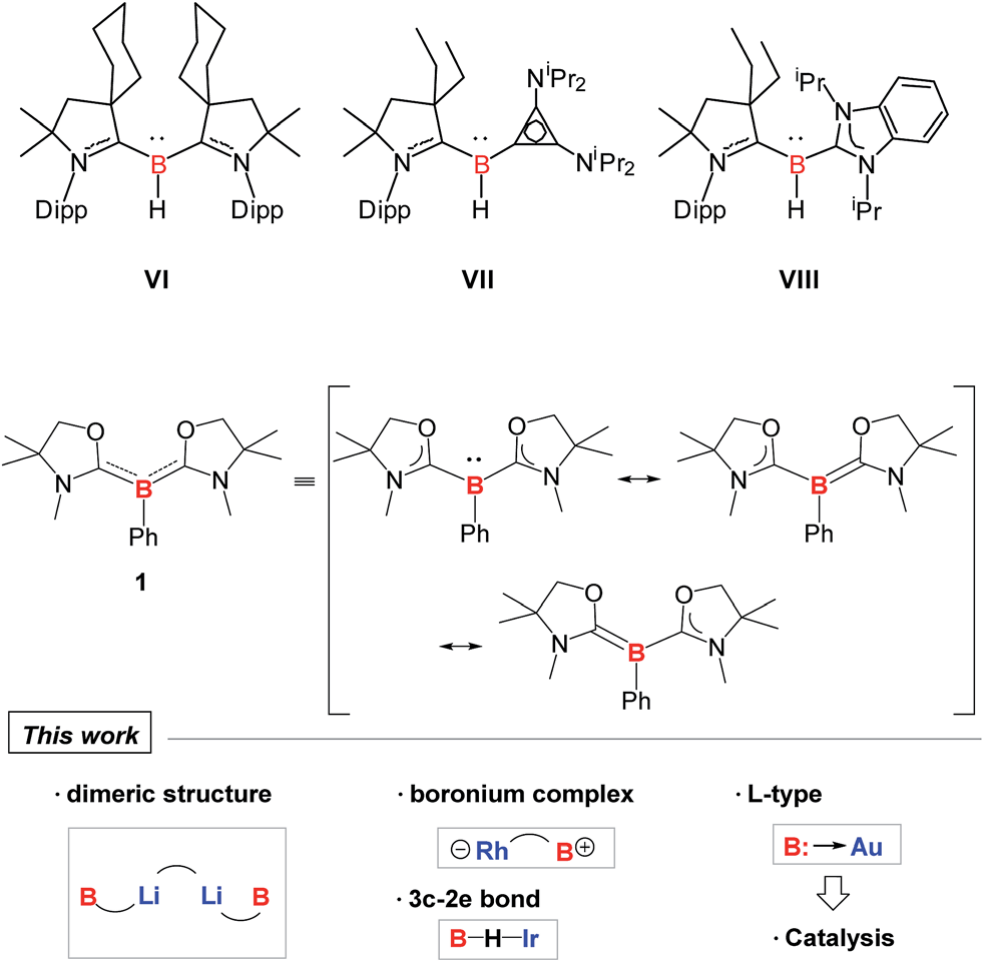
Isolated examples of tricoordinate organoborons **VI–VIII** and **1** that are isoelectronic with amines.

Although a variety of M–B bonding modes have been achieved through reactions between metal precursors and nucleophilic boron species as mentioned above, such an approach is limited to the construction of M–B single bonds mainly in their covalent fashion, and cannot be applied in the formation of the B:→M bonding mode despite the significance from the fundamental and application points of view.^1^ In addition, conventional synthetic methodology for the complexes bearing B:→M bonds cannot be applicable to all metals due to the lack of the reducing strength of some metals towards boron–halide bonds, such as for coinage metals. Undoubtedly, it will be straightforward if direct coupling between metal precursors and ligands possessing a lone pair on the boron is available. In 2011, Bertrand and co-workers reported the first isolation of tricoordinate nucleophilic organoboron **VI** that is isoelectronic with amines (Fig. 2).^10,11^ They also developed an efficient synthetic route to relevant derivatives **VII** and **VIII**.^12^ Despite the development of unique compounds **VI–VIII**, studies dealing with them are limited to their protonation reaction with Brønsted acids that afforded boronium species, and oxidation to generate radical cations. Recently, we reported the synthesis of L_2_PhB: (**1**) (L = oxazol-2-ylidene) and elucidated that **1** can readily react with (thf)Cr(CO)_5_ to afford the complex (L_2_PhB)Cr(CO)_5_, underlining its nucleophilic character (Fig. 2).^13^ This result prompted us to investigate the further reactivity of **1** towards metal precursors. Herein, we report the reactivity of L_2_PhB: (**1**) towards alkali and late transition metal derivatives, and discuss the outcome of these reactions based on spectroscopic analysis, single-crystal X-ray diffraction and computational studies.

## Results

### Reactivity of **1** towards alkali metal salts

**Scheme 1:**
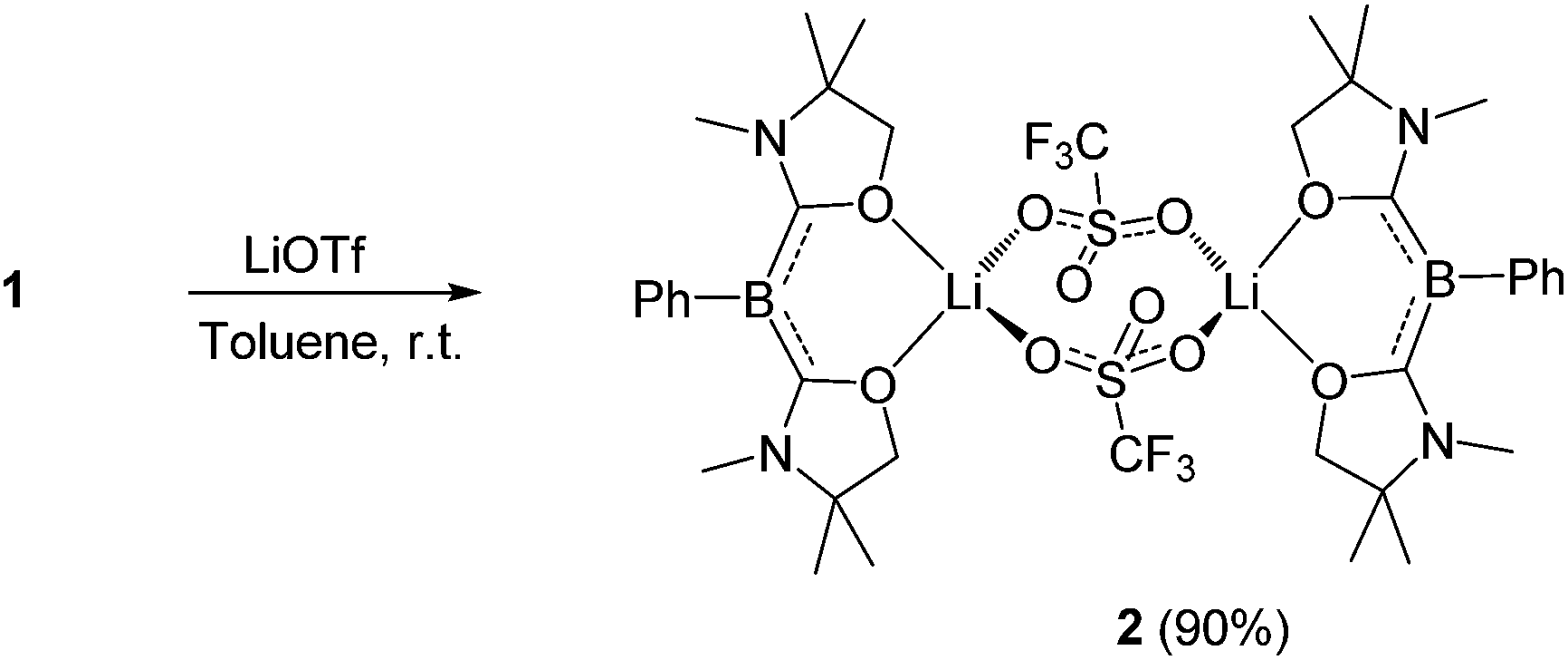
Reaction of **1** with LiOTf.

First, we attempted the reaction between **1** and lithium trifluoromethanesulfonate (LiOTf). Treatment of **1** with an equivalent of LiOTf in toluene for 48 h at ambient temperature afforded a yellow precipitate. After removal of the solvent under vacuum, the residue was washed with *n*-hexane to afford a yellow powder of **2** in 90% yield (Scheme 1). The ^11^B NMR spectrum of **2** in C_6_D_6_ displays a singlet at 1.74 ppm, which is shifted only 0.07 ppm downfield compared with that of **1**, implying that the boron center is inert towards lithium salt.

**Fig. 3:**
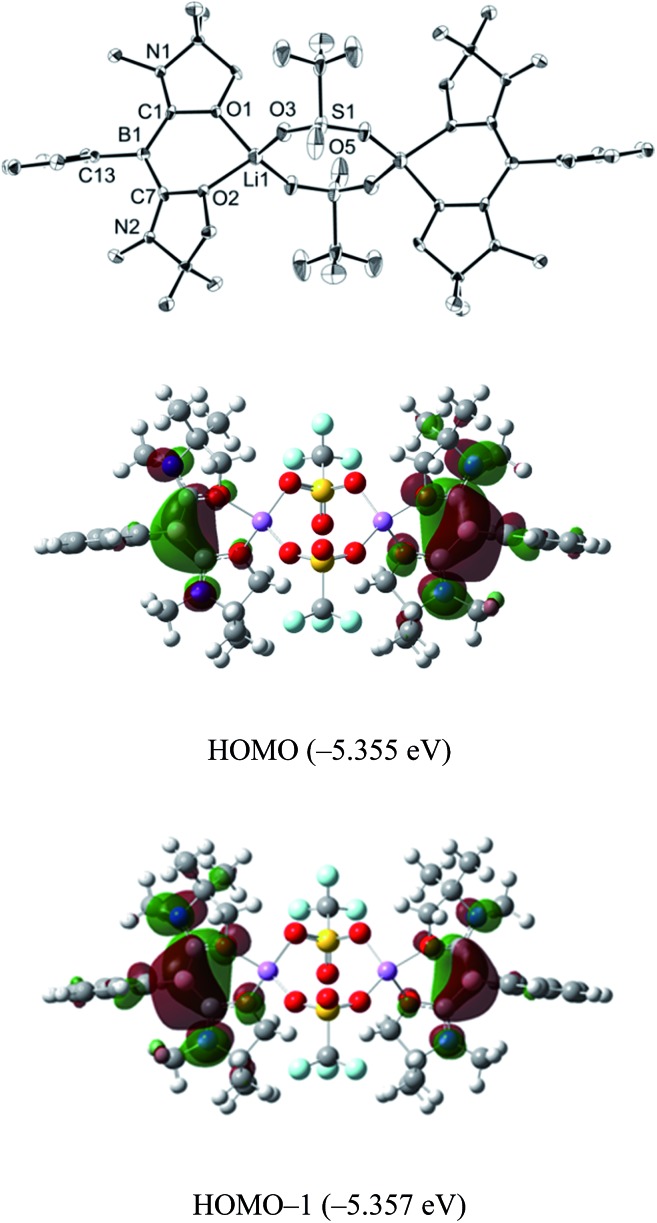
Solid-state structure of **2** (top) (thermal ellipsoids are set at the 50% probability level. Hydrogen atoms are omitted for clarity). Plots of the HOMO (middle) and the HOMO–1 (bottom) of **2** (calculated at the M05-2X/6-311G(d,p) level of theory).

Single crystals were obtained from a saturated toluene solution of **2** and the solid state structure was confirmed by an X-ray diffraction study (Fig. 3).^14^ Compound **2** contains two L_2_PhB: units and two LiOTf molecules. Two oxygen atoms from each oxazol-2-ylidene coordinate to a lithium atom forming a BC_2_O_2_Li six-membered ring. The two lithium atoms are linked by two OTf units in which two oxygen atoms from each OTf unit coordinate to different lithium atoms to form a LiO_2_S_2_O_2_Li eight-membered ring, with the CF_3_ groups on the S atoms in a *trans* orientation. The geometry around the boron centers remains trigonal planar (sum of the angles = 360.0°) and structural parameters in each of the L_2_PhB: moieties are nearly identical to those in compound **1**. To gain insight into the electronic properties, we performed quantum chemical calculations for **2**. Electron delocalization over the two C–B–C units was confirmed in the HOMO (–5.355 eV) and the HOMO–1 (–5.357 eV) that are nearly degenerate (Fig. 3). Interestingly, these MOs are lower in energy than the HOMO (–4.80 eV) of compound **1**, presumably caused by the coordination of oxygen atoms to the Lewis acidic Li atoms, which reduces the electron density in the L_2_PhB: units. Thus, the nucleophilicity of **1** might be tunable depending on the property of the units coordinating to these O-atoms. The formation of **2** rather than B:→Li coordination can be rationalized by the hard acidic and oxophilic properties of lithium in contrast to the nature of the boron center as a soft base. Although compound **2** is thermally stable, it decomposes upon exposure to air. Attempts to react **1** with NaOTf and KOTf, containing metals slightly softer than Li, afforded neither B:→M (M = Na, K) coordination nor the corresponding analogues to **2**.

### Reactivity of **1** towards group 9 metals

**Scheme 2:**
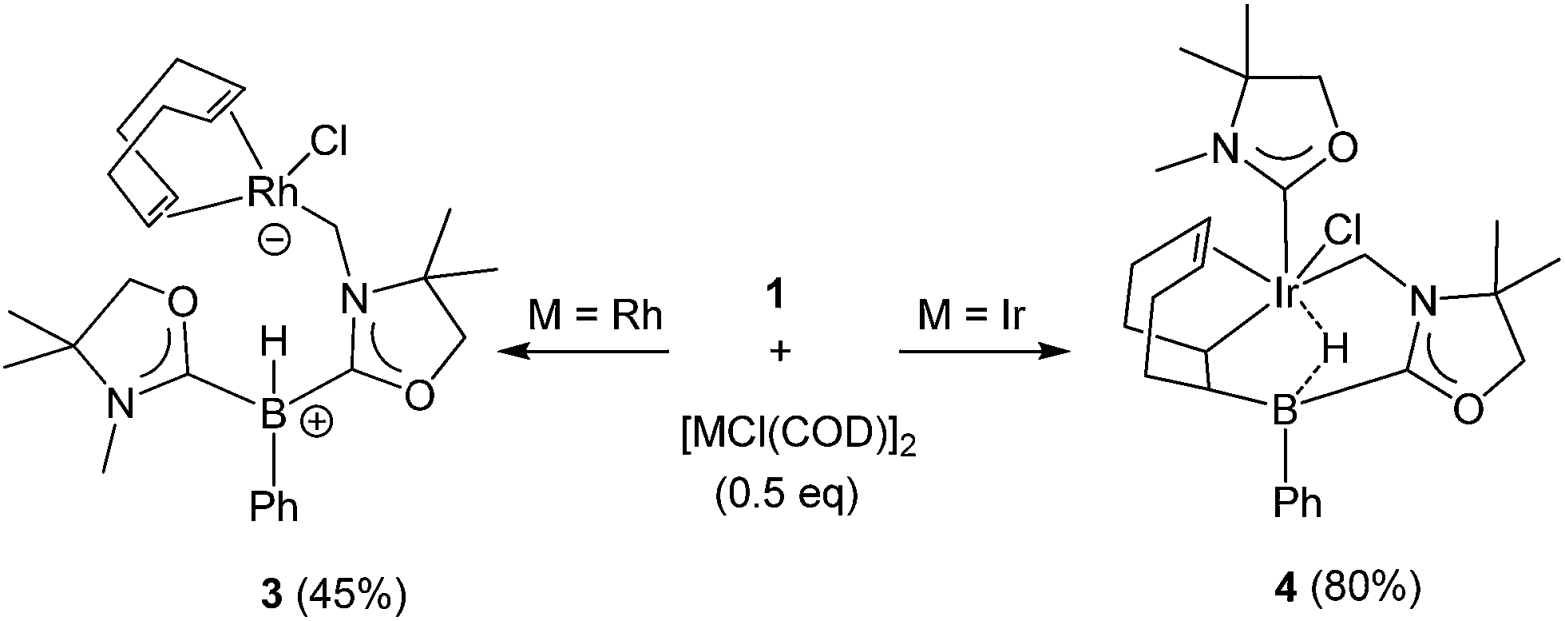
Reactions of **1** with [MCl(COD)_2_] (M = Rh, Ir).

**Fig. 4:**
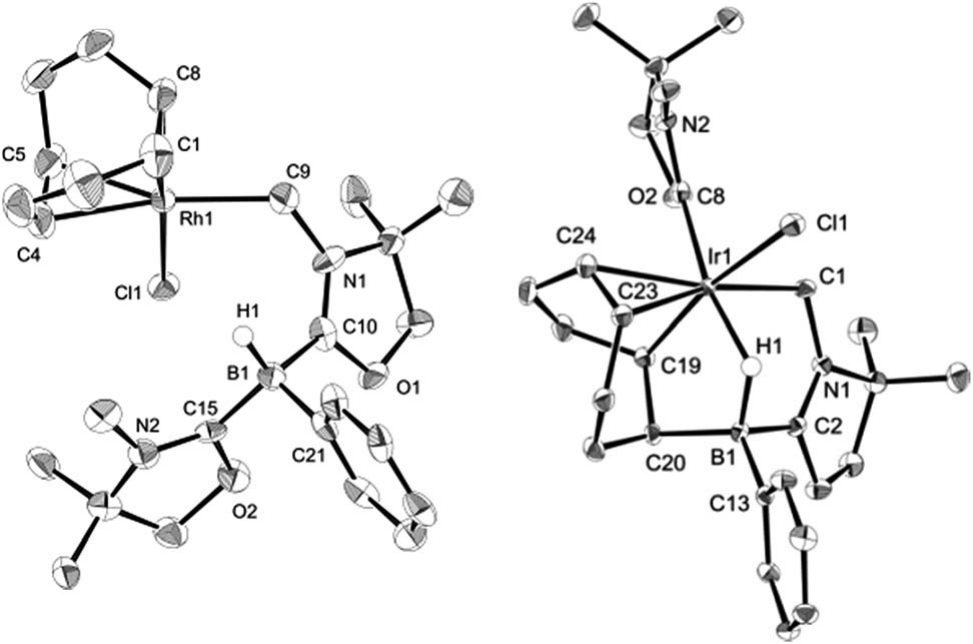
Solid-state structures of **3** (left) and **4** (right) (hydrogen atoms except for those on the B atom are omitted for clarity). Thermal ellipsoids are set at the 50% probability level.

Reactivity of **1** towards late transition metals was investigated employing rhodium and iridium complexes. To a THF solution of **1**, 0.5 equivalent of [RhCl(COD)]_2_ was added at ambient temperature. After removal of the solvent *in vacuo*, the residue was washed with cold *n*-hexane to afford a yellow solid of **3** in 45% yield (Scheme 2). The ^11^B NMR spectrum of **3** displays a doublet at –21.0 ppm which is due to boron–hydrogen coupling (^1^ *J*_BH_ = 34.0 Hz). In the ^13^C NMR spectrum, a new peak for *C*H_2_ appeared at 39.7 ppm as a doublet (^1^ *J*_RhC_ = 31.8 Hz), indicating the formation of a rhodium–carbon bond. The solid structure of **3** was determined by a single crystal X-ray diffraction study, which revealed the unique zwitterionic property of **3** involving a boronium cation and an anionic rhodium(i) center (Fig. 4, left).^14^ Note that only a few anionic Rh(i) complexes have been described and structurally characterized thus far, and to the best of our knowledge, no rhodium complexes including a boronium fragment have been reported before.^15^ The anionic Rh center coordinated by COD is covalently bonded to a chlorine atom and the *C*H_2_ carbon [Rh–Cl: 2.408(3) Å. Rh–*C*H_2_: 2.100(10) Å]. The distance between the Rh and the B atoms is greater than 3.6791(5) Å, indicating no interaction between them. The geometric parameters around the boron center are almost identical to those previously reported in the boronium [L_2_PhBH]OTf.^13^

**Fig. 5:**
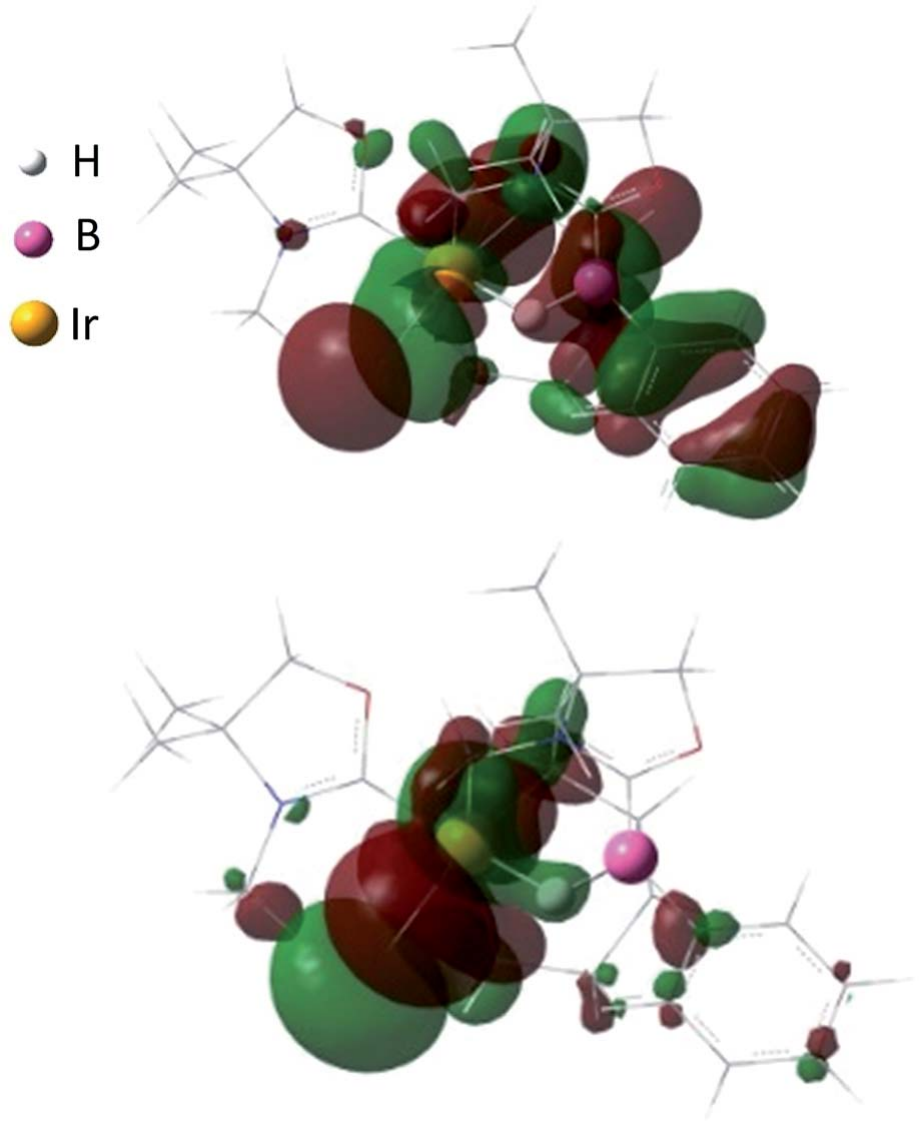
Plots of the HOMO (–7.043 eV) (top) and HOMO–1 (–7.161 eV) (bottom) of **4** (calculated at the M05-2X/6-311G(d,p) level of theory with the LANL2TZ(f) pseudopotential applied for the iridium atom).

Reaction of **1** with 0.5 equivalent of [IrCl(COD)]_2_ led to the formation of a new complex **4** in 80% yield (Scheme 2). In the ^1^H NMR spectrum of **4**, a characteristic broad peak was observed at –7.54 ppm, indicating a strong interaction of the H atom with the iridium center. The ^11^B NMR spectrum of **4** showed a broad peak at –22.7 ppm, corresponding to the tetracoordinate boron. Single crystals of **4** were obtained from a saturated toluene solution at room temperature. In the solid structure of **4**, one of the oxazol-2-ylidenes coordinates to the Ir center in an η^1^-fashion (Fig. 4, right).^14^ In contrast to the bonding situation in **3** where two C=C moieties in COD coordinate to the Rh center, both the Ir and the B atoms in **4** form covalent bonds with carbons in the COD, and only one C=C part interacts with the Ir atom in an η^2^-fashion. Taking NMR data and the structural parameters of **4**, the presence of the B–H–Ir bonding interaction can be postulated. Indeed, the experimental geometry is well reproduced by a DFT calculation on **4**, where the Ir–H and B–H distances are 1.78 Å and 1.31 Å; these are 0.06 Å and 0.16 Å longer than the sum of the covalent radii of Ir–H and B–H respectively.^16^ Thus, it can be illustrated as a 3c-2e Ir–H–B bond.^17^ The B–H and Ir–H bonding interactions are involved in the HOMO and HOMO–1, respectively (Fig. 5). Natural Bond Orbital (NBO) analysis gave Wiberg bond index (WBI) values for the B–H bond (0.57) and Ir–H bond (0.30). The solid-state IR spectrum of **4** displays an Ir–H–B bending vibration at 1618 cm^–1^, which is in line with the computational result (mode 167: 1589 cm^–1^, see the ESI†).

**Scheme 3:**
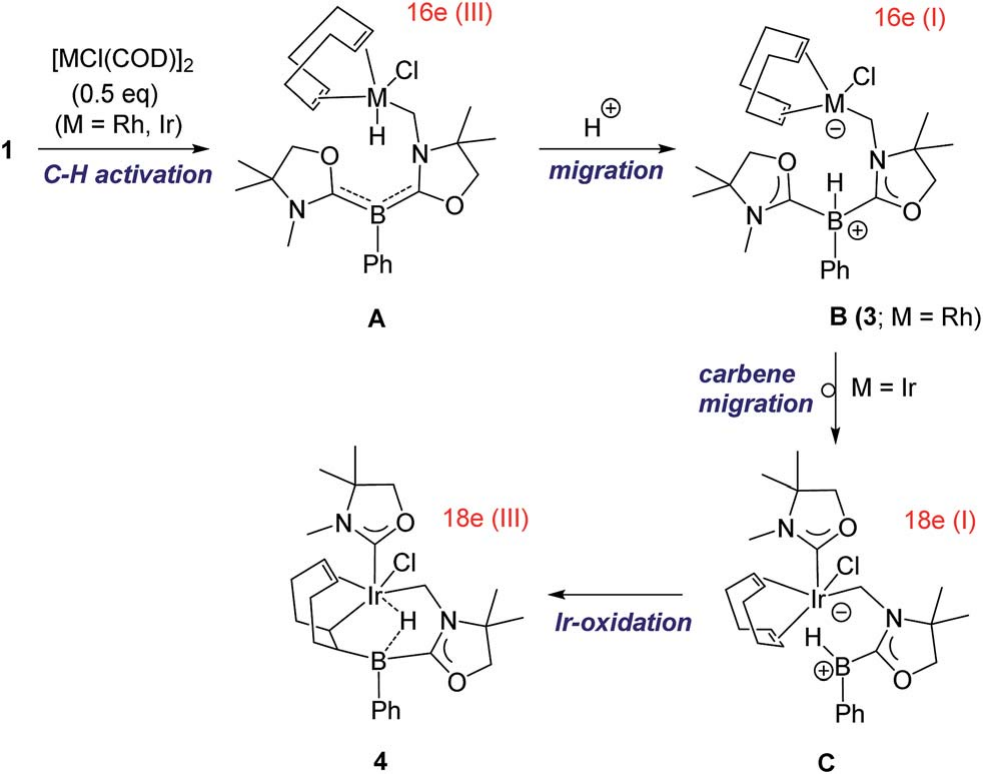
Proposed reaction pathway for the formation of **3** and **4** from reactions of **1** with [MCl(COD)]_2_ (M = Rh, Ir).

A proposed reaction pathway for the formation of **3** and **4** is drawn in Scheme 3. As no metal complexes containing B:→M bonding were observed in these reactions even when reactions were conducted at low temperature, the reactions may be initiated by C–H activation at one of the methyl groups on nitrogen atoms to generate 16 e metal hydride **A**. Subsequent proton migration from the metal center to the boron atom would lead to zwitterionic species **B** in the +I oxidation state. Note that it can be regarded as the first example of deprotonation from a metal hydride by the basic boron center. For the case of Ir, further migration of oxazol-2-ylidene from the boron to the iridium center would afford intermediate **C** which involves an anionic 18 e Ir(i) atom and a borenium cation. Addition of the Ir and the B atoms across one of the C=C bonds in COD results in the formation of the iridium borane complex **4** bearing a 3c-2e B–H–Ir bond.

### Reactivity of **1** towards coinage metal compounds

**Scheme 4:**
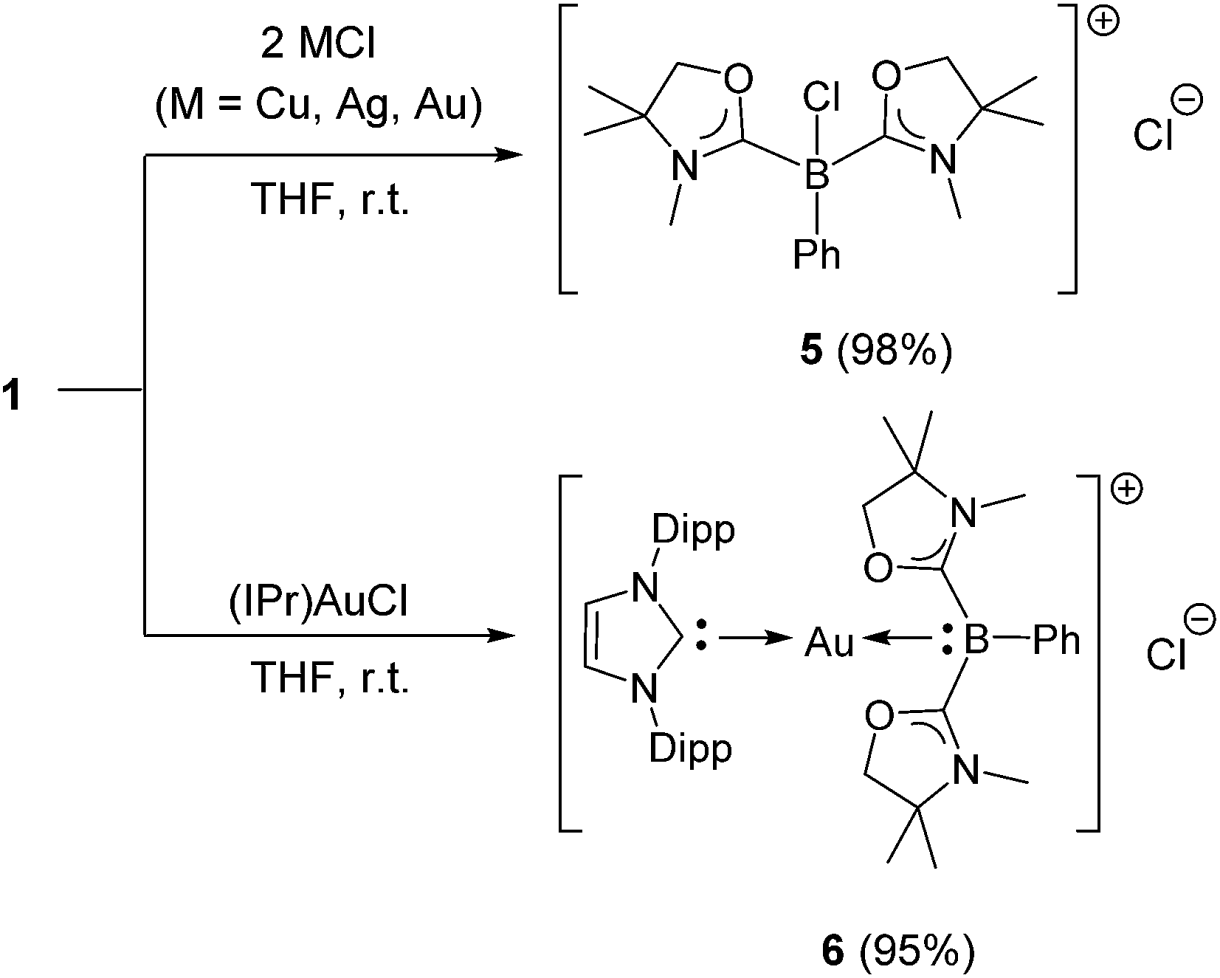
Reactions of **1** with MCl (M = Cu, Ag, Au) and (IPr)AuCl (Dipp = 2,6-diisopropylphenyl).

**Fig. 6:**
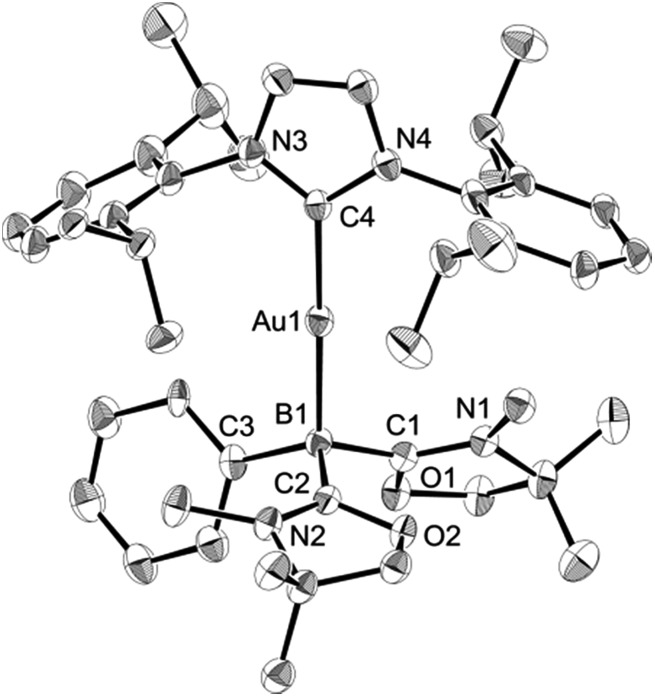
Solid-state structure of **6** (hydrogen and chlorine atoms and solvent molecules are omitted for clarity). Thermal ellipsoids are set at the 50% probability level.

**Fig. 7:**
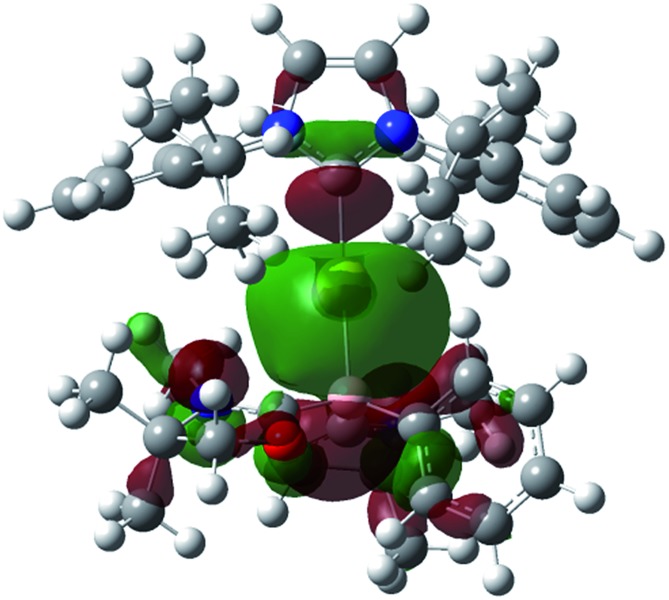
Plot of the HOMO (–9.138 eV) of **6** (calculated at the M05-2X/6-311G(d,p) level of theory, with the LANL2TZ(f) pseudopotential applied for the gold atom).

Numerous coinage metals featuring boron-based ligand have been isolated and structurally characterized.^18^ Although various types of bonding interaction between coinage metals and boron atoms have been described,^19^ to the best of our knowledge, the B:→M (M = Cu, Ag, Au) bonding mode is still unknown to date. We were interested in introducing **1** as the ligand onto coinage metals, not only for the fundamental curiosity in developing the novel B:→M bonding, but also considering their further application since a variety of coinage metal complexes exhibit catalytic activity in organic transformation.^20^ First, we investigated the reaction between **1** and coinage metal chlorides MCl (M = Cu, Ag, Au). Addition of two equivalents of MCl to a THF solution of **1** at room temperature immediately afforded a black precipitate, presumably metal (0), indicating the reduction of coinage metal chlorides by **1**. In fact, quantitative formation of the resulting boronium **5** was observed (Scheme 4, top), and **5** was fully characterized by HRMS and NMR spectroscopy which are identical to previous data for **5** with trifluoromethanesulfonate (^–^OTf) as the counter anion.^13^ We reasoned that the presence of a strong σ-donating ligand on the M(I) would increase the electron density at the metal center, which will prevent the electron transfer from **1** to the metals. To examine this hypothesis, we chose N-heterocyclic carbene–gold chloride (IPr)AuCl as a precursor. A THF solution of **1** and (IPr)AuCl was stirred at ambient temperature for 10 min. As expected, no precipitation of black metal was detected, and a clear yellow solution was generated instead. After evaporation of the solvent *in vacuo* and washing of the residue with toluene, **6** was obtained as a white solid in 95% yield (Scheme 4, bottom). In the ^11^B NMR spectrum of **6**, an upfield shift from the precursor was observed at –15.1 ppm, which is in line with the formation of a new gold complex bearing the L_2_PhB: ligand. Recrystallization from a saturated THF solution of **6** at –25 °C under argon afforded single crystals, and the solid-state structure of cationic gold(i) complex **6** was confirmed by a single-crystal X-ray diffraction study (Fig. 6).^14^ Both the L_2_PhB: and IPr ligands coordinate to the cationic gold center heteroleptically, and the B–Au–C(carbene) angle (179.1(5)°) deviates only slightly from linearity. The Au–B distance of 2.170(12) Å is significantly shorter than those (2.31–2.66 Å) reported for gold–borane complexes;^21^ however, it correlates well with those of gold–boryl and boride complexes **Ia**, **IIa**, **IVa**, **IVb**, and **Va** (2.07–2.21 Å). It is also in good agreement with the calculated result (2.176 Å). Significantly, a slight elongation of the Au–C(carbene) bond (2.068(10) Å) with respect to that in [(IPr)Au(PPh_3_)]BF_4_ (2.039(5) Å)^22^ was observed which suggests a *trans* influence of L_2_PhB: comparable with triphenylphosphine. To gain further insight into the bonding nature of the Au–B bond, geometry optimization, NBO analysis, and NPA calculation for **6** were performed. Fig. 7 shows the HOMO of **6** which mainly corresponds to the Au–B bond. The Au–B bond of **6** is formed by coordination of electrons in a high-p-character hybrid orbital (s: 16.08%, p: 83.89%) of the boron atom to an unoccupied orbital of the cationic gold atom (s: 84.51%, p: 0.18%, d: 15.29%). Remarkably, the WBI value of the Au–B bond (0.48) is even larger than that of the Au–C(carbene) bond (0.46), supporting the strong σ-donating property of the L_2_PhB: ligand. Indeed, NPA revealed that a charge transfer of 0.52*e* occurs from the L_2_PhB: fragment to the cationic gold(i) whereas a lesser charge (0.36*e*) is transferred from the IPr fragment to the gold(i) center. The calculated bond dissociation energy (BDE) for the B:→Au and Au←:C_(carbene)_ bonds in **6** are 92.6 and 72.0 kcal mol^–1^, respectively. The solid-state IR spectrum of **6** displays Au–B stretching vibrations at 570 cm^–1^, that are consistent with the computational result (mode 89: 582 cm^–1^, see the ESI†).

**Scheme 5:**
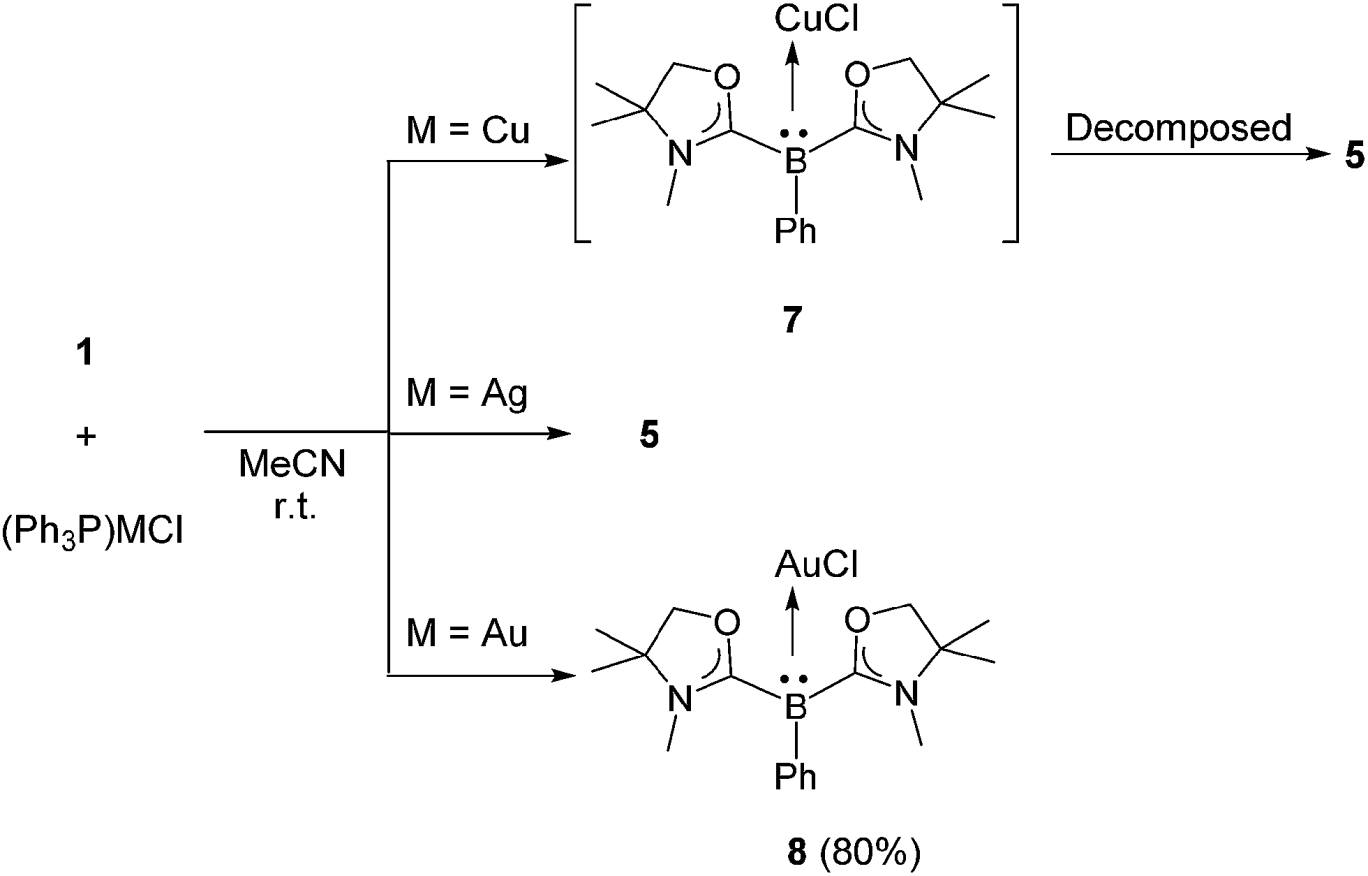
Reactions of **1** with (Ph_3_P)MCl (M = Cu, Ag, Au).

Reactions with (IPr)CuCl and (IPr)AgCl were also examined. A CD_3_CN solution of **1** and (IPr)CuCl was prepared in a J. Young NMR tube. ^1^H and ^11^B NMR spectra were measured immediately, and showed unidentified complex mixtures. Meanwhile, a reaction mixture of **1** with an equivalent of (IPr)AgCl in CD_3_CN displayed a characteristic signal at –15.6 ppm in the ^11^B NMR spectrum, presumably indicating the generation of [(L_2_PhB)Ag(IPr)]Cl. However, this intermediate was not thermally stable: thus, it decomposed completely after 4 h at ambient temperature, and the formation of [(IPr)_2_Ag]Cl was confirmed concomitant with the reproduction of **1** (see the ESI†). The reactivity of **1** towards triphenylphosphine-ligated group 11 metal chlorides (Ph_3_P)MCl (M = Cu, Ag, Au) was also investigated. A mixture of **1** and an equivalent of (Ph_3_P)CuCl was dissolved in CD_3_CN and the reaction was monitored by NMR spectroscopy (see the ESI†). In the ^11^B NMR spectrum, a singlet appeared at –16.8 ppm which is in line with the formation of (L_2_PhB)CuCl **7** (Scheme 5, top). In fact, a quantitative generation of free Ph_3_P was confirmed by ^31^P NMR. However, compound **7** gradually decomposed to **5** within 24 h at room temperature. Reaction of **1** with (Ph_3_P)AgCl afforded **5** directly, and no corresponding Ag complex intermediate was detected even when the reaction was conducted at low temperature (Scheme 5, middle). Interestingly, a contrasting result was obtained when (Ph_3_P)AuCl was employed. An acetonitrile solution of **1** and (Ph_3_P)AuCl was stirred at ambient temperature for 10 min. After removing the solvent *in vacuo* and washing the residue with *n*-hexane, the neutral gold chloride complex **8** was obtained as a white solid in 80% yield. Formation of **8** *via* the smooth replacement of triphenylphosphine by **1** on the gold atom demonstrates the strong nucleophilicity of the L_2_PhB: ligand **1**. Note that a variety of gold complexes featuring strong σ-donating neutral ligands are widely used as efficient catalysts in organic synthesis.^23^ The ^11^B NMR of **8** displays a sharp singlet at –18.6 ppm which is shifted upfield compared to that of **1** (–1.1 ppm). The complex **8** is not only moisture sensitive but light sensitive, and quickly decomposes if exposed to air or light.

**Fig. 8:**
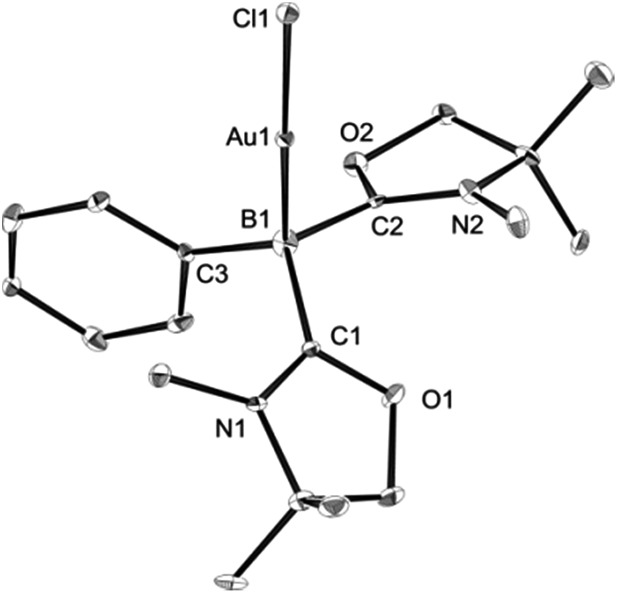
Solid-state structure of **8** (hydrogen atoms are omitted for clarity). Thermal ellipsoids are set at the 50% probability level.

**Fig. 9:**
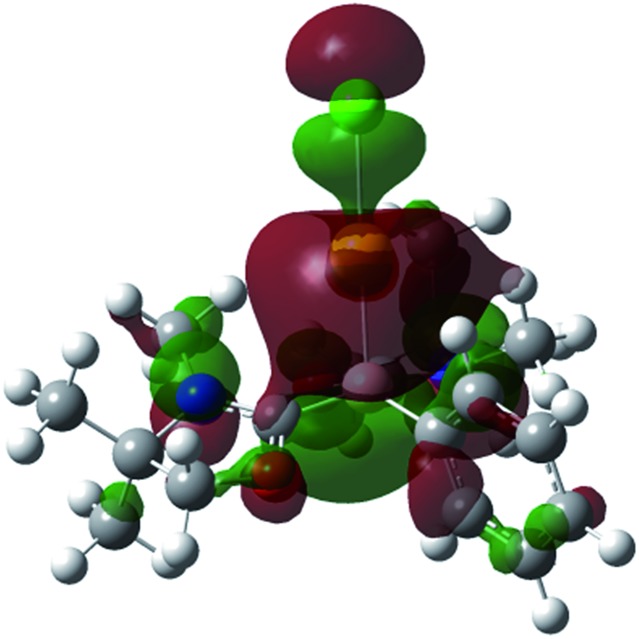
Plot of the HOMO (–6.779 eV) of **8** (calculated at the M05-2X/6-311G(d,p) level of theory, with the LANL2TZ(f) pseudopotential applied for the gold atom).

Single crystals of **8** were obtained from an acetonitrile/toluene mixed solvent at room temperature, and X-ray diffraction analysis revealed that **8** features an almost linear geometry (B1–Au1–Cl1: 175.2(4)°), and the tetracoordinate boron is bound to the gold(i) center in an η^1^-fashion (Fig. 8).^14^ The Au–B distance of 2.166(14) Å is nearly identical to that of **6**. These parameters are in good accordance with the calculated results for the model compounds (Ph_3_P)_2_(BH)AuCl and (NHC_Me_)_2_(BH)AuCl.^24^ We performed quantum chemical calculations to investigate the property of the Au–B bond in **8**. The HOMO of **8** displays the coordination of electrons of the boron to the gold atom (Fig. 9). NBO analysis gave a Wiberg bond index (WBI) value of the Au–B bond (0.50), which is formed from the high-p-character hybrid orbital (s: 15.49%, p: 84.47%) of the boron atom and mainly the s-orbital of the gold atom (s: 84.66%, p: 1.72%, d: 13.60%). Natural Population Analysis (NPA) indicates an overall charge transfer of 0.43*e* from the L_2_PhB: fragment to the gold chloride. The calculated BDE of the donor–acceptor bond between **1** and the gold chloride in **8** is 78.4 kcal mol^–1^, which is comparable to that (77.3 kcal mol^–1^) of (Ph_3_P)_2_(BH)AuCl.^24^ Comparatively, much lower BDEs were estimated for the corresponding bonds in **1**-CuCl (64.3 kcal mol^–1^) and **1**-AgCl (55.8 kcal mol^–1^), which may account for the instability of compound **7**. An analogous trend of the bond energies for the metal fragments (Au > Cu > Ag) was also found in their carbene, silylene and germylene complexes.^25^ In the solid state IR spectrum of **8**, a characteristic peak was observed at 586 cm^–1^, which is assigned to a Au–B stretch based on computational results (mode 41: 577 cm^–1^, see the ESI†).

### Preliminary investigation of catalysis with complex **8**

Next, we turned our attention to the potential ability of complex **8** as a catalyst. We chose the hydroamination reaction between aniline **9a** and phenylacetylene **10a** as a model reaction. A brief screening of solvents (Table 1, entries 1–5) revealed that benzene is suitable for the reaction; an imine derivative **11aa** was obtained in 94% yield after 12 h at room temperature (entry 5). During this reaction we observed a main signal in the ^11^B NMR spectrum at –16.1 ppm, in addition to the peak at –16.6 for B(C_6_F_5_)_4_ and a small peak at –19.3 ppm. Control reactions suggest that the main signal may correspond to either (L_2_PhB)Au(C≡CPh) or σ,π-acetylide complex [(L_2_PhB)Au]_2_(C≡CPh)·B(C_6_F_5_)_4_, proposing the resting state of the catalyst (see the ESI†). Hence, this result supports the innocence of colloidal gold, demonstrating the essential role of **8** in the catalytic reaction. With AgOTf as an additive, little formation of **11aa** (<5%) was observed (entry 6).

**Table 1:** Optimization of the reaction conditions^*a*^

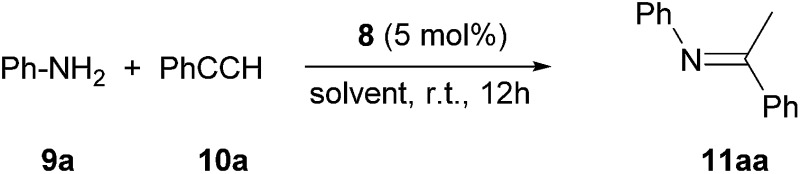			
Entry	Additive (5 mol%)	Solvent	Yield^*b*^ (%)
1	KBAr^F^_4_	CD_3_CN	9
2	KBAr^F^_4_	THF-D_8_	12
3	KBAr^F^_4_	CDCl_3_	73
4	KBAr^F^_4_	CD_2_Cl_2_	74
5	KBAr^F^_4_	C_6_D_6_	94
6	AgOTf	C_6_D_6_	<5

^*a*^Reaction conditions: **9a** (0.5 mmol), **10a** (0.5 mmol), solvent (0.5 mL).

^*b*^Yields were determined by ^1^H NMR spectroscopy using 1,3,5-trimethoxy benzene as an internal standard.

**Scheme 6:**
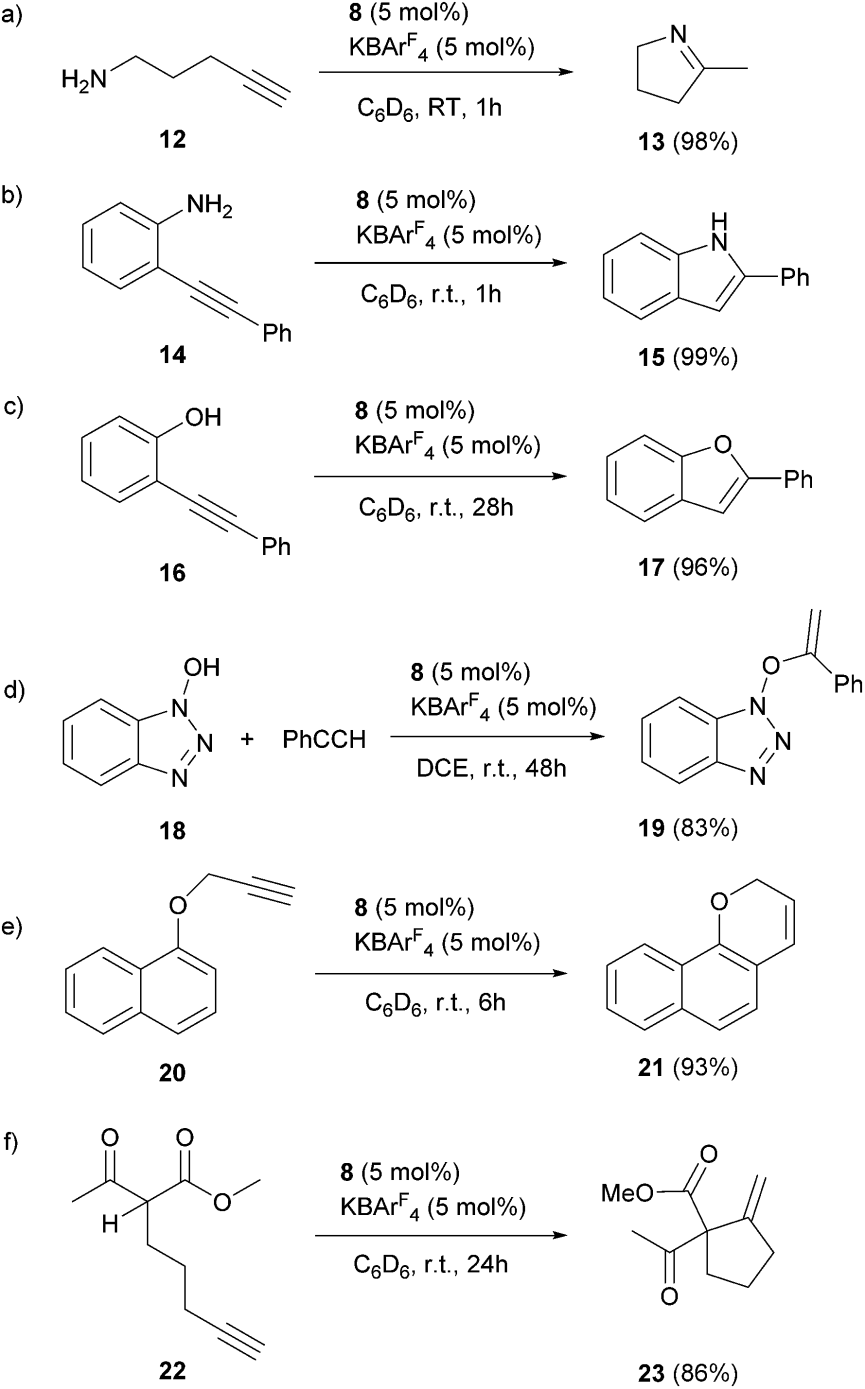
Catalytic addition of X–H (X = N, O, C) bonds to alkynes.

The scope of the catalytic reaction was briefly examined with various primary amine and terminal alkyne derivatives at ambient temperature (Table 2). The addition reactions of aniline **9a**, *p*-anisidine **9b** or 3,5-dimethylaniline **9c** to phenylacetylene **10a** or 4-ethynylanisole **10b** were effectively catalyzed to afford the corresponding imines in excellent yields: **11aa** (98%), **11ab** (93%), **11ba** (90%), **11bb** (94%) and **11ca** (91%) (entries 1, 2, 4, 5, 7). Lower activity was observed when the sterically hindered amine substrates **9d, e** were used (entries 8–10); *i.e.* from the reaction of **9e** and **10b**, only moderate formation of the corresponding imine **11eb** (44%) was observed after 72 h (entry 10). Although a longer reaction time was required, the catalyst **8** was also effective for the hydroamination with more challenging electron-poor alkynes, such as 1-bromo-4-ethynylbenzene **10c**. Thus, imine derivatives **11ac**, **11bc** and **11ec** were slowly formed from the reaction of **10c** and the corresponding amines (**9a**, **9b** and **9e**) in low to good yields (entries 3, 6 and 11, respectively). Not surprisingly, complex **8** also catalyzed intramolecular hydroamination (Scheme 6a and b), in which N-heterocyclic products **13** and **15** were obtained quantitatively. We also tested O–H additions to alkynes with a catalytic amount of **8** (5 mol%). At room temperature, compound **16** was smoothly converted to 2-phenylbenzofuran **17** (96%) (Scheme 6c). When *N*-hydroxy benzotriazole **18** was treated with phenylacetylene, **19** was formed in good yield (83%) *via* an intermolecular O–H addition (Scheme 6d). Because selective C–H activation as well as the formation of C–C bonds is significant in organic synthesis, we also investigated the catalytic transformation of compounds **20** and **22**. In the reaction with 1-naphthalenyl propargyl ether **20**, addition of a C(sp^2^)–H bond across the C–C triple bond took place which afforded a benzo[*h*]chromene derivative **21** in 93% yield after 6 h (Scheme 6e). The cyclisation reaction of compound **22** also occurred effectively *via* activation of a C(sp^3^)–H bond, and β-ketoester **23** was obtained in good yield (86%) (Scheme 6f). Since it has been already demonstrated that with colloidal gold, higher temperature, irradiation of light, or longer reaction times are required to conduct the relevant reactions,^26^ its involvement may be ruled out here. Note that some of the results shown here were comparable to or even better than those with (Ph_3_P)AuCl as a precatalyst under similar conditions.^27^ It is also noteworthy that these results demonstrated the first application of metal complexes featuring a neutral boron-based ligand in catalysis.

**Table 2:** Catalytic hydroamination of alkynes with primary amines^*a*^

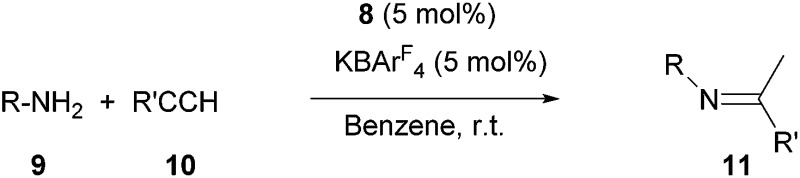				
Entry	**9**	**10**	Time (h)	**11**, Yield^*b*^ (%)
1	**9a** (R = Ph)	**10a** (R′ = Ph)	24	**11aa**, 98
2	**9a**	**10b** (R′ = 4-MeOC_6_H_4_)	24	**11ab**, 93
3	**9a**	**10c** (R′ = 4-BrC_6_H_4_)	72	**11ac**, 86
4	**9b** (R = 4-MeOC_6_H_4_)	**10a**	36	**11ba**, 90
5	**9a**	**10b**	36	**11bb**, 94
6	**9a**	**10c**	72	**11bc**, 54
7	**9c** (R = 3,5-Me_2_C_6_H_3_)	**10a**	36	**11ca**, 91
8	**9d** (R = 2,4,6-Me_3_C_6_H_2_)	**10b**	72	**11db**, 73
9	**9e** (R = 2,6-^i^Pr_2_C_6_H_3_)	**10a**	72	**11ea**, 46
10	**9e**	**10b**	72	**11eb**, 44
11	**9e**	**10c**	72	**11ec**, 30

^*a*^Reaction conditions: **9** (2 mmol), **10** (2 mmol) benzene (3 mL).

^*b*^Isolated yields.

## Conclusions

In conclusion, we have demonstrated the diverse reactivity of L_2_PhB: (**1**) towards metal precursors. Addition of hard acidic LiOTf to **1** induced coordination of the oxygen atoms in the oxazol-2-ylidenes to the Li atoms, and the unique compound **2** containing two L_2_PhB: moieties bridged *via* a cyclic Li(OTf)_2_Li unit was obtained. Reaction of **1** with [RhCl(COD)]_2_ afforded the hitherto unknown zwitterionic complex **3** involving an anionic rhodium and a boronium cation whereas iridium borane complex **4** featuring a 3c-2e Ir–H–B bond was isolated from reaction with [IrCl(COD)]_2_. In the formation of complexes **3** and **4**, deprotonation of the metal hydrides by the boron atom is proposed, which illustrates the strongly basic property of the boron center in **1**. Direct complexation between **1** and gold chloride derivatives bearing carbene or phosphine ligands produced the first isolable gold complexes **6** and **8** featuring B:→Au bonding. Depending on the ligand on the gold chloride precursors, either a cationic or a neutral gold(i) complex supported by the L_2_PhB: ligand can be obtained selectively *via* Au–Cl bond dissociation or ligand exchange, respectively. X-ray diffraction analysis and computational studies revealed the unique Au–B bonding formed by the coordination of lone-pair electrons on the boron atom to the gold center as well as the strong σ-donating property of the L_2_PhB: ligand. Complex **8** showed good catalytic activity when employed as a precatalyst for both intra- and inter-molecular X–H (X = N, O, C) addition to alkynes. Further developments of metal complexes bearing **1** and its application in catalysis are currently under investigation in our laboratory.
