# Supplementary material for: Diverse reactivity of a tricoordinate organoboron L2PhB: (L = oxazol-2-ylidene) towards alkali metal, group 9 metal, and coinage metal precursors
Source: Chem Sci. 2015 Feb 23;6(5):2893–902. doi: 10.1039/c5sc00404g (PMC5655907; doi:10.1039/c5sc00404g)
Supplement: Supplementary file 1 [file SC-006-C5SC00404G-s001.pdf]

Electronic Supplementary Material (ESI) for Chemical Science.
This journal is © The Royal Society of Chemistry 2015

Supplementary Information

Diverse Reactivity of A Tricoordinate Organoboron L_2PhB: (L = oxazol-2-ylidene) towards Alkali Metal, Group 9 Metal, and Coinage Metal Precursors

Lingbing Kong, Rakesh Ganguly,Yongxin Li, and Rei Kinjo*

This PDF file includes:

Supplementary Text

Supplementary Figs. 1 to 34

Supplementary Note 1

Supplementary Tables 1 to 7

Supplementary References

Method

General. All reactions were performed under an atmosphere of argon by using standard Schlenk or dry box techniques; solvents were dried over Na metal, K metal or CaH_2. ^1H, ^{13}C, ^{11}B, ^7Li, and ^{19}F NMR spectra were obtained with AVIII 400MHz BBFO1 spectrometer at 298 K. NMR multiplicities are abbreviated as follows: s = singlet, d = doublet, t = triplet, m = multiplet, br = broad signal. Coupling constants *J* are given in Hz. Electrospray ionization (ESI) mass spectra were obtained at the Mass Spectrometry Laboratory at the Division of Chemistry and Biological Chemistry, Nanyang Technological University. Melting points were measured with an OpticMelt Stanford Research System. Fourier transform infrared (FT-IR) spectra were recorded on a SHIMADZU IRPrestige-21 spectrometer.

Synthesis of 2: A toluene solution (30 mL) of **1** (157 mg, 0.5 mmol) was added into lithium trifluoromethanesulfonate (78 mg, 0.5 mmol) at room temperature. The resulting suspension was stirred for 2 days at room temperature. All volatiles were removed under vacuum and washed with n-hexane to afford a yellow powder of **2** in 90 % yield. Mp: 146 °C (dec); ^1H NMR (400 MHz, C_6D_6): δ = 0.71 (s, 24H, CH_3), 2.42 (s, 12H, N-CH_3), 3.64 (s, 8H, CH_2), 7.22 (br, 2H, *p*-C*H*), 7.37 (br, 4H, *m*-C*H*), 7.87 (br, 4H, *o*-C*H*); ^{13}C NMR (100.56 MHz, C_6D_6): δ = 22.2 (CH_3), 30.4 (N-CH_3), 60.6 (q), 77.4 (CH_2), 126.5 (*p*), 128.2 (*m*), 138.1 (*o*), B-*C* and F-*C* were not observed; ^7Li NMR (116.6 MHz, C_6D_6): δ = 0.62 (s); ^{11}B NMR (128.3 MHz, C_6D_6): δ = 1.74 (s); ^{19}F NMR (376.3 MHz, C_6D_6): δ = -78.0 (s); HRMS (ESI): *m/z* calcd for $C_{19}H_{28}BF_3LiN_2O_5S$: 471.1924 $[(M+H)]^+$; found: 471.1914.

Synthesis of 3: 10 mL THF was added to the mixture of **1** (63 mg, 0.2 mmol) and $[RhCl(COD)]_2$ (50 mg, 0.1 mmol) and the resulting solution was stirred for 10 min. After

removal of solvent, the residue was washed with n-hexane and recrystalized from toluene to afford yellow crystals of **3** (51 mg, 45% yield). Mp: 105 °C (Dec). ^{1}H NMR (400 MHz, C_6D_6): δ = 0.24 (s, 3H, CH_3), 0.77 (s, 3H, CH_3), 0.91 (s, 3H, CH_3), 1.71 (s, 3H, CH_3), 1.94 (m, 4H, COD-CH_2), 2.40 (m, 5H, COD-CH_2 and Rh-CH_2), 2.97 (s, 3H, N-CH_3), 3.24 (m, 1H, COD-C*H*), 3.38 (m, 1H, COD-C*H*), 3.45 (t, 2H, ^2J = 8.1 Hz, CH_2), 3.90 (d, 1H, ^2J = 8.8 Hz, CH_2), 4.08 (d, 1H, ^2J = 8.8 Hz, CH_2), 4.18 (br, 1H, COD-C*H*2), 4.96 (m, 1H, COD-C*H*), 5.15 (m, 1H, COD-C*H*), 7.23 (t, 1H, ^3J = 7.4 Hz, *p*-C_6H_5), 7.40 (t, 2H, ^3J = 7.4 Hz, *m*-C_6H_5), 7.86 (2H, d, ^3J = 7.1 Hz, *o*-C_6H_5); ^{13}C NMR (100.56 MHz, C_6D_6): δ = 22.5 (*C*H$_3$), 24.0 (*C*H$_3$), 25.3 (*C*H$_3$), 25.4 (*C*H$_3$), 29.4 (N-*C*H$_3$), 30.2 (COD-*C*H$_2$), 30.6 (COD-*C*H$_2$), 33.2 (COD-*C*H$_2$), 33.3 (COD-*C*H$_2$), 39.7 (d, J_{RhC} = 31.8 Hz, Rh-*C*H$_2$), 63.7 (q), 65.1 (q), 67.4 (d, J_{RhC} = 16.6 Hz, COD-*C*H), 67.8 (d, J_{RhC} = 16.3 Hz, COD-*C*H), 79.2 (*C*H$_2$), 80.8 (*C*H$_2$), 87.4 (d, J_{RhC} = 7.5 Hz, COD-*C*H), 87.9 (d, J_{RhC} = 7.4 Hz, COD-*C*H), 126.1 (*p*), 127.9 (*m*), 135.5(*o*); ^{11}B NMR (128.3 MHz, C_6D_6): δ = -21.0 (d, J_{BH} = 34.0 Hz); HRMS (ESI): *m/z* calcd for $C_{26}H_{40}BClN_2O_2Rh$: 561.1779 [$(M+H)$]$^+$; found: 561.1906.

Synthesis of 4: 10 mL THF was added to the mixture of **1** (63 mg, 0.2 mmol) and $[IrCl(COD)]_2$ (67 mg, 0.1 mmol) and the resulting solution was stirred for 10 min. After removal of solvent, the residue was washed with n-hexane and recrystalized from toluene and THF to afford colorless crystals of **4** (100 mg, 80% yield). Mp: 145 °C. ^{1}H NMR (400 MHz, CD_2Cl_2): δ = -7.56 (br, 1H, B-H), 1.31 (m, 1H, COD-CH_2), 1.37 (s, 3H, CH_3), 1.41 (s, 3H, CH_3), 1.43 (s, 3H, CH_3), 1.44 (s, 3H, CH_3), 1.72 (m, 1.5H, COD-CH_2), 1.83 (m, 2H, COD-CH_2), 2.03 (m, 1H, COD-C*H*), 2.22 (m, 1.5H, COD-CH_2), 2.66 (m, 1H, COD-CH_2), 2.81 (m, 1H, COD-CH_2), 3.43 (s, 3H, N-CH_3), 3.84 (d, 1H, ^2J = 11.6 Hz, Ir-CH_2), 4.24 (d,

1H, ^2J = 8.8 Hz, CH_2), 4.31 (d, 1H, ^2J = 8.8 Hz, CH_2), 4.44 (d, 1H, ^2J = 9.1 Hz, CH_2), 4.60 (d, 1H, ^2J = 9.1 Hz, CH_2), 4.68 (d, 1H, ^2J = 11.6 Hz, Ir-CH_2), 5.17 (m, 1H, COD-C*H*), 5.66 (m, 1H, COD-C*H*), 7.12 (t, 1H, ^3J = 7.2 Hz, *p*-C_6H_5); 7.20 (t, 2H, ^3J = 7.4 Hz, *m*-C_6H_5); 7.41 (2H, d, ^3J = 6.7 Hz, *o*-C_6H_5); ^{13}C NMR (100.56 MHz, CD_2Cl_2): δ = 13.7 (Ir-*C*H), 24.1 (*C*H_3), 24.5 (*C*H_3), 24.6 (*C*H_3), 24.8 (COD-*C*H_2), 25.2 (*C*H_3), 26.3 (COD-*C*H_2), 26.7 (COD-*C*H_2), 27.1 (COD-*C*H_2), 30.7 (N-*C*H_3), 43.1 (Ir-*C*H_2), 64.2 (q), 64.5 (q), 81.1 (*C*H_2), 85.5 (*C*H_2), 88.4 (COD-*C*H), 93.2 (COD-*C*H), 125.6 (*p*), 127.2 (*m*), 133.4 (*o*), 190.7 (carbene-*C*); ^{11}B NMR (128.3 MHz, CD_2Cl_2): δ = -22.8 (br); HRMS (ESI): *m/z* calcd for $C_{26}H_{39}BIrN_2O_2$: 615.2734 $[(M - Cl)]^+$; found: 615.2726; IR (KBr, cm^{-1}): IR (KBr, cm^{-1}): 1618.28 (Ir–H–B).

Synthesis of 5: A THF solution (5 mL) of **1** (16 mg, 0.05 mmol) was added into coinage metal chlorides (0.1 mmol) at room temperature. Color changed immediately from orange to colorless with some black metal precipitated. After the metal was filtered off, all volatiles were removed under vacuum to afford a colorless powder of **5** in 98 % yield. Mp: 146 °C (dec); ^{1}H NMR (400 MHz, THF-d_8): δ = 1.48 (s, 6H, CH_3), 1.56 (s, 6 H, CH_3), 3.20 (s, 6H, N-CH_3), 4.73 (d, 2H, ^2J = 9.7 Hz, CH_2), 4.81 (d, 2H, ^2J = 9.7 Hz, CH_2), 7.23 (t, 1H, ^3J = 7.3 Hz, *p*-C*H*), 7.30 (t, 2H, ^3J = 7.4 Hz, *m*-C*H*), 7.49 (2H, d, ^3J = 6.8 Hz, *o*-C*H*); ^{13}C NMR (100.56 MHz, THF-d_8): δ = 23.6 (*C*H_3), 23.9 (*C*H_3), 29.9 (N-*C*H_3), 68.1 (q), 82.6 (*C*H_2), 128.2 (*p*), 128.5 (*m*), 133.1 (*o*), B-*C* was not observed; ^{11}B NMR (128.3 MHz, THF-d_8): δ = –11.2 (s); HRMS (ESI): *m/z* calcd for $C_{18}H_{27}BClN_2O_2$: 349.1854 $[(M)]^+$; found: 349.1860.

Synthesis of 6: A solution of **1** (157 mg, 0.5 mmol) in THF (10 mL) was added to a solution of (IPr)AuCl (310 mg, 0.5 mmol) in THF (10 mL) at room temperature under dark conditions. The mixture was stirred for 10 min. All volatiles were removed in vacuo. After

the residue was washed with toluene (10 mL), the solvent was removed under vacuum to afford **6** as a white solid in 95% yield. Mp: 212 °C (dec). ^{1}H NMR (400 MHz, CD_2Cl_2): δ = 1.07 (s, 6H, C*H*$_3$), 1.10 (s, 6H, C*H*$_3$), 1.22 (d, 12H, ^3J = 6.9 Hz, (C*H*$_3$)$_2$CH), 1.28 (d, 12H, ^3J = 6.9 Hz, (C*H*$_3$)$_2$CH), 2.30 (s, 6H, N-C*H*$_3$), 2.66 (sept, 4H, ^3J = 6.9 Hz, (CH$_3$)$_2$C*H*), 3.96 (d, 2H, ^2J = 9.0 Hz, C*H*$_2$), 4.10 (d, 2H, ^2J = 9.0 Hz, C*H*$_2$), 6.97 (br, 5H, Ph-*H*), 7.24 (s, 2H, N-C*H*), 7.30 (d, 4H, ^3J = 7.8 Hz, Dipp-*H*), 7.51 (t, 2H, ^3J = 7.8 Hz, Dipp-*H*); ^{13}C NMR (100.56 MHz, CD_2Cl_2): δ = 24.0 (*C*H$_3$), 24.1 ((*C*H$_3$)$_2$CH), 24.2 (*C*H$_3$), 24.7 ((*C*H$_3$)$_2$CH), 29.1 ((CH$_3$)$_2$*C*H), 29.3 (N-*C*H$_3$), 64.0 (q), 79.9 (*C*H$_2$), 123.8 (*C*H), 124.4 (*C*H), 125.2 (*C*H), 127.3 (*C*H), 130.8 (*C*H), 135.1 (q), 138.5 (*C*H), 146.3 (q), carbene carbon and B-*C* were not observed; ^{11}B NMR (128.3 MHz, CD_2Cl_2): δ = –15.1 (s); HRMS (ESI): *m/z* calcd for $C_{45}H_{63}AuBN_4O_2$: 899.4710 [(*M*)]$^+$; found: 899.4714; IR (KBr, cm^{-1}): IR (KBr, cm^{-1}): 570.93 (Au–B).

Synthesis of compound 8: A solution of **1** (78 mg, 0.25 mmol) in acetonitrile (10 mL) was added dropwise to a solution of (Ph_3P)AuCl (124 mg, 0.25 mmol) in acetonitrile (10 mL) at room temperature under dark conditions. The mixture was stirred for 10 min. All volatiles were removed in vacuo. After the residue was washed with 20 mL n-hexane, the solvent was removed under vacuum to give **7** as a colorless powder in 80% yield. Mp: 118 °C (dec). ^{1}H NMR (400 MHz, CD_3CN): δ = 1.29 (s, 6H, C*H*$_3$), 1.33 (s, 6 H, C*H*$_3$), 3.01 (s, 6H, N-C*H*$_3$), 4.27 (d, 2H, ^2J = 9.3 Hz, C*H*$_2$), 4.30 (d, 4H, ^2J = 9.3 Hz, C*H*$_2$), 7.06 (t, 1H, ^3J = 7.3 Hz, *p*-C*H*), 7.14 (t, 2H, ^3J = 7.2 Hz, *m*-C*H*), 7.38 (d, 4H, ^3J = 5.6 Hz, *o*-C*H*); ^{13}C NMR (100.56 MHz, CD_3CN): δ = 23.7 (*C*H$_3$), 23.8 (*C*H$_3$), 65.5 (q), 80.7 (*C*H$_2$), 125.8 (*C*H), 127.9 (*C*H), 138.8 (*C*H), B-*C* was not observed; ^{11}B NMR (128.3 MHz, CD_3CN): δ = –18.6 (s); HRMS

(ESI): *m/z* calcd for $C_{18}H_{28}AuBClN_2O_2$: 547.1598 [(*M+H*)]$^+$; found: 547.1611; IR (KBr, cm^{-1}): 586.36 (Au–B).

General procedure for catalysis:

All the substrates were dried before used and known catalytically produced products were identified by comparison to their reported spectroscopic data (**11aa**[1], **11ab**[2], **11ac**[3], **11ba**[4], **11bb**[4], **11bc**[5], **11ca**[1], **11db**[6], **11ea**[7], **11eb**[6], **13**[8], **15**[9], **17**[10], **19**[11], **21**[12], **23**[13])

To a small screw-cap scintillation vial equipped with a magnetic stir bar and charged with a solution of substrates (1 eq) in benzene or DCE(0.65M) was added catalyst **8** (5 mol %) followed by $KBAr^F_4$ (5 mol%). The reaction mixture was then stirred at room temperature and monitored periodically by ^{1}H NMR. Upon completion, the imines products were purified *via* vacuum distillation (liquid residue) or recrystallization from methanol. The other products were purified on silica gel with the appropriate mixture of n-hexane and EtOAc.

11ec: Yellow solid. Mp:128 ^{o}C.^{1}H NMR (400 MHz, $CDCl_3$): δ = 1.12 (d, 6H, ^3J = 3.6 Hz, $(CH_3)_2$CH), 1.14 (d, 6 H, ^3J = 3.6 Hz, $(CH_3)_2$CH), 2.07 (s, 3H, N-CH_3), 2.69 (sept, 2H, ^3J = 3.6 Hz, $(CH_3)_2CH$), 7.07 (t, 1H, ^3J = 6.6 Hz,Dipp-*H*), 7.14 (d, 2H, ^3J = 7.0Hz,Dipp-*H*), 7.60 (d, 2H, ^3J = 8.6 Hz,Ar-*H*), 7.90 (d, 2H, ^3J = 8.6 Hz,Ar-*H*);^{13}C NMR (100.56 MHz, $CDCl_3$):δ = 18.0 (N-CH_3), 23.0 ($(CH_3)_2$CH), 23.3 ($(CH_3)_2$CH), 28.4 ($(CH_3)_2CH$), 123.1 (*C*H), 123.6 (*C*H), 125.2 (q), 128.9 (*C*H), 131.7 (*C*H), 136.1 (q), 138.0 (*q*), 146.6 (q), 163.9 (N=*C*); HRMS (ESI): *m/z* calcd for $C_{20}H_{25}NBr$: 358.1170 [(*M+H*)]$^+$; found: 358.1169.

Crystallographic details

X-ray data collection and structural refinement. Intensity data for compounds **2**, **3**, **4**, **6** and **8** was collected using a Bruker APEX II diffractometer. The crystals were measured at 103(2) K. The structure was solved by direct phase determination (SHELX-2013) and refined or all data by full-matrix least squares methods on F^2.[S14] All non-hydrogen atoms were subjected to anisotropic refinement. The hydrogen atoms were generated geometrically and allowed to ride in their respective parent atoms; they were assigned appropriate isotropic thermal parameters and included in the structure-factor calculations. CCDC; 1011533, 1011534, 1038665, 108666 and 1038667 contains the supplementary crystallographic data for this paper. The data can be obtained free of charge from the Cambridge Crystallography Data Center viawww.ccdc.cam.ac.uk/data_request/cif.

Computational details

Gaussian 09 was used for all density functional theory (DFT) calculations.[S15] Geometry optimization and frequency calculations, Natural bond order (NBO) analysis of compounds**2**, **3**, **4**, **6** and **8** were performed at the M052x/6-311G(d,p) level of theory, with the LANL2TZ(f) pseudo-potential applied for the metal atoms. Calculated bond dissociation energies D_e [kcal mol^{-1}] of compounds **6**, **7**, **8**, **1·AgCl** and their subunits were performed at the M052x/6-311G(d,p) level of theory, with the LANL2TZ(f) pseudo-potential applied for the metal atoms.

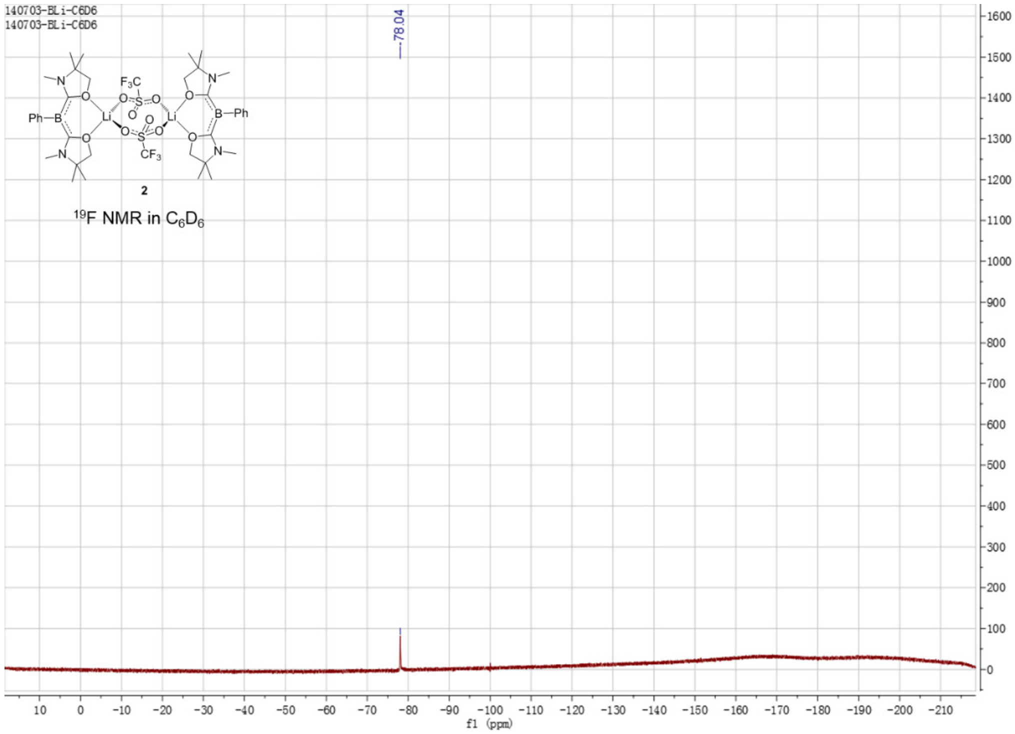

Supplementary Fig. 1^{19}F NMR spectrum of **2**.

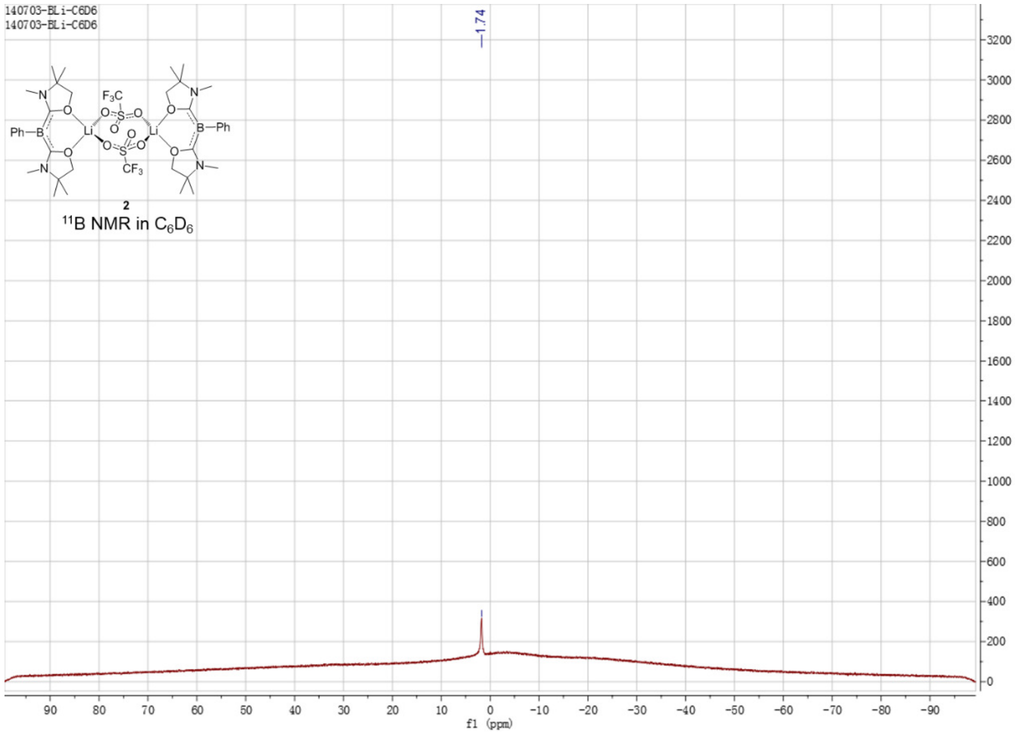

Supplementary Fig. 2 ^{11}B NMR spectrum of **2**.

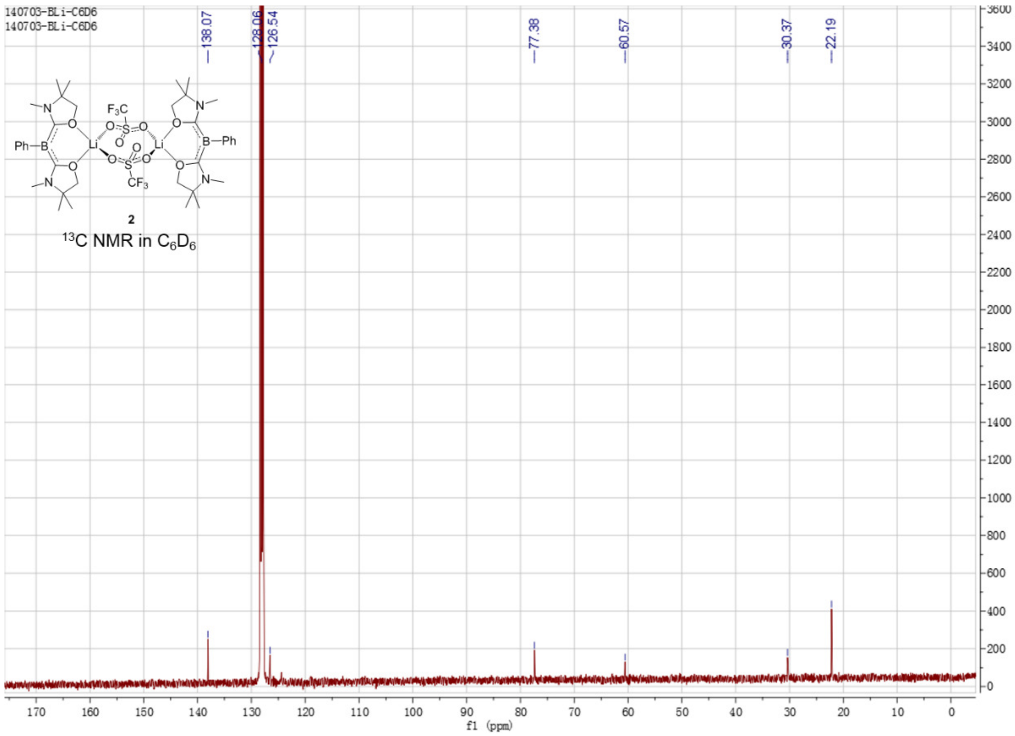

Supplementary Fig. 3 ^{13}C NMR spectrum of **2**.

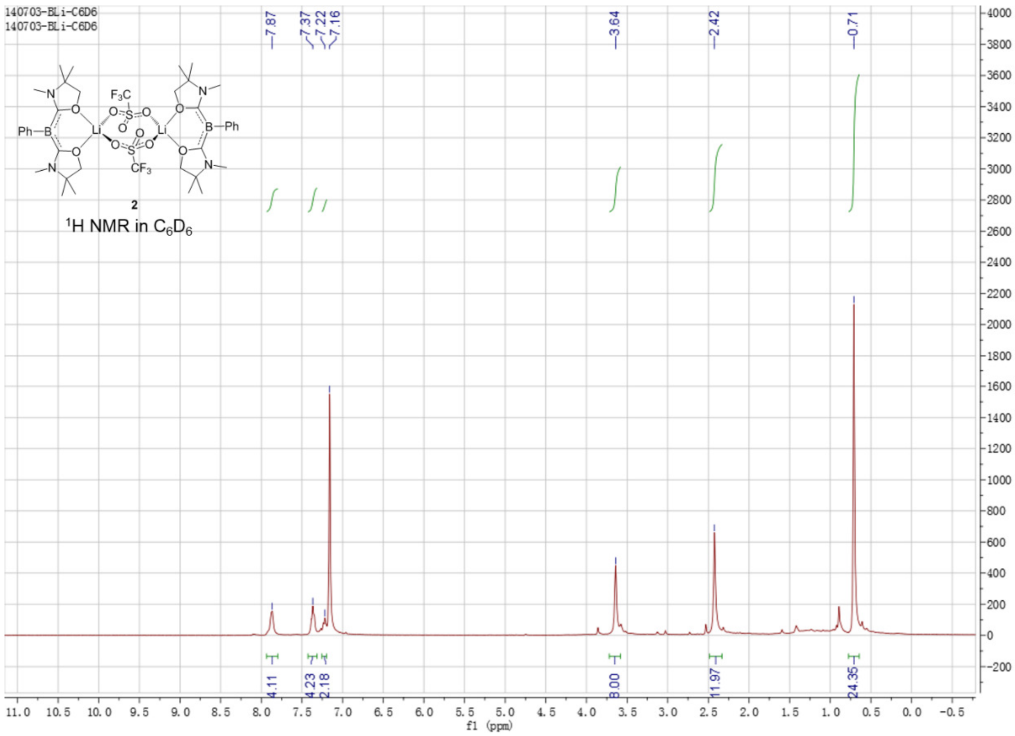

Supplementary Fig. 4 ^{1}H NMR spectrum of **2**.

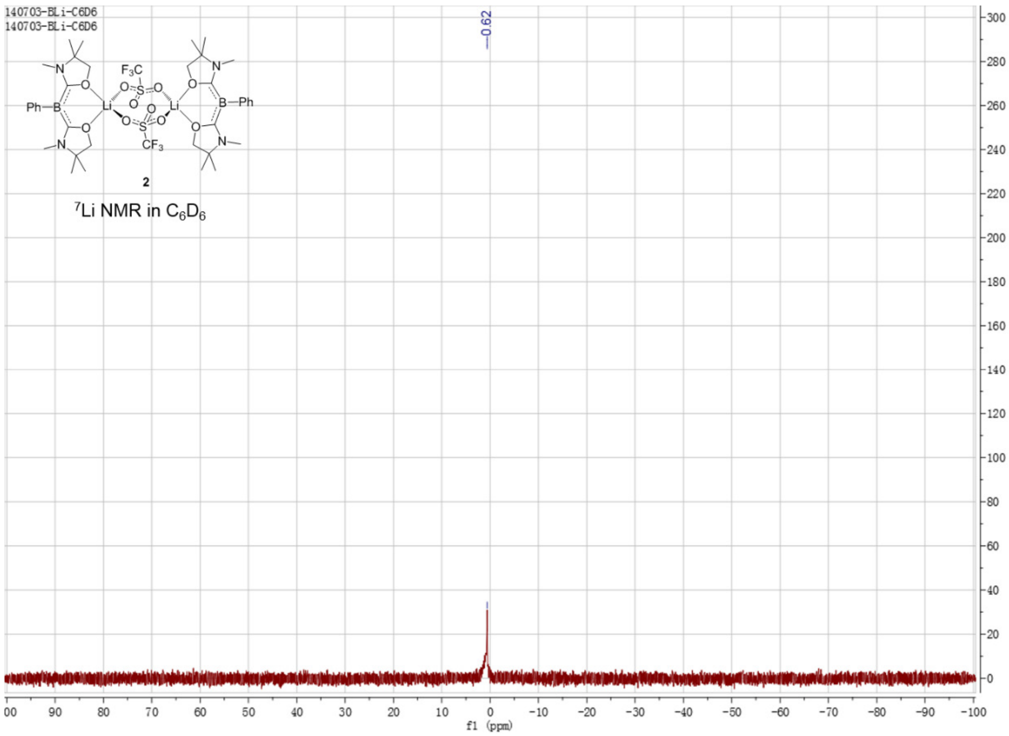

Supplementary Fig. 5 ^{7}Li NMR spectrum of **2**.

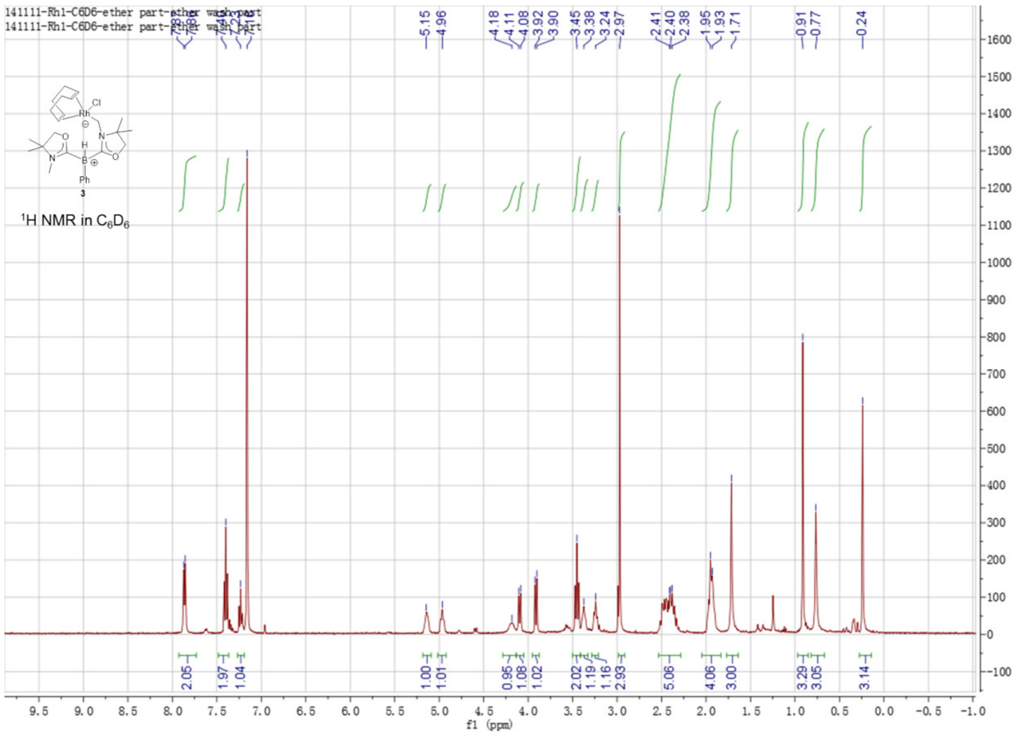

Supplementary Fig. 6 ^{1}H NMR spectrum of **3**.

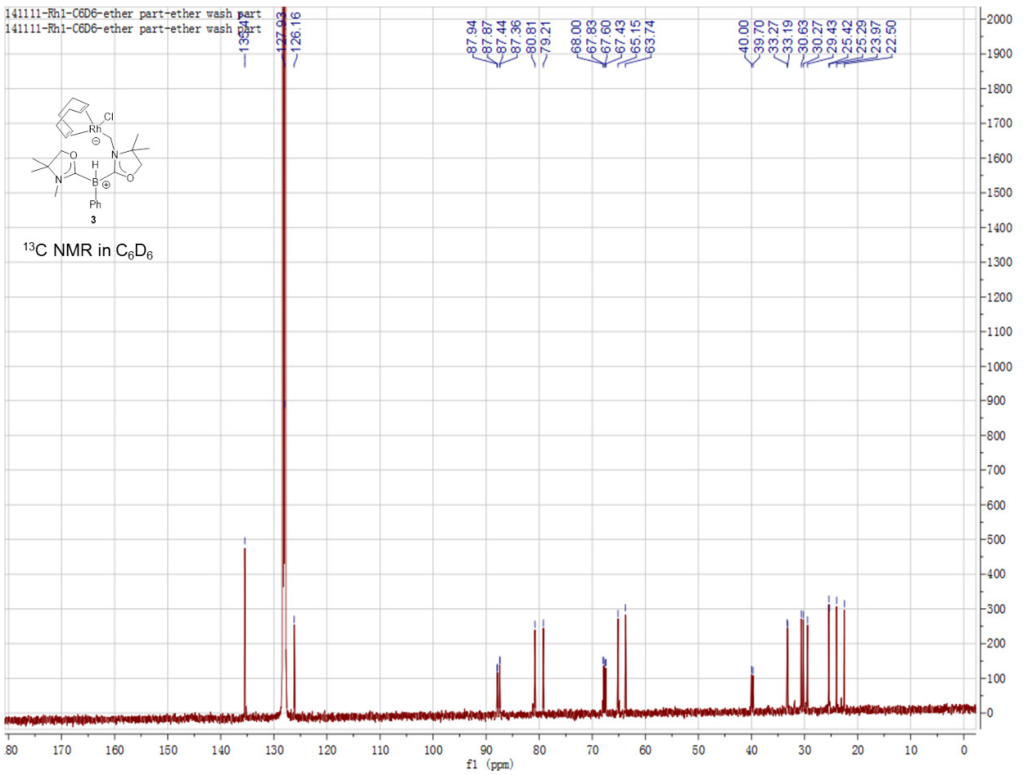

Supplementary Fig. 7 ^{13}C NMR spectrum of **3**.

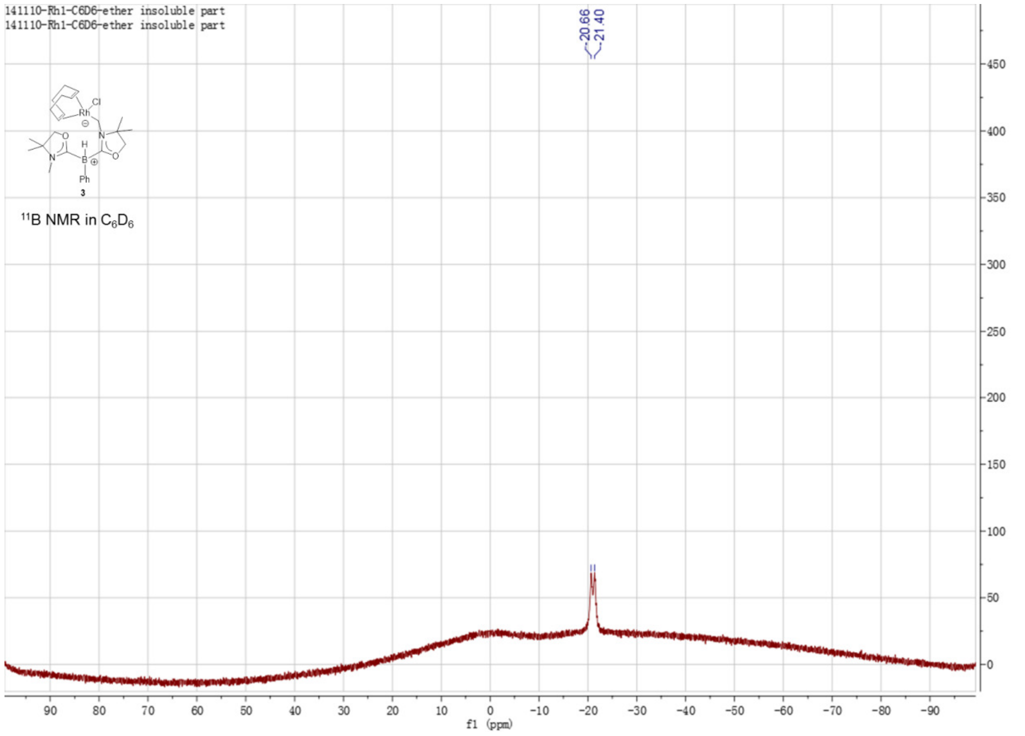

Supplementary Fig. 8 ^{11}B NMR spectrum of **3**.

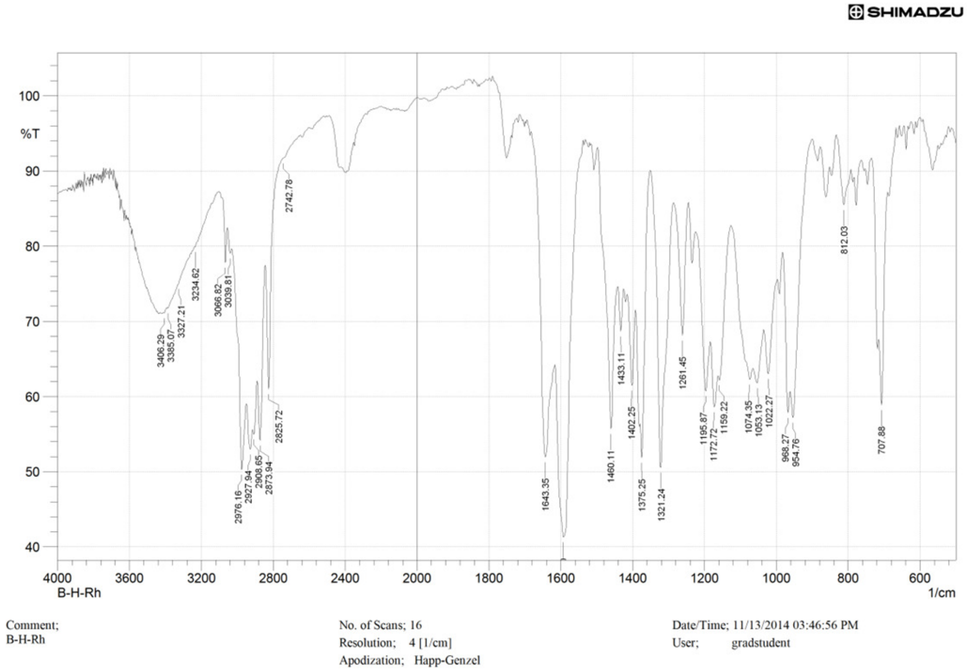

Supplementary Fig. 9 IR spectrum of **3**.

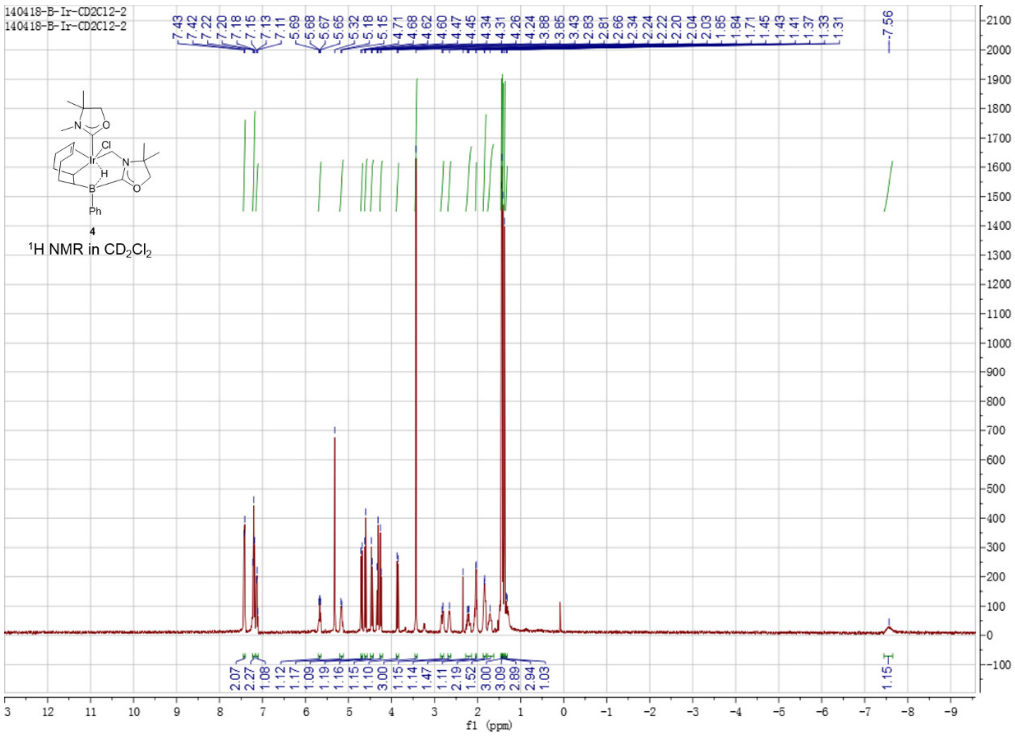

Supplementary Fig. 10 ^{1}H NMR spectrum of **4**.

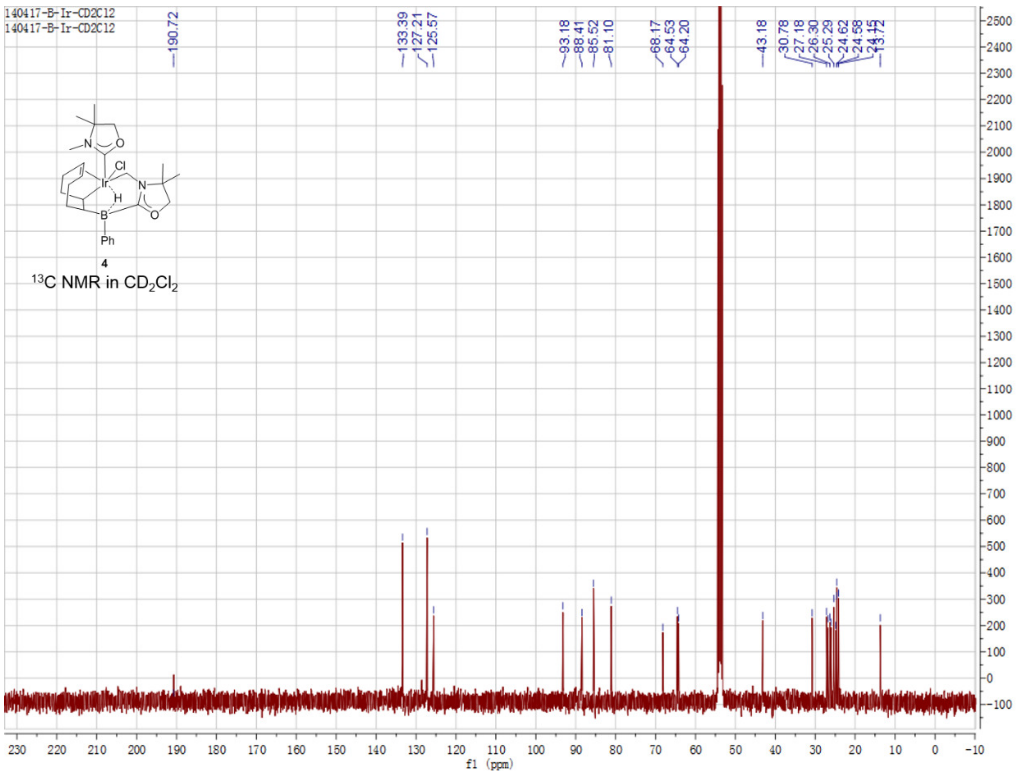

Supplementary Fig. 11 ^{13}C NMR spectrum of **4**.

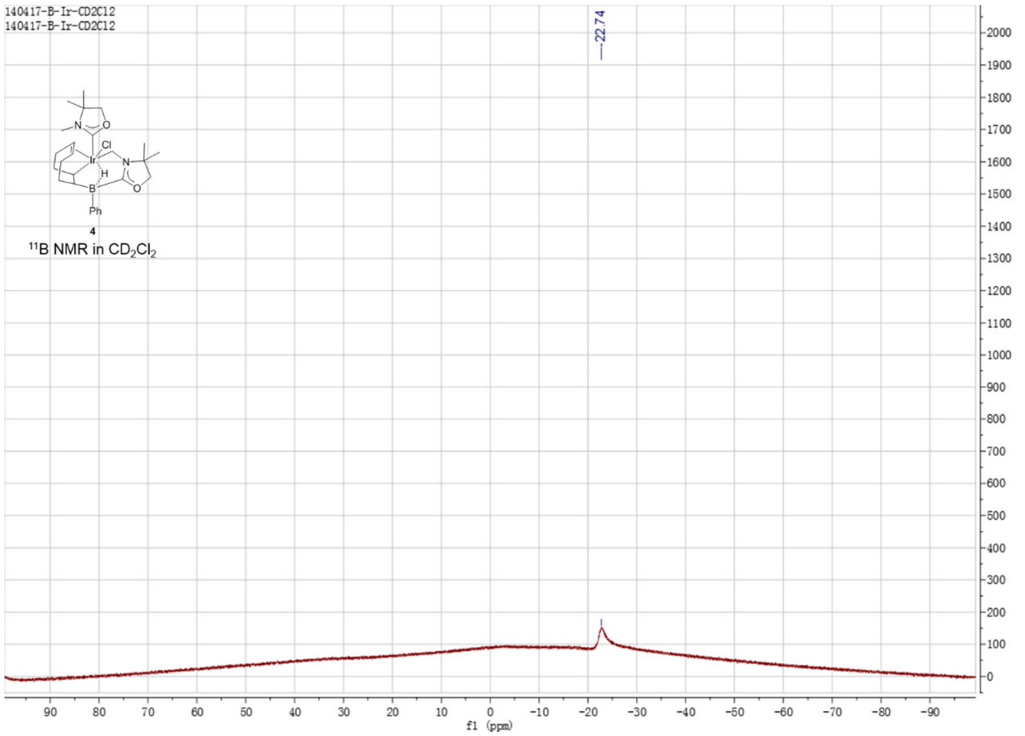

Supplementary Fig. 12 ^{11}B NMR spectrum of **4**.

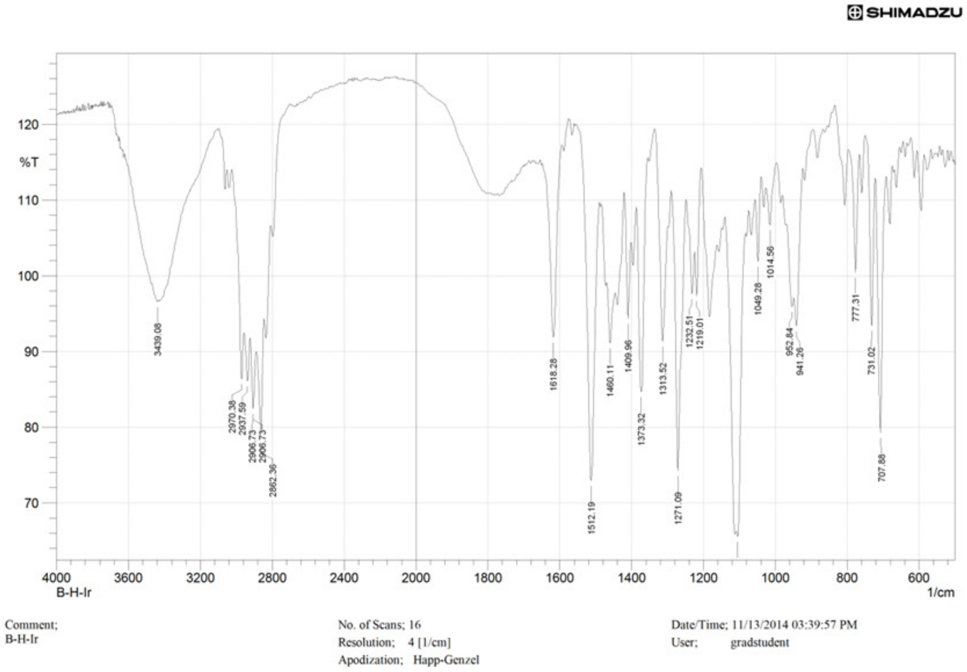

Supplementary Fig. 13 IR spectrum of compound **4**.

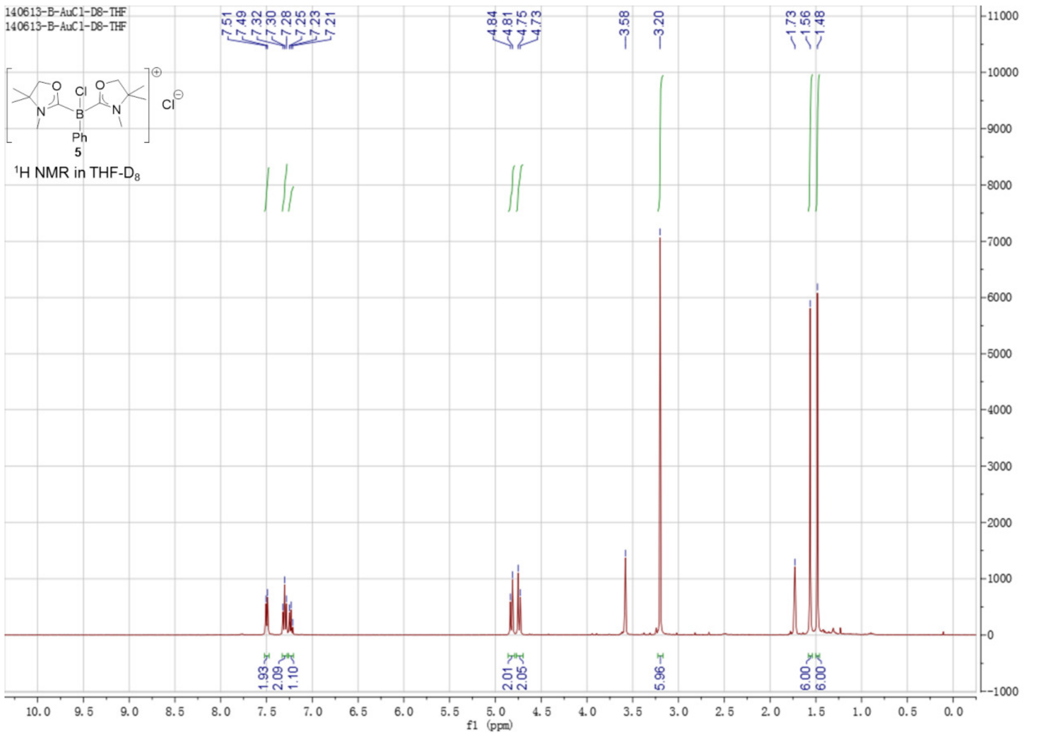

Supplementary Fig. 14 ^{1}H NMR spectrum of **5**.

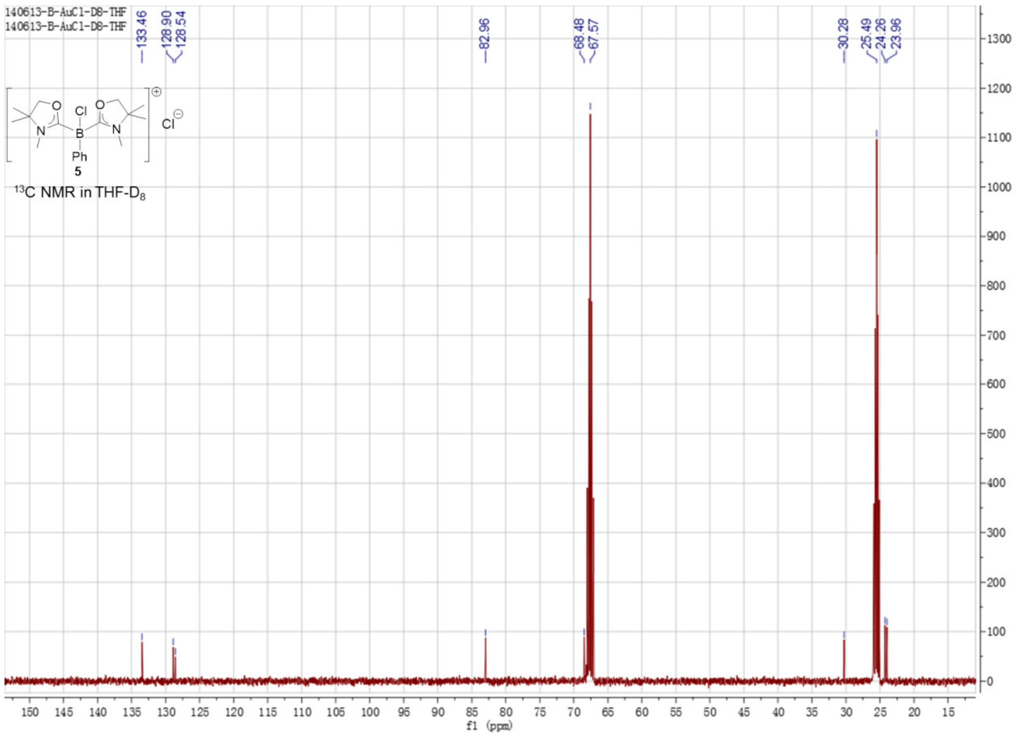

Supplementary Fig. 15 ^{13}C NMR spectrum of **5**.

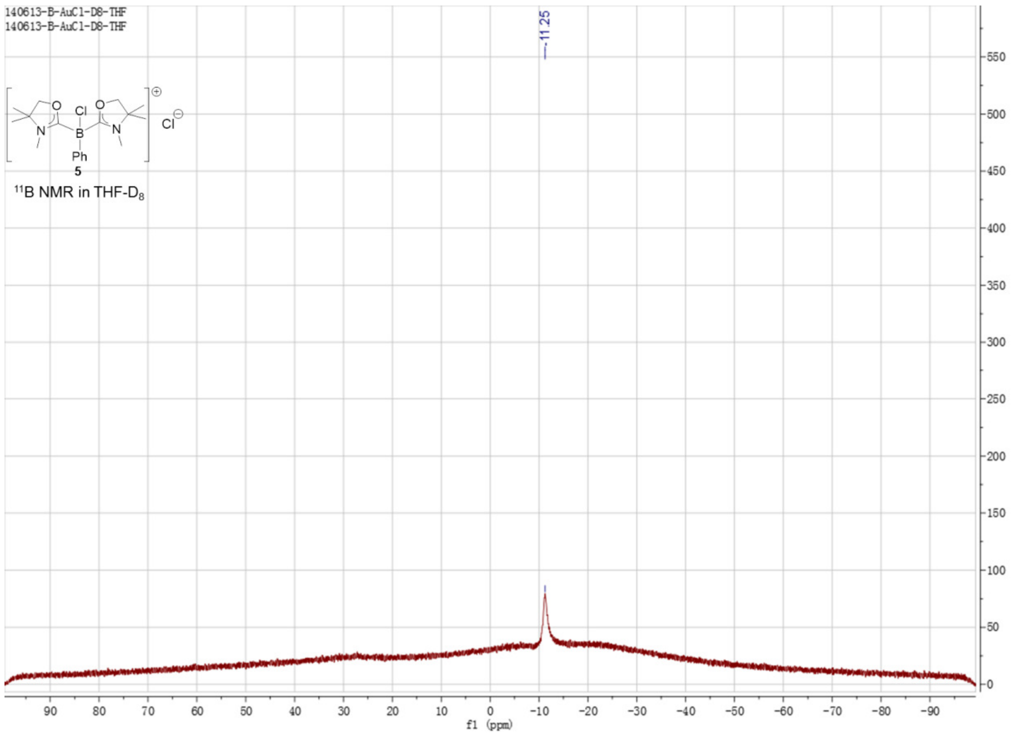

Supplementary Fig. 16 ^{11}B NMR spectrum of **5**.

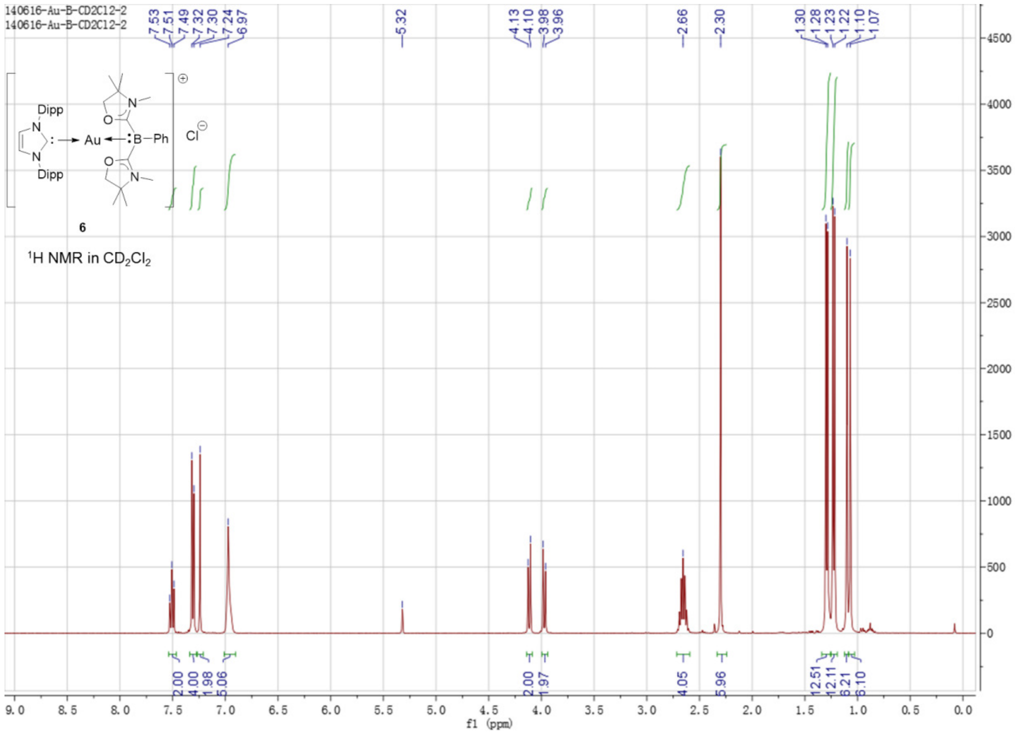

Supplementary Fig. 17 ^{1}H NMR spectrum of **6**.

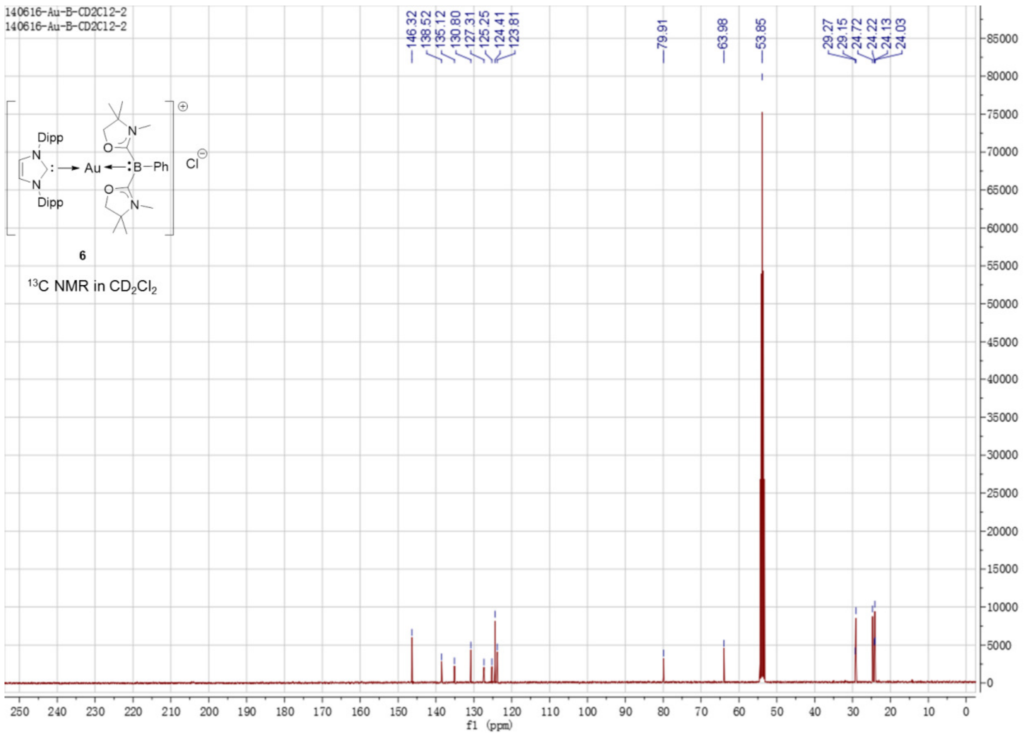

Supplementary Fig. 18 ^{13}C NMR spectrum of **6**.

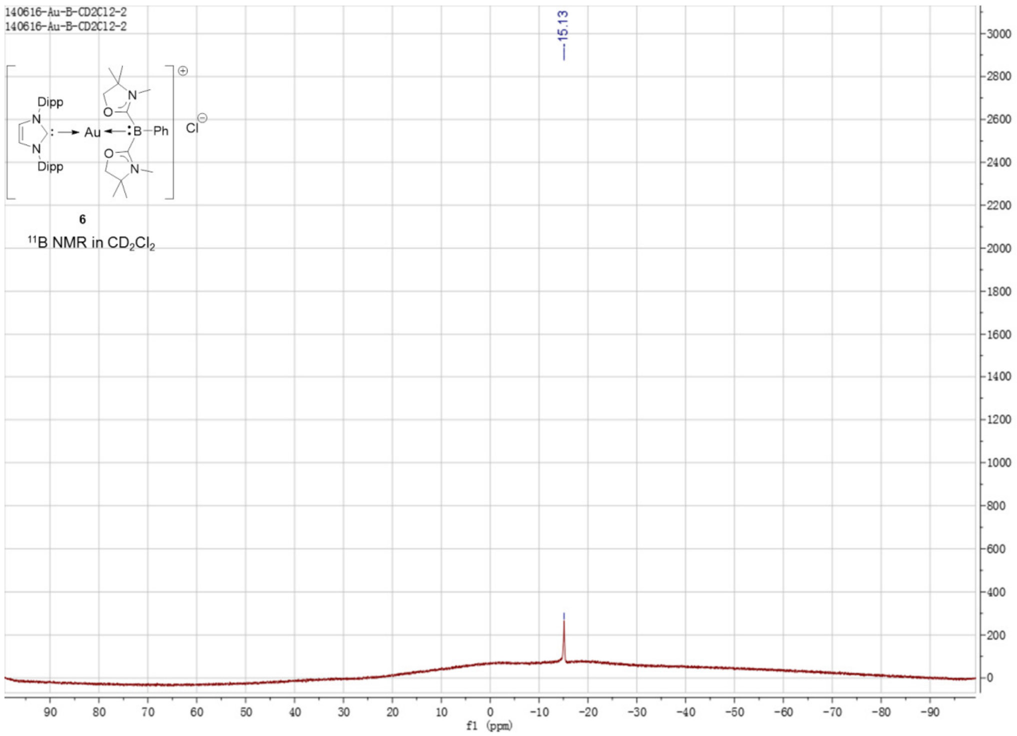

Supplementary Fig. 19 ^{11}B NMR spectrum of **6**.

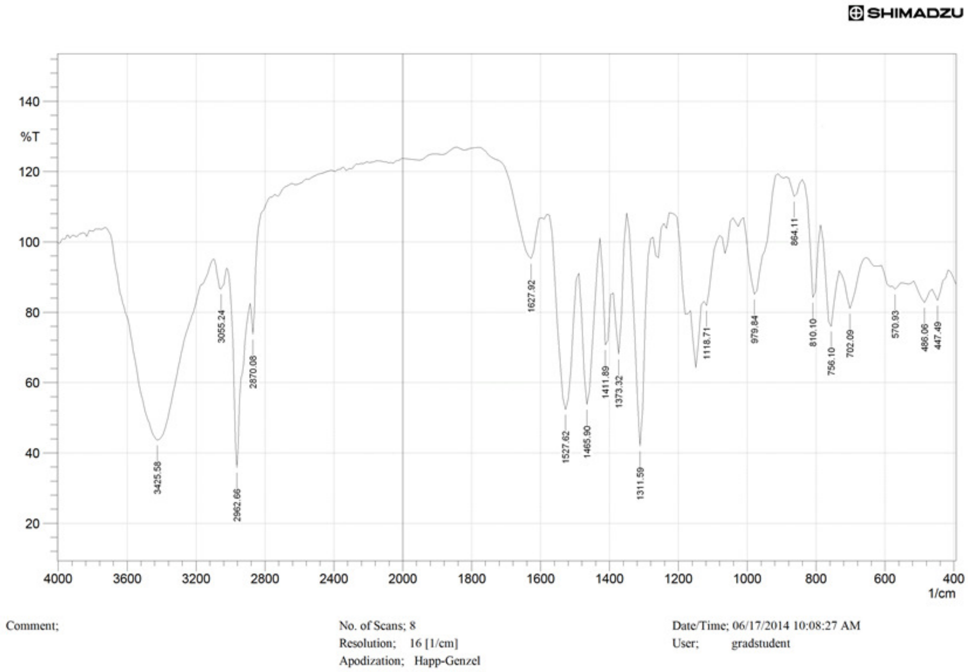

Supplementary Fig. 20 IR spectrum of compound **6**.

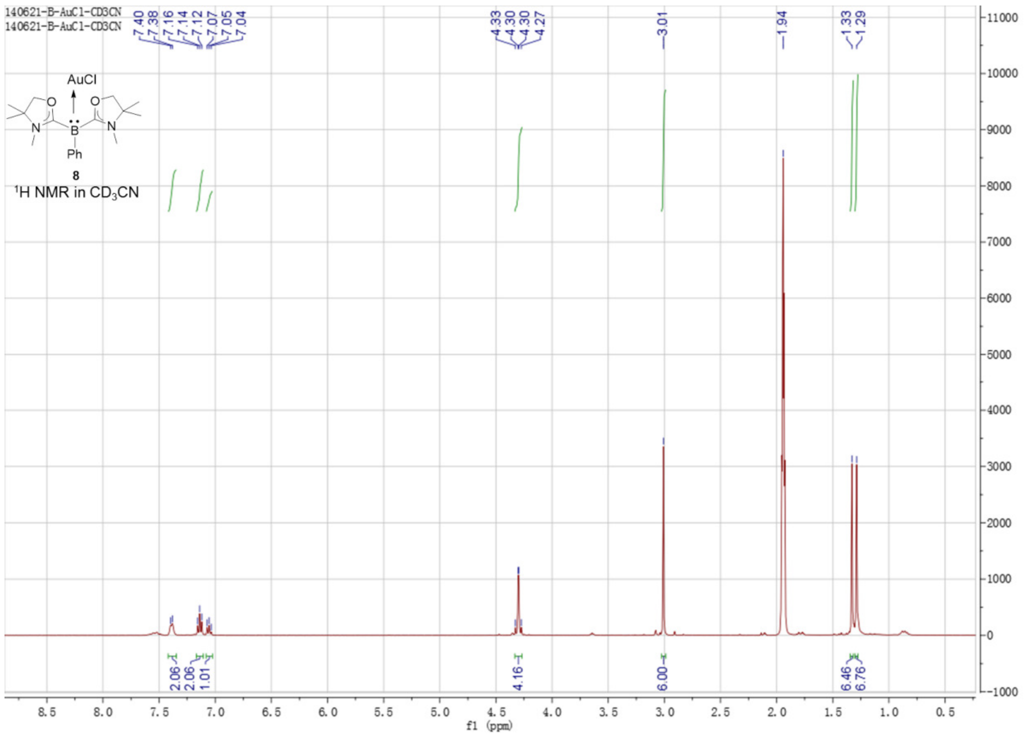

Supplementary Fig. 21 ^{1}H NMR spectrum of **8**.

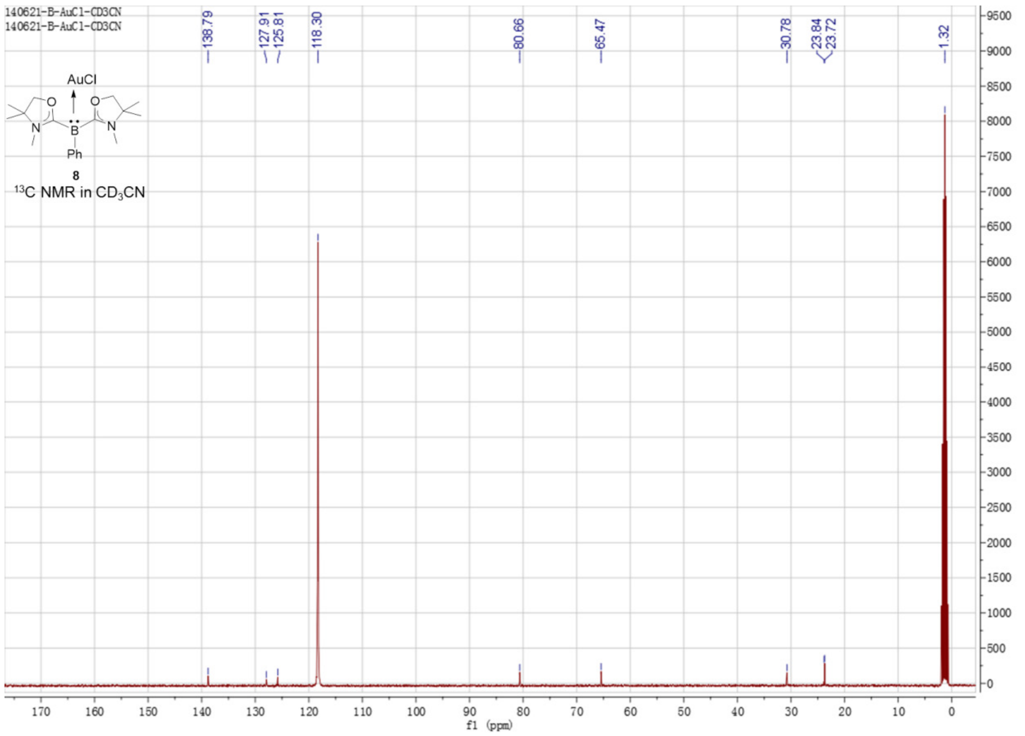

Supplementary Fig. 22 ^{13}C NMR spectrum of **8**.

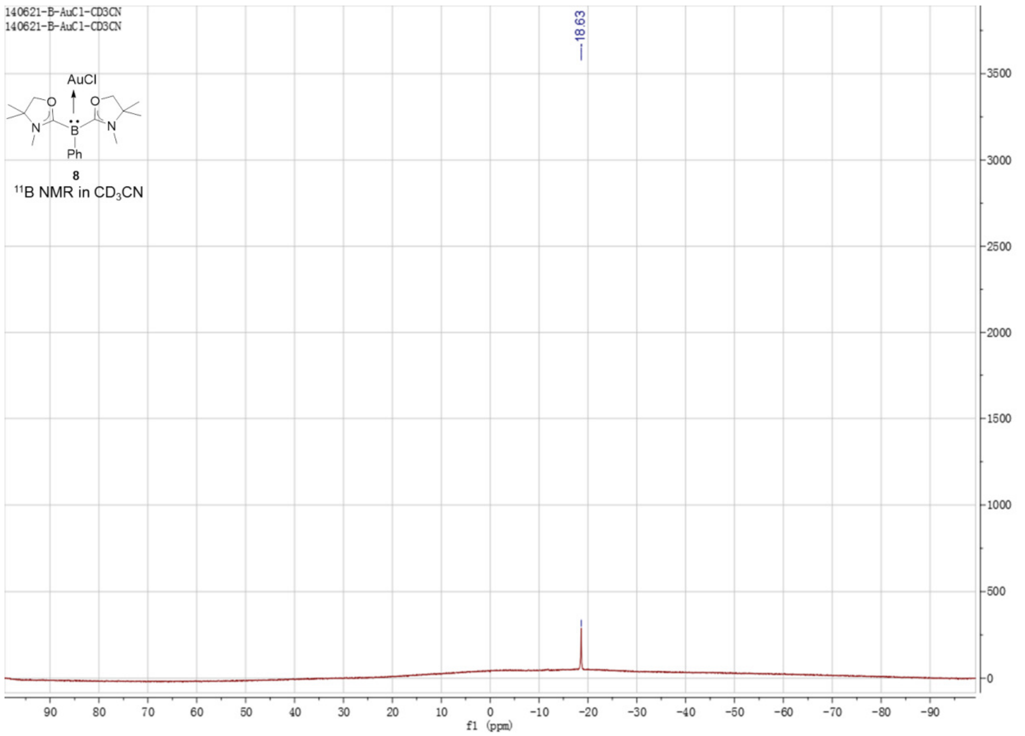

Supplementary Fig. 23 ^{11}B NMR spectrum of **8**.

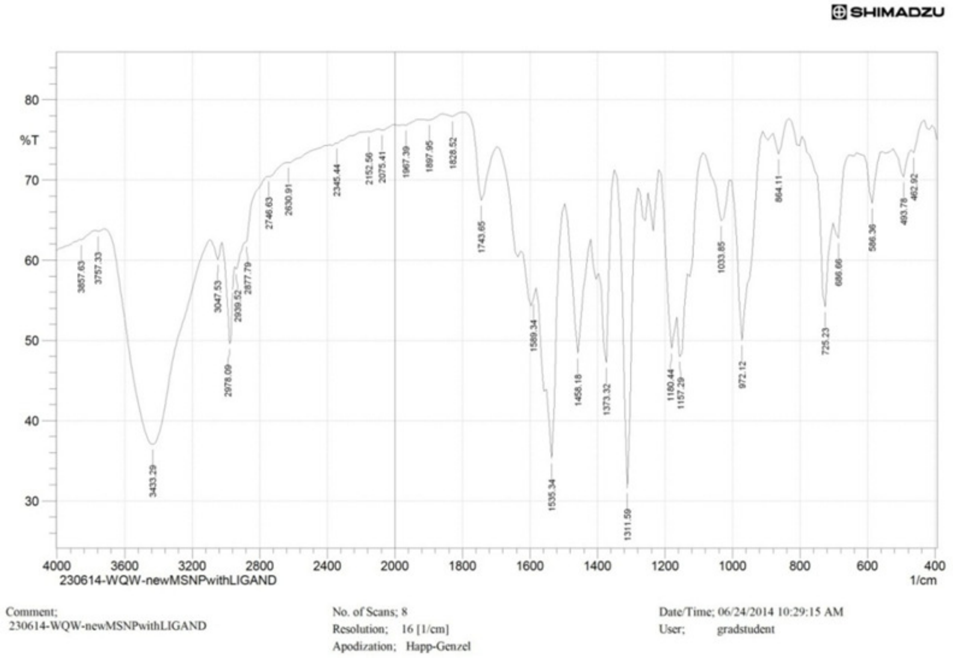

Supplementary Fig. 24 IR spectrum of compound **8**.

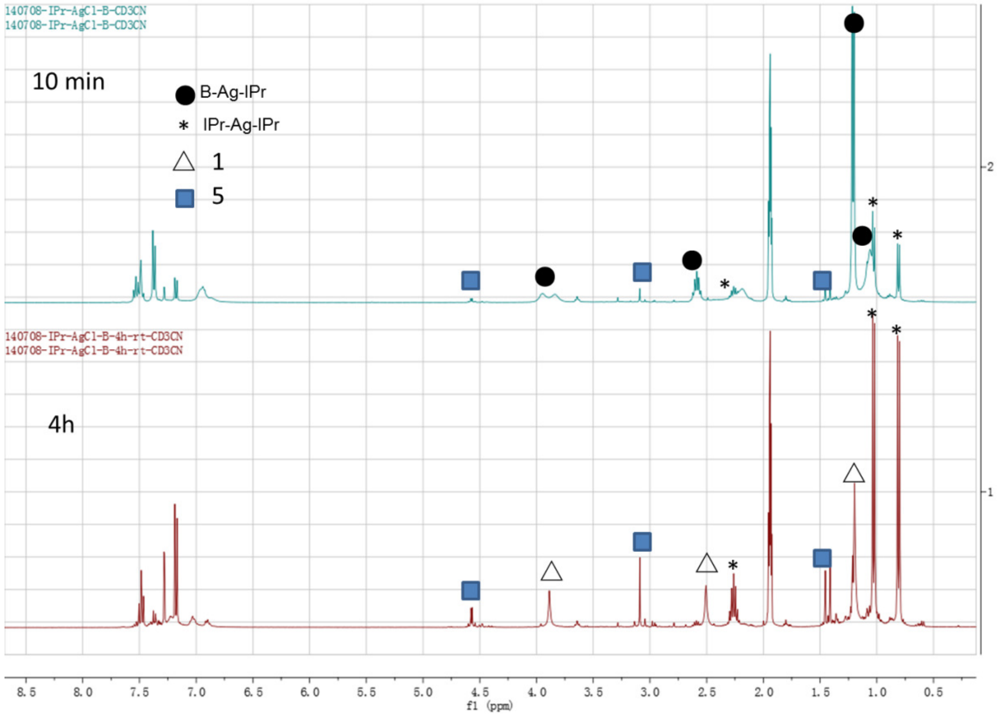

Supplementary Fig. 25 Reaction of **1** with (IPr)AgCl monitored by ^{1}H NMR (CD_3CN).

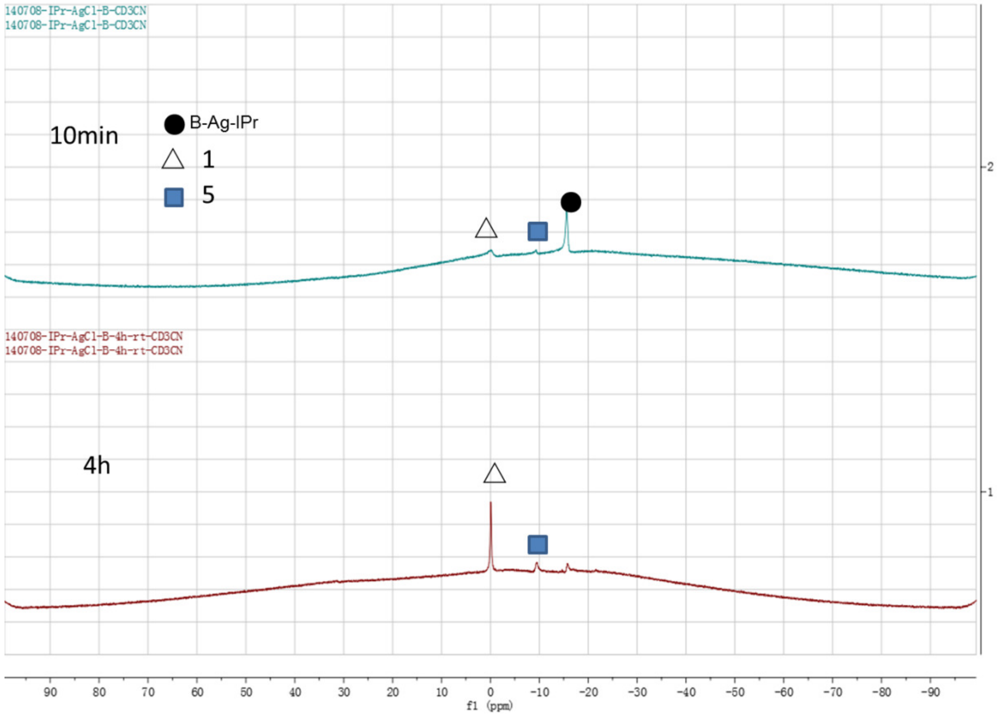

Supplementary Fig. 26 Reaction of **1** with (IPr)AgCl monitored by ^{11}B NMR (CD_3CN).

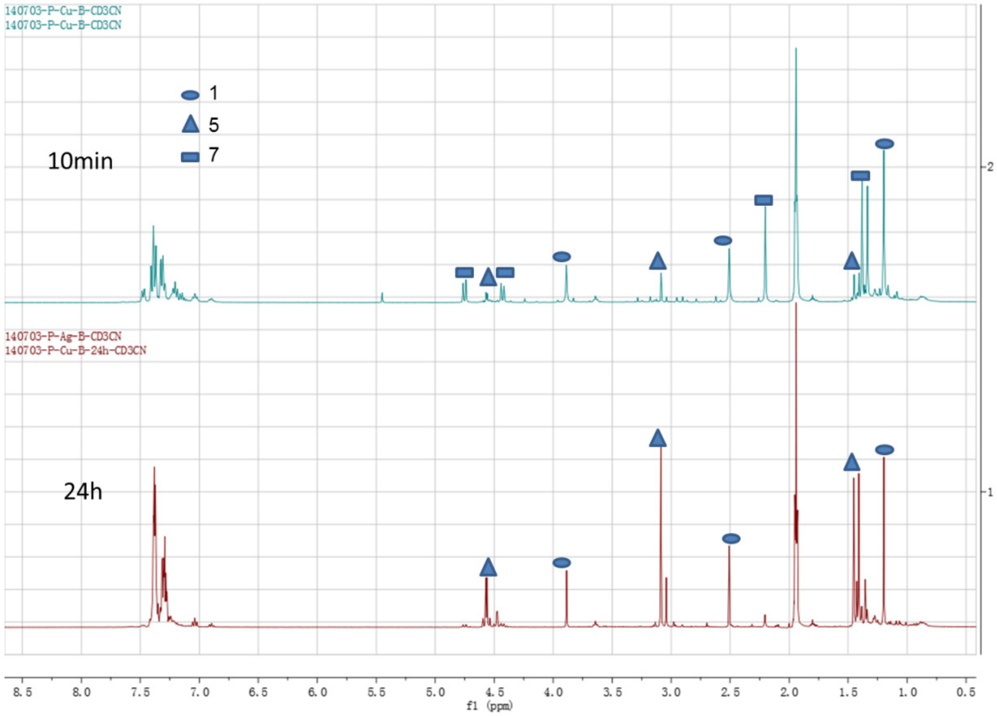

Supplementary Fig. 27 Reaction of **1** with $(Ph_3P)CuCl$ monitored by ^1H NMR (CD_3CN).

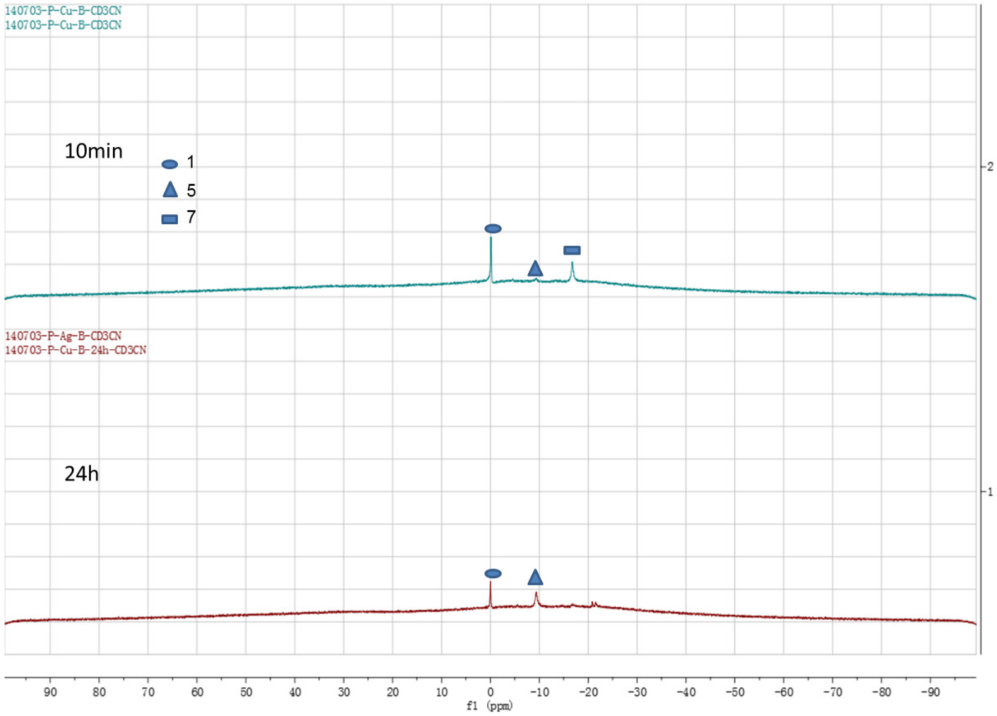

Supplementary Fig. 28 Reaction of **1** with (Ph3P)CuCl monitored by ^{11}B NMR (CD_3CN).

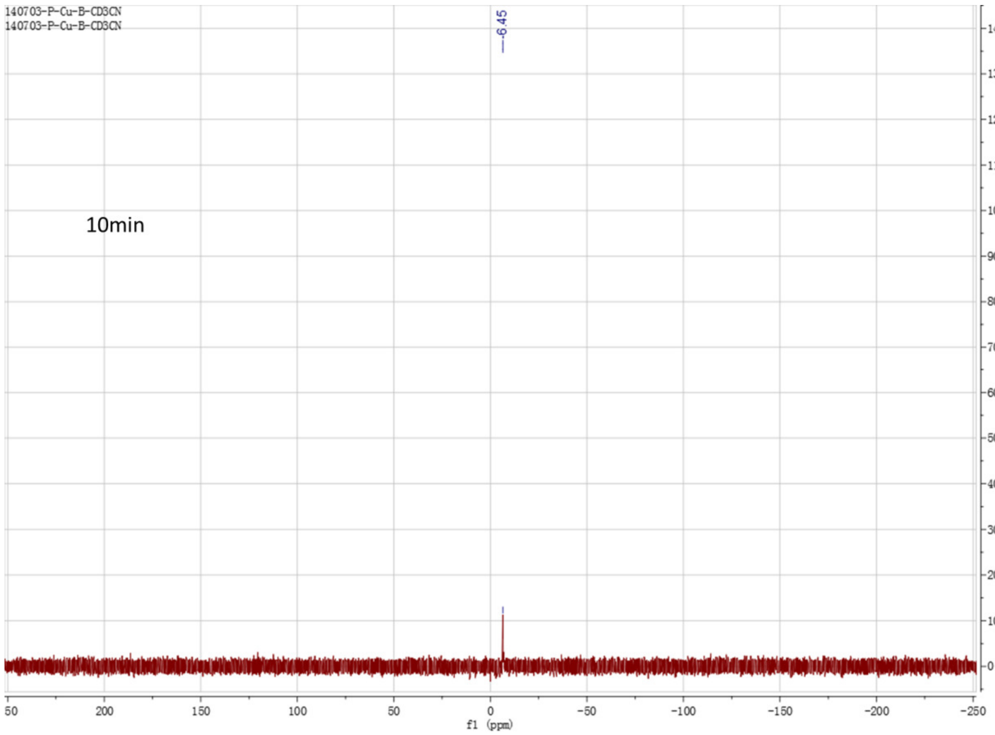

Supplementary Fig. 29 Reaction of **1** with $(Ph_3P)CuCl$ monitored by ^{31}P NMR (CD_3CN).

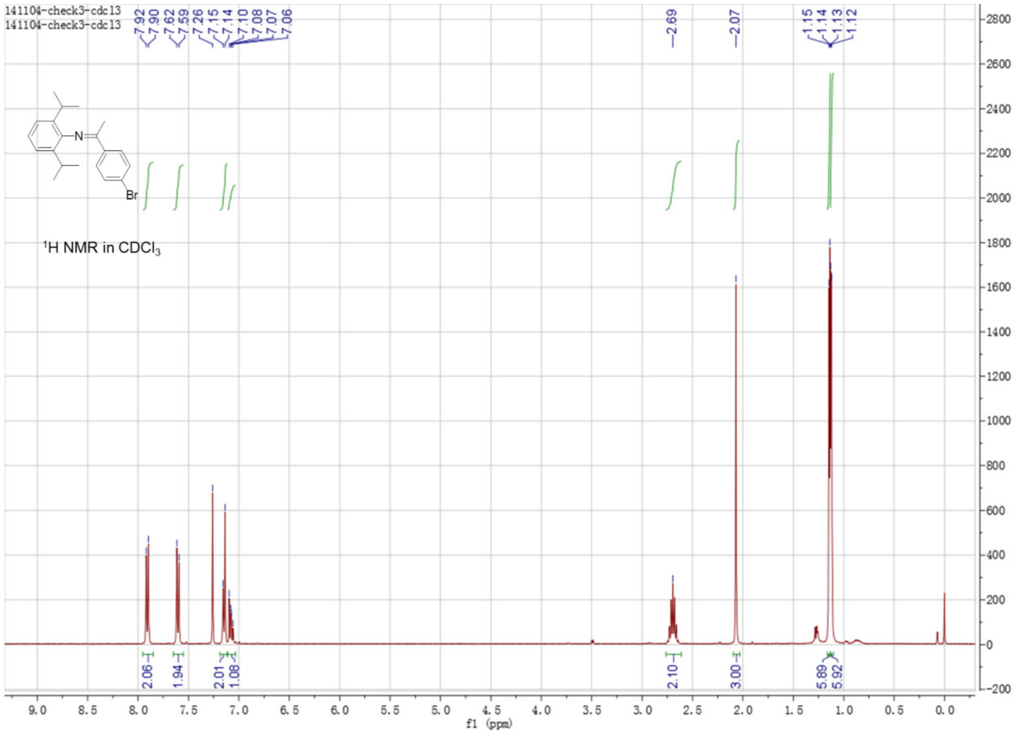

Supplementary Fig. 30 ^1H NMR spectrum of **11ec**.

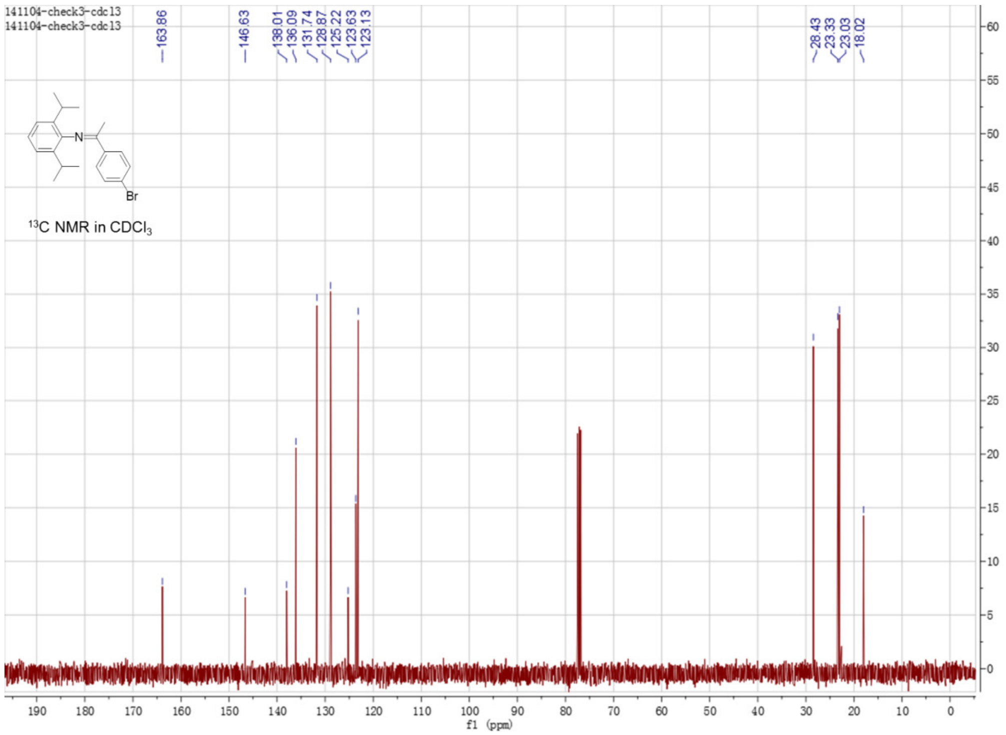

Supplementary Fig. 31 ^{13}C NMR spectrum of **11ec**.

Control experiments:

To investigate the stability of compound **8** during the catalysis reaction, the catalytic reaction between phenylacetylene and aniline with 20 mol% of **8** was monitored by the ^{11}B NMR spectroscopy. A major signal was observed at –16.6 ppm, in addition to the peak at –16.8 ppm for $B(C_6F_5)_4$ and a minor peak at –19.3 ppm (Supplementary Fig. 32d). The major peak corresponds to neither free L_2PhB: **1** (^{11}B NMR: δ = –1.1) nor **5** (^{11}B NMR: δ = –11.2), potential decomposed products from **8**, supporting no decomposition of the B-Au bond. These signals remained until the catalytic reaction completed (Supplementary Fig. 32e). Meanwhile, in a stoichiometric reaction of **8** with aniline ($PhNH_2$), the aniline complex $[(L_2PhB)Au(NH_2Ph)]B(C_6F_5)_4$ **S1** was formed, and the ^{11}B NMR peak appeared at –20.1 ppm (Supplementary Fig. 32b), indicating that this species is not the resting state of the catalyst. The reaction of **8** and alkyne (HC≡CPh) in the presence of NEt_3 afforded a ^{11}B NMR chemical shift at –16.6 ppm, in addition to a peak at –20.2 ppm for $[(L_2PhB)Au(NEt_3)]B(C_6F_5)_4$ **S2**, which presumably corresponds to either $(L_2PhB)Au(C{\equiv}CPh)$ or σ,π-acetylide complex $[(L_2PhB)Au]_2(C{\equiv}CPh){\cdot}B(C_6F_5)_4$, proposing the resting state of the catalyst **S3** (Supplementary Fig. 32c).[16] Although further analysis is required, a peak at –19.3 ppm was tentatively assigned to phenylethenyl-gold complex **S4** (Supplementary Fig. 32d-e).

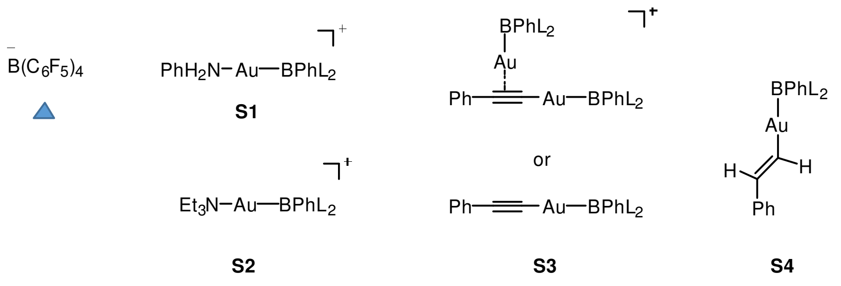

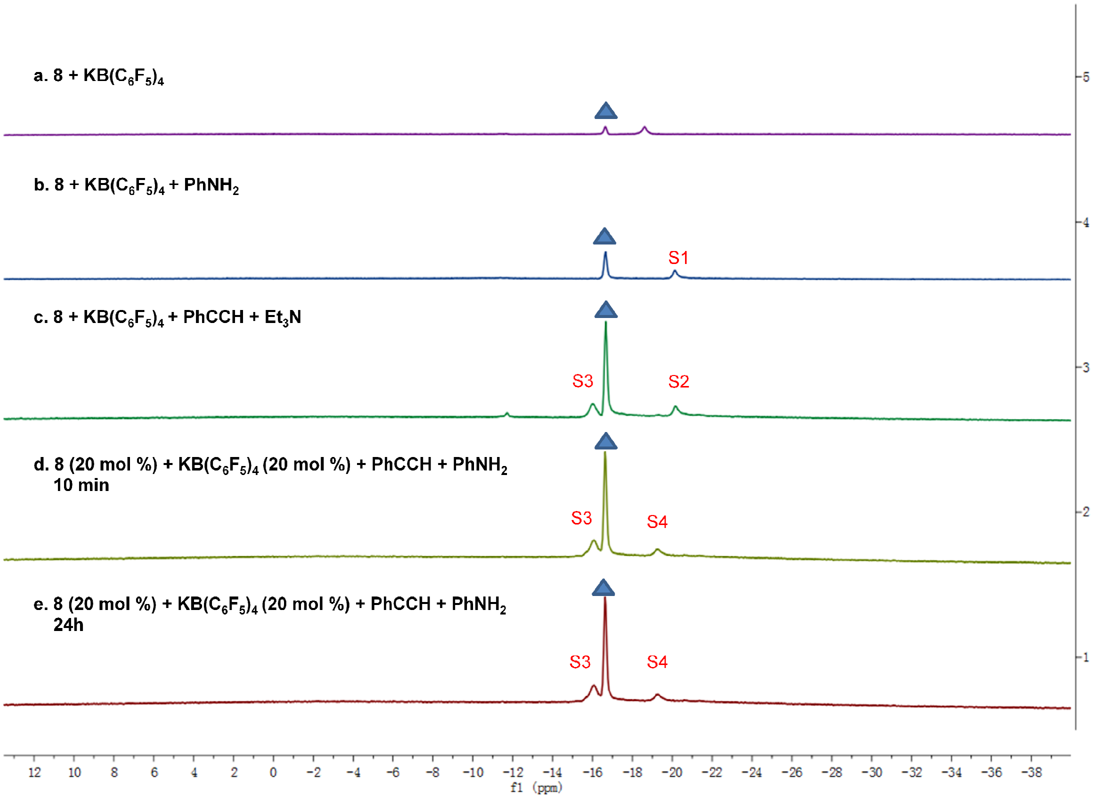

Supplementary Fig. 32. Comparison of the ^{11}B NMR spectra of the control experiments.

2

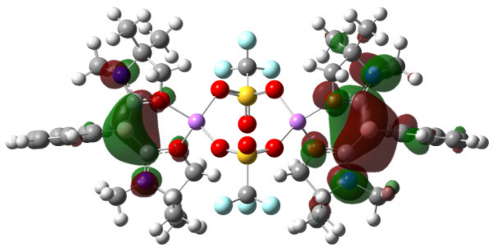

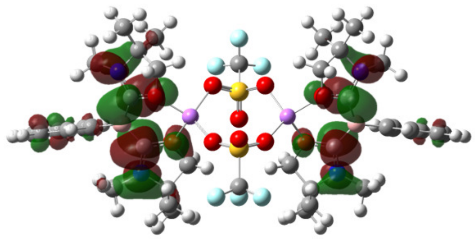

HOMO (–5.355) **LUMO (0.0346)**

3

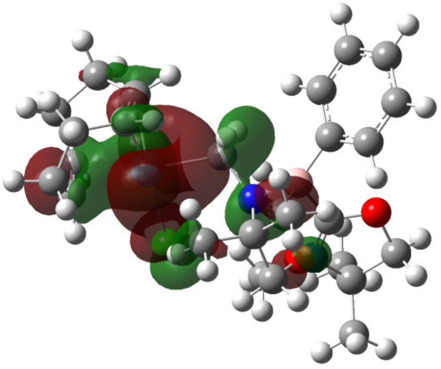

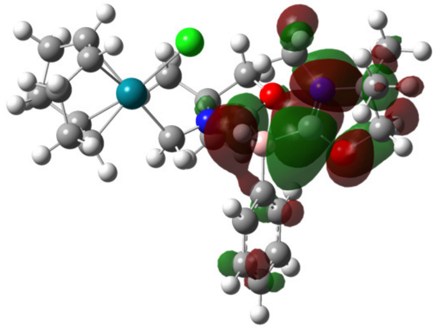

HOMO (–4.804) **LUMO (0.338)**

4

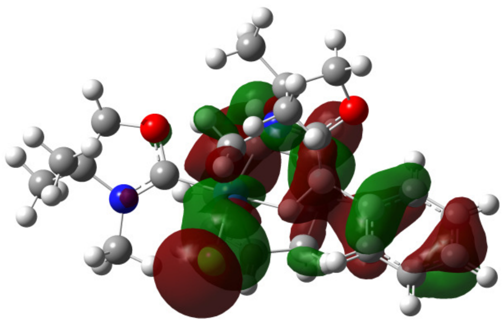

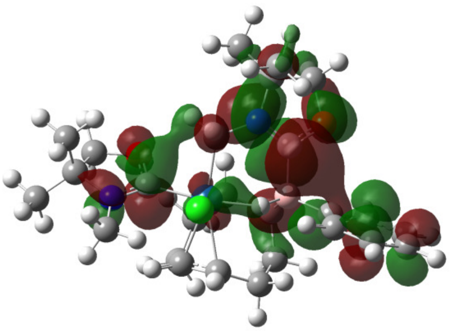

HOMO (–7.043) **LUMO (0.839)**

6

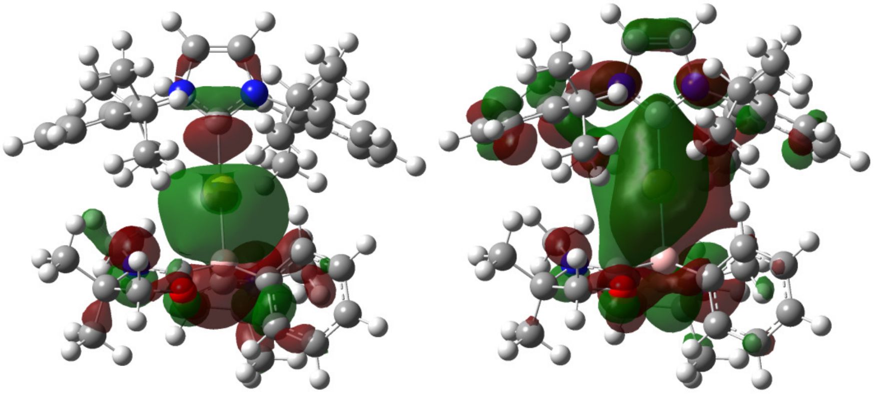

HOMO (–9.138) **LUMO (–2.413)**

8

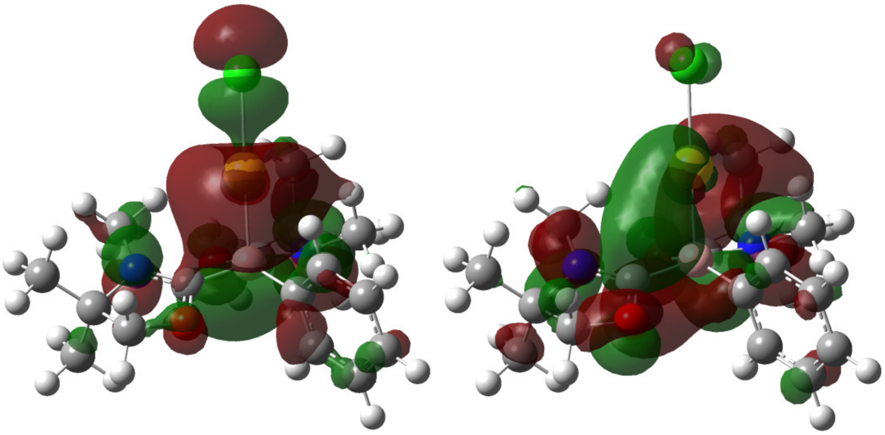

HOMO (–6.779) **LUMO (-0.017)**

Supplementary Fig. 33 Plots of the frontier orbitals of compounds **2**, **3**, **4, 6** and **8**.

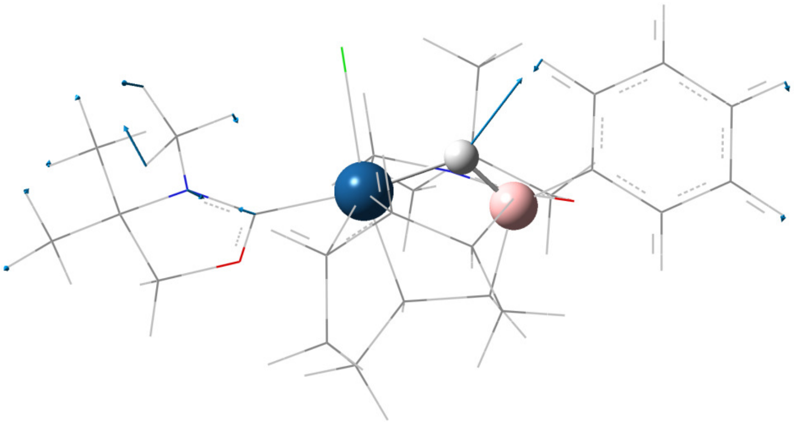

4: # 167(1589.55 cm^{-1})

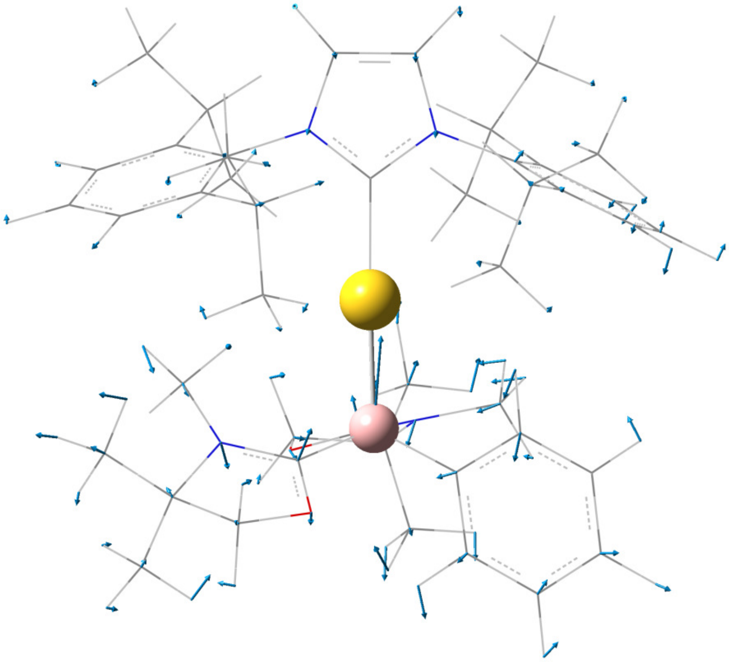

6: # 89(581.91 cm^{-1})

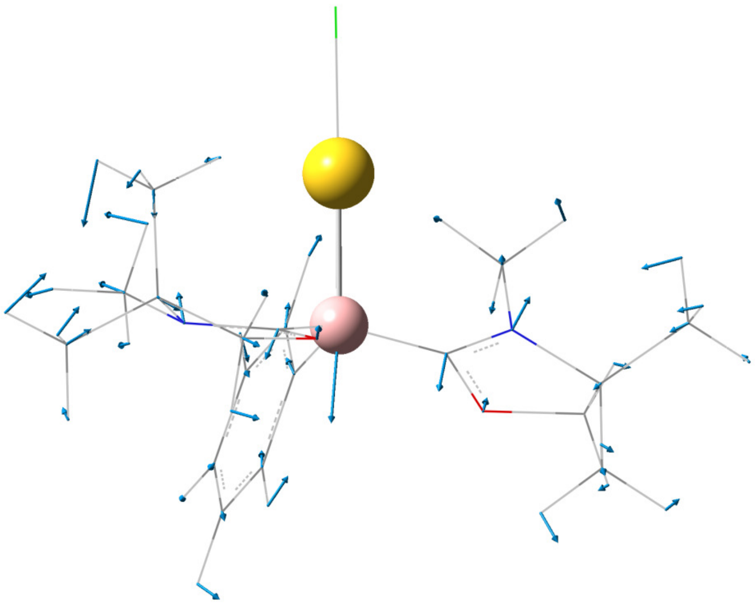

8: # 41 (577.36 cm^{-1})

Supplementary Fig. 34 Frequency calculation results of **4**, **6** and **8**. The picture displays that the vibration mode involves Ir-H-B bending vibration in **4**, and Au-B stretching in **6** and **8**.

Supplementary Note 1 Coordinates for optimized geometries.

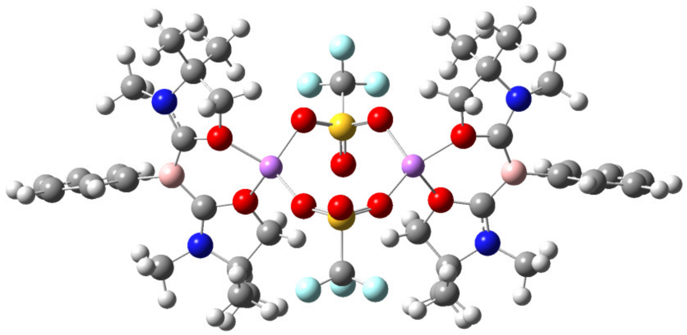

2

2

B	-5.52225024	-0.04103128	-0.30921150
C	-5.09765552	1.20025678	0.40510858
C	-4.76355165	3.43201014	0.98892156
C	-3.88239415	2.53140315	1.83547025
C	-6.72888915	2.80908846	-0.46559122
C	-5.44154230	4.50284205	1.82920913
C	-3.96518572	4.03129072	-0.17125257
C	-4.49173203	-1.10935917	-0.51617520
C	-3.37796574	-3.13424275	-0.86694394
C	-2.39897594	-1.97288133	-0.88921511

C	-6.99755254	-0.20832402	-0.88365407
C	-7.25075555	-0.22700502	-2.26082817
C	-8.53680766	-0.38062203	-2.76714421
C	-9.61493873	-0.51550004	-1.89950715
C	-9.39269672	-0.49672904	-0.52747204
C	-8.10088960	-0.34783003	-0.03341900
C	-0.00053100	2.78061921	-1.74531913
F	-1.08343008	2.66898120	-2.50876719
F	-0.00077500	3.98203230	-1.17302809
F	1.08154108	2.66994720	-2.51001819

C	-5.91086140	-3.06935305	-0.95406642
C	-3.15234593	-4.08024275	-2.03626118
C	-3.32679876	-3.86263078	0.47748930
O	-3.93145330	1.27475410	1.13629209
O	-3.15278024	-0.90810307	-0.27863402
O	-1.23440410	1.76260813	0.29750802
O	1.23710110	1.76381313	0.29565302
O	0.00097100	0.20035402	-1.16934709
S	0.00090700	1.47273711	-0.45632603
F	-1.08120708	-3.48685626	3.28710225
O	-1.23130610	-0.88837607	1.91305115
Li	2.29391417	0.30100002	0.92297307
C	0.00059200	-3.01508023	2.67993520
C	3.96465830	4.03128031	-0.16748701
S	-0.00081700	-1.17651009	2.67579821
O	3.15294524	-0.90899407	-0.27723602
O	3.93215530	1.27335510	1.13849308
O	1.23057010	-0.88615507	1.91534015
F	1.08263708	-3.48534927	3.28784625
F	0.00134200	-3.46873827	1.41398611
C	4.76374337	3.43097926	0.99166907
O	-0.00240800	-0.77075506	4.06417331
C	3.88313330	2.52974019	1.83817114
C	4.49181134	-1.10973408	-0.51578204
C	2.39904518	-1.97345315	-0.88821207
C	5.09768339	1.19959809	0.40623903
C	5.44231543	4.50115634	1.83235714
N	5.71591742	2.41169019	0.50246604
B	5.52213642	-0.04114800	-0.30909902
N	4.63534535	-2.36981418	-1.00237207
C	6.72779952	2.80927421	-0.46498804
C	6.99709756	-0.20786002	-0.88456107
C	8.10099760	-0.34713303	-0.03501900
C	7.24950458	-0.22618402	-2.26188917
C	8.53531665	-0.37925003	-2.76898321
H	6.41567750	-0.11760201	-2.94549822
C	9.61400674	-0.51392604	-1.90201514
H	8.69791467	-0.38923703	-3.83894329
C	9.39256274	-0.49548604	-0.52984504
H	10.61622379	-0.63098805	-2.29176317
H	-2.84536622	2.85212922	1.88027914
H	-4.29834933	2.38389318	2.83336921
H	-6.37834050	2.65288820	-1.48859611

Li	-2.29388117	0.30142602	0.92214407
N	-5.71607543	2.41226318	0.50122304
N	-4.63526735	-2.36973218	-1.00215608
H	-4.68500636	5.10381539	2.33580718
H	-6.08910449	4.04350831	2.57516320
H	-3.42964226	3.24596325	-0.70612306
H	-3.23444125	4.74657736	0.20866602
H	-4.62472135	4.55117435	-0.86636006
H	-1.50324411	-2.12885016	-0.29801502
H	-2.14352616	-1.66726413	-1.90415714
H	-6.37475950	-2.96350222	0.02852300
H	-5.72947145	-4.12164732	-1.15853609
H	-6.59545250	-2.68224420	-1.70268013
H	-3.83769229	-4.92683838	-2.00145915
H	-2.13722816	-4.47760034	-1.99333615
H	-3.28807525	-3.55341627	-2.97996423
H	-3.39931126	-3.14587224	1.29655610
H	-2.38394218	-4.40088133	0.57416005
H	-4.14285932	-4.58046835	0.56272204
H	-6.41735546	-0.11831601	-2.94493523
H	-8.70002069	-0.39088803	-3.83700429
H	-10.61734178	-0.63299505	-2.28864218
H	-10.22472777	-0.60218204	0.15651901
H	-7.93715358	-0.34020903	1.03763708
H	3.42869826	3.24643525	-0.70264105
H	2.84605722	2.85026022	1.88349614
H	3.23422725	4.74633036	0.21347902
H	4.62382135	4.55166635	-0.86256306
H	4.29945033	2.38189518	2.83586222
C	3.37828026	-3.13466824	-0.86724007
H	1.50359511	-2.12998816	-0.29671002
H	2.14302317	-1.66718113	-1.90280115
H	6.03506448	5.17541140	1.21479309
H	4.68612536	5.10160839	2.34009018
H	6.09053348	4.04123431	2.57738020
C	5.91122045	-3.06902823	-0.95620307
H	6.37643847	2.65341220	-1.48777111
H	6.95154154	3.86203430	-0.31098902
H	7.63835460	2.23649217	-0.32165802
H	7.93787760	-0.33974603	1.03613708
H	10.22503078	-0.60076605	0.15364501
C	3.15238324	-4.07983131	-2.03717216
C	3.32788925	-3.86404629	0.47669604

H	-6.95285453	3.86182829	-0.31184002
H	-7.63911461	2.23604417	-0.32124002
H	-6.03486948	5.17650640	1.21155609
H	6.37695151	-2.96218722	0.02538500
H	5.72972143	-4.12156031	-1.15933009
H	6.59429551	-2.68240521	-1.70647313

H	3.40065626	-3.14780524	1.29619410
H	2.38522718	-4.40260934	0.57344304
H	4.14418531	-4.58171135	0.56108704
H	3.83818229	-4.92610938	-2.00349215
H	2.13748816	-4.47772934	-1.99394915
H	3.28726625	-3.55218527	-2.98054123

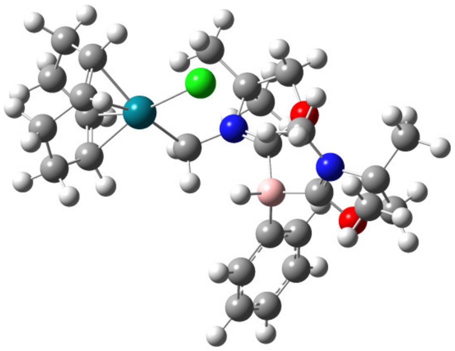

3

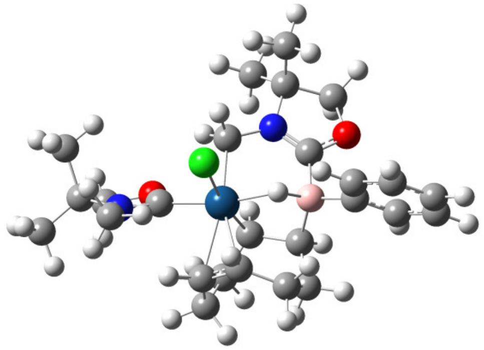

4

3

C	-3.43403198	1.35297049	-1.07981298
H	-2.73520843	2.18058999	-1.15194697
C	-3.98965670	1.09450497	0.18474850
H	-3.66708402	1.73488708	0.99792749
C	-5.35547823	0.45119148	0.38712886
H	-6.01096623	0.72057567	-0.44068803
H	-5.81555139	0.87109756	1.28236082
C	-5.27258851	-1.07766892	0.54687175
H	-5.05984068	-1.30312713	1.59312099
H	-6.24011601	-1.53804206	0.31788120
C	-4.17370521	-1.71630591	-0.27273958
H	-3.72149932	-2.60321417	0.15393084
C	-3.87263032	-1.42316472	-1.57701085
H	-3.18656336	-2.08456167	-2.09216648
C	-4.66219600	-0.44493933	-2.42317990
H	-5.69741352	-0.42971381	-2.08620382
H	-4.68258414	-0.80480963	-3.45238516
C	-4.06180110	0.97373843	-2.40475019
H	-3.27342555	1.02671421	-3.15751934
H	-4.82234132	1.70822296	-2.69485293
C	-0.99353637	1.06111915	0.44294571
H	-0.48308904	1.48273106	-0.42191037

H	0.51233017	-1.92421723	2.65907118
C	1.21328343	0.09530163	0.89802663
C	2.19829465	2.07403244	-0.53676604
C	1.86694909	2.81890100	-1.67008830
H	1.43099603	2.31207834	-2.52242946
C	2.07361822	4.19383491	-1.72330283
H	1.80511654	4.74644929	-2.61422898
C	2.61760821	4.85801232	-0.63200898
H	2.77628353	5.92747058	-0.66773381
C	2.95392018	4.13837768	0.50930495
H	3.37764265	4.64766756	1.36503035
C	2.74488296	2.76587991	0.54893718
H	3.01906434	2.21606324	1.44375240
C	3.34486439	-0.32950412	-0.51427561
C	5.57792032	-0.64781940	-0.35194442
H	5.91600442	-0.63902746	0.68334127
H	6.37720218	-0.33255488	-1.01543648
C	4.94710392	-1.98799396	-0.74701430
C	5.25851094	-2.35595436	-2.19530063
H	4.70057625	-3.23879727	-2.50425747
H	6.32189803	-2.57539287	-2.29561429
H	5.00545028	-1.52944270	-2.85962595

H	-1.42219750	1.88963574	1.01358106
B	1.94744094	0.48389293	-0.47860781
H	1.27935222	0.08088521	-1.40119935
C	-0.18237272	0.13234551	2.71455139
C	-1.57175846	-0.40390460	3.01033738
H	-2.32530343	0.36227153	2.83240371
H	-1.61431514	-0.69599733	4.06186990
H	-1.79329156	-1.26330894	2.38039084
C	0.12492264	1.38868969	3.52878089
H	1.15100385	1.71438832	3.35411082
H	-0.00955844	1.18320842	4.59140130
H	-0.54674341	2.19733603	3.24348868
C	0.89973010	-0.93024993	2.88015795
H	1.40347185	-0.89339641	3.84154995

C	5.31315723	-3.10765952	0.21560119
H	4.99097978	-2.86054655	1.22698009
H	6.39396562	-3.25149714	0.21443155
H	4.84815788	-4.04595489	-0.08486622
C	2.45012608	-2.60649807	-0.67140118
H	1.48980077	-2.10966605	-0.77078919
H	2.44441599	-3.18619031	0.24989157
H	2.60676283	-3.26617729	-1.52289816
N	0.03647629	0.46074892	1.28011536
N	3.51812910	-1.61842754	-0.62255762
O	1.86677796	-0.60325317	1.84871426
O	4.50040891	0.31148490	-0.46042876
Cl	-0.92846207	-2.22729036	-0.05487290
Rh	-2.45793967	-0.28328199	-0.23421896

4

Ir	-0.48521376	-0.35130862	0.18168652
C	-2.39184172	0.02344401	-0.08048964
C	-0.01656343	-0.34545668	-1.81955163
C	-0.25902472	1.70405582	0.36389735
C	-0.03731123	-2.61521652	0.21203210
C	-1.23822050	-2.44979551	-0.43750452
Cl	-0.58324610	-0.41525275	2.75025142
B	2.03775759	-0.15132870	-0.41078136
H	1.24389558	-0.42318569	0.59640021
C	2.01751080	1.44303664	-0.48797176
C	3.45070506	-0.64889560	0.16727491
C	1.46044038	-0.79189584	-1.79684204
N	1.01934065	2.20508330	-0.16502419
H	-1.03309432	2.24136182	-0.18225176
H	-0.30819277	1.97287245	1.42030579
O	3.06877919	2.13622285	-0.91281957
C	2.67363392	3.52138243	-1.04842845
C	1.39678417	3.63912545	-0.20961111
H	2.48620990	3.70810215	-2.10597910
H	3.48847535	4.14315453	-0.69012454
C	0.33427814	4.48729118	-0.88916056
C	1.69037762	4.12077225	1.20859177
H	0.04901107	4.04952560	-1.84577189
H	0.72511819	5.49086971	-1.06158087
H	-0.55098744	4.57160687	-0.26059370
H	0.80041634	4.04094347	1.83069038

H	-4.41306459	0.19655404	-2.03945085
H	-4.50075277	1.88854174	-1.46288789
C	-4.96556373	1.52358940	1.08147855
C	-5.90182120	-0.53981024	-0.03741370
H	-5.17981941	1.15422958	2.08308287
H	-5.82659347	2.09884441	0.73881364
H	-4.09601094	2.17960903	1.13318757
H	-5.65316504	-1.40596993	-0.65110305
H	-6.71367144	0.00795235	-0.51716579
H	-6.25744053	-0.88427980	0.93313130
C	3.64136132	-0.91056365	1.52799202
C	4.55146408	-0.79996265	-0.68164974
C	4.87791627	-1.31321303	2.02054138
H	2.80234362	-0.80706254	2.20838482
C	5.95786779	-1.45878086	1.15824676
H	4.99730299	-1.51552195	3.07687449
C	5.79165200	-1.19888145	-0.19710426
H	6.92064409	-1.77398905	1.53796013
H	6.62738163	-1.30968399	-0.87587306
H	4.43615159	-0.59790663	-1.74048199
C	-0.96572497	-1.20843755	-2.65833224
H	-0.06936592	0.67496415	-2.21277732
C	1.65058562	-2.31405832	-1.77953222
H	1.99131706	-0.41209428	-2.67977191
C	1.26482871	-2.99740005	-0.45812586
H	2.70988411	-2.52834333	-1.93517245

H	2.00752555	5.16428049	1.18602502
H	2.48164450	3.51827590	1.65567893
C	-3.56837721	-1.26896040	1.69251877
N	-3.49116225	-0.37133221	0.55086136
H	-4.14564359	-2.15455554	1.42508263
H	-4.05237183	-0.75737326	2.52331651
H	-2.56503430	-1.52681364	2.01142086
O	-2.74718997	0.88945661	-1.05409573
C	-4.17521456	0.88068845	-1.22254747
C	-4.69441253	0.37164743	0.11526663

H	1.12618370	-2.76557341	-2.62305012
H	1.22594800	-4.08078854	-0.62044860
H	2.06486272	-2.82790103	0.26084745
H	-0.07585371	-2.81737920	1.27636246
C	-1.40046806	-2.51005659	-1.94593738
H	-2.13364277	-2.60887047	0.14505600
H	-2.44821899	-2.71619073	-2.17059299
H	-0.83636345	-3.36652732	-2.31694870
H	-0.51919714	-1.44418257	-3.63032217
H	-1.85711096	-0.61437390	-2.86186551

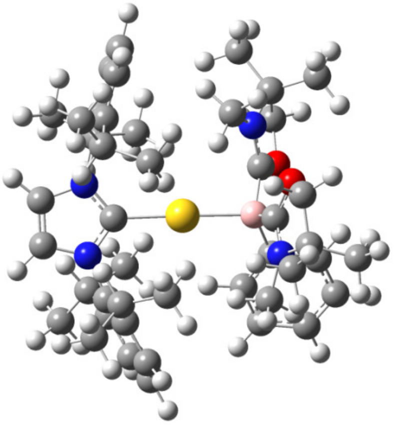

6

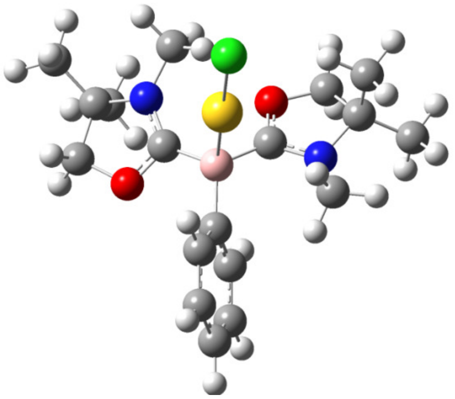

8

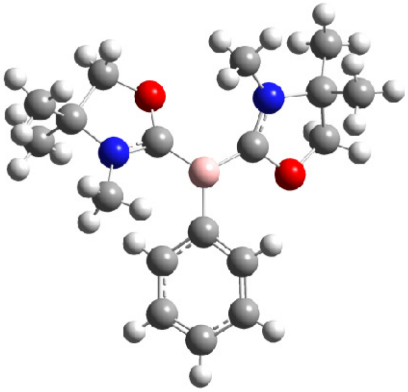

1

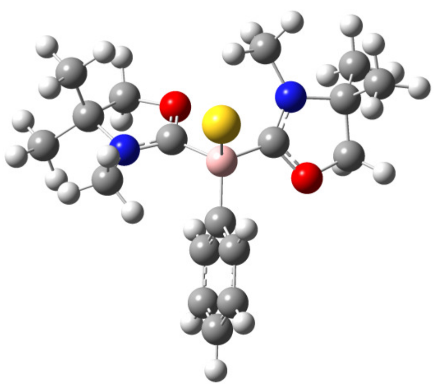

[1·Au]⁺

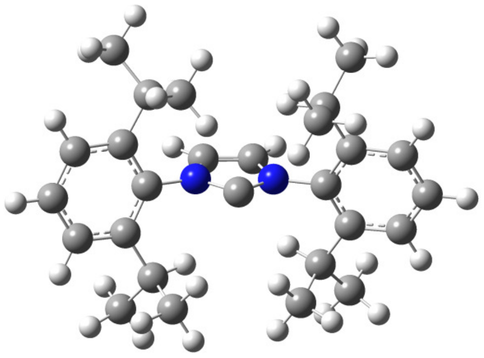

IPr

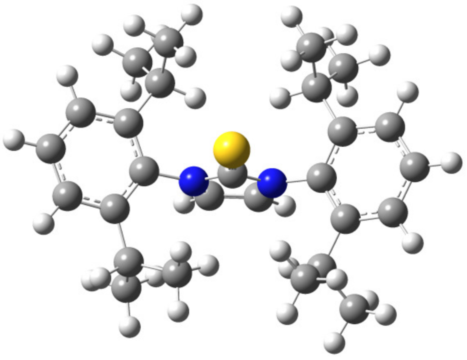

[IPr·Au]$^+$

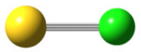

AuCl

6

Au	0.00648907	0.09183318	-0.08808482
B	-1.86403834	-0.96183810	-0.44585744
C	1.78173956	1.13871704	0.14300360
C	3.94550379	1.70430857	0.42935068
C	3.25166212	2.84735654	0.22015863
C	0.81289388	3.34258903	-0.20568564
C	0.03074314	3.73335437	0.88409466
C	0.40672509	3.40224234	2.31664309
C	0.65408484	4.68551229	3.11726125
C	-0.66001411	2.53630956	2.99265003
C	-1.11778904	4.47287157	0.61033216
C	-1.45495318	4.80571645	-0.69321576
C	-0.63860095	4.42775472	-1.74966323
C	0.52029140	3.68762774	-1.52862607
C	1.39630307	3.25174105	-2.68887908
C	1.67290950	4.40343592	-3.65940670
C	0.76648570	2.06398732	-3.42577168
C	3.34347777	-0.72411748	0.50713893
C	3.43058737	-1.27871913	1.78883365
C	3.11976206	-0.46629997	3.03160157
C	1.65570872	-0.68669270	3.43765853
C	4.05668056	-0.78504515	4.19859375
C	3.76315272	-2.62803634	1.88051206
C	3.99993687	-3.38194203	0.73873763
C	3.89428173	-2.80492096	-0.51732962

C	-1.02995069	-3.83526881	-2.70303950
C	-0.56315928	-4.49138234	-1.40885898
C	-1.58451250	-5.49875700	-0.88483508
C	0.82175612	-5.10438891	-1.53512797
C	0.14059094	-3.26442812	0.72342364
N	1.93221763	2.47422805	0.04202247
N	3.02328121	0.67178686	0.38399187
N	-3.09591279	-0.18376940	1.70202141
N	-0.57386724	-3.28840362	-0.53939333
O	-2.35830018	-2.23901612	1.69730355
O	-1.76879303	-2.67821904	-2.25193944
H	4.99068873	1.52773585	0.60636080
H	3.56790708	3.87354405	0.17694874
H	1.33466820	2.83069611	2.30736325
H	1.41954161	5.30129398	2.64571696
H	0.98059084	4.44114918	4.12839906
H	-0.25678456	5.28031662	3.19219992
H	-1.61295439	3.06575581	3.04219495
H	-0.35397335	2.30036981	4.01324085
H	-0.80732724	1.60342883	2.44574069
H	-1.75181362	4.79530762	1.42567237
H	-2.35443813	5.37337633	-0.88771191
H	-0.90868607	4.70817017	-2.75842241
H	2.35517814	2.92400607	-2.28714333
H	2.40466836	4.08926553	-4.40358456

C	3.55796344	-1.45923598	-0.66181340
C	3.43997876	-0.85083240	-2.04563035
C	4.78282724	-0.89094079	-2.78192297
C	2.34675696	-1.55753449	-2.85465016
C	-2.89591189	-0.20552020	-1.43192946
C	-2.62665537	1.01271207	-2.06121604
C	-3.57846873	1.65892300	-2.84341296
C	-4.83607889	1.09672520	-3.01970461
C	-5.12979104	-0.11857254	-2.41171540
C	-4.16920187	-0.75346930	-1.63341078
C	-2.44241002	-1.09664701	0.99973905
C	-2.80162819	-1.99374393	3.05127815
C	-3.69398431	-0.76192991	2.92833308
C	-5.15056015	-1.12895960	2.65632908
C	-3.55534598	0.15080245	4.13711862
C	-3.43359438	1.14733293	1.21822294
C	-1.37336596	-2.35090926	-1.02415740
H	4.68849277	-0.41592033	-3.75871215
H	5.11305920	-1.91840562	-2.93877612
H	2.62915560	-2.59111201	-3.06374470
H	2.20006645	-1.05266856	-3.80975919
H	1.39982451	-1.55119337	-2.31055316
H	-1.66138564	1.48154371	-1.91723646
H	-3.33793348	2.60482031	-3.31186185
H	-5.57928354	1.59667426	-3.62554558
H	-6.10424023	-0.56946717	-2.54631052
H	-4.41481510	-1.70427145	-1.17100437
H	-1.91918662	-1.79354425	3.66180499
H	-3.32244124	-2.87862646	3.40336001
H	-5.72319715	-0.23925073	2.39442703
H	-5.59815543	-1.57485341	3.54480483
H	-5.21618318	-1.83853607	1.83081543
H	-2.53505105	0.52352598	4.22455841

H	2.06186898	5.27819456	-3.13900763
H	0.76982800	4.69726434	-4.19434862
H	0.61610210	1.21577630	-2.75600771
H	1.40996419	1.75014544	-4.24926584
H	-0.20399484	2.34194122	-3.84056615
H	3.24325290	0.58892605	2.78793517
H	1.48897389	-1.74105063	3.67002928
H	0.97904974	-0.39646046	2.63187261
H	1.41711735	-0.09804196	4.32493743
H	3.88678495	-0.07580418	5.00843279
H	5.10214074	-0.72064687	3.89840432
H	3.87522499	-1.78284281	4.59852258
H	3.84866131	-3.09299453	2.85236350
H	4.27595498	-4.42371417	0.83142685
H	4.08952464	-3.40092836	-1.39916612
H	3.14507430	0.19251169	-1.94224268
H	5.55669868	-0.37025740	-2.21809569
H	-3.80261943	-0.40970453	5.03921830
H	-4.24199695	0.99408007	4.08016600
H	-2.57679466	1.55894901	0.68891458
H	-3.65715074	1.77384898	2.07759982
H	-4.28435508	1.11656162	0.53898506
H	-0.19526863	-3.48103402	-3.30851936
H	-1.69953255	-4.45276414	-3.29302459
H	-1.32661830	-5.81857035	0.12437335
H	-1.60109256	-6.37920141	-1.52778786
H	-2.58065714	-5.05562393	-0.86862659
H	1.55963163	-4.34130922	-1.77935954
H	0.81092554	-5.85211241	-2.32842267
H	1.11799233	-5.60229481	-0.61224703
H	0.10169186	-2.25817160	1.12721605
H	1.18185016	-3.52842067	0.54658280
H	-0.31091586	-3.95437901	1.43497187

8

Au	0.50930691	-0.83406308	-1.18345040
B	-0.25062795	0.67433689	0.14868267
C	0.68717037	0.19145564	1.28106509
C	2.61057722	-0.49761574	2.36580599
C	1.37019643	-0.96964177	3.11696982
C	2.76925170	1.21264204	0.49332490
C	3.63518075	0.11984332	3.30486870
C	3.21274773	-1.61663728	1.51700746

C	-1.83351877	-2.10564384	0.94974857
C	-4.19558079	-0.36188641	2.30384173
C	-4.71383021	-1.68489022	0.21801954
C	-0.05822984	2.19889183	-0.32493359
C	0.13402354	2.56948478	-1.65816108
C	0.30172647	3.90052923	-2.02659775
C	0.28081801	4.90070650	-1.06293067
C	0.08169144	4.55972585	0.27002548

C	-1.77890999	0.30880596	0.34742692
C	-3.87349508	-0.57289564	0.82612624
C	-3.98424584	0.72169270	0.02964434
N	-2.41754171	-0.80609422	0.67033570
O	0.27968376	-0.68633211	2.21814563
O	-2.65778141	1.27847934	0.09081321
H	1.37508695	-2.03587532	3.32685564
H	1.20707598	-0.39941683	4.03218664
H	2.76379713	0.65766407	-0.44915196
H	3.78443718	1.29999423	0.87095859
H	2.36424341	2.20421593	0.32187840
H	4.53495915	0.41625457	2.76745985
H	3.92960916	-0.61359045	4.05668505
H	3.21582812	0.99247183	3.80453169
H	2.46000640	-2.05130152	0.85666274
H	3.61607698	-2.39663356	2.16412646
H	4.02366827	-1.23706183	0.89593911
H	-4.21995111	0.53623462	-1.01873793

C	-0.08953045	3.22713802	0.62482241
Cl	1.36999703	-2.45307495	-2.67612425
N	1.97067098	0.51294803	1.49133272
H	-4.67495479	1.44192654	0.45732569
H	-0.82858143	-2.13202969	0.53885146
H	-2.43399846	-2.86872312	0.45956214
H	-1.79125575	-2.28880911	2.02166749
H	-3.94045291	-1.24989871	2.88180772
H	-5.26117382	-0.16617137	2.42885975
H	-3.63218516	0.48571960	2.69508504
H	-4.36741805	-1.92413757	-0.78715113
H	-5.75146972	-1.35443269	0.15971734
H	-4.68659803	-2.58500178	0.83068393
H	0.16734585	1.79774543	-2.41687579
H	0.45525065	4.15494727	-3.06708546
H	0.41784784	5.93550857	-1.34672969
H	0.05924768	5.33091991	1.02917335
H	-0.24187715	2.97566950	1.66920041

1

B	0.037912	0.338	0.023002
C	1.506144	0.104743	-0.17687
C	3.580167	0.655117	-0.90861
C	3.726809	-0.61084	-0.07517
C	4.281332	-0.27616	1.312157
C	4.55699	-1.67503	-0.77556
C	1.893842	-2.15351	0.747541
C	-0.9098	-0.81687	0.043059
C	-1.68543	-2.94125	-0.24783
C	-2.8977	-2.03153	-0.07839
C	-3.92364	-2.62048	0.876259
C	-3.52303	-1.67295	-1.42744
C	-2.86529	0.174051	1.218633
C	-0.50245	1.830286	-0.0063
C	-1.55232	2.202436	-0.85757
C	-2.04769	3.501209	-0.89657
C	-1.49069	4.482487	-0.08592
C	-0.43586	4.147515	0.756155
C	0.044358	2.844603	0.791295
N	2.303159	-0.98359	0.000503
N	-2.19655	-0.8734	0.483812
O	2.293434	1.154067	-0.55156
O	-0.61678	-2.03261	-0.52419

H	3.694196	0.525653	1.761152
H	5.318851	0.050732	1.229329
H	4.254606	-1.14182	1.973549
H	4.625818	-2.57852	-0.16889
H	5.570419	-1.30822	-0.94498
H	4.106304	-1.93131	-1.73364
H	1.13273	-1.863	1.475788
H	2.758553	-2.56092	1.268484
H	1.475327	-2.91598	0.095767
H	-1.47552	-3.48035	0.680844
H	-1.76842	-3.63361	-1.08178
H	-4.79943	-1.977	0.958596
H	-3.49061	-2.75833	1.867356
H	-4.25664	-3.58893	0.501214
H	3.59892300	-3.84278600	-0.19636500
H	-4.02891	-2.53729	-1.86118
H	-2.74754	-1.33379	-2.11513
H	-2.1125	0.75281	1.747471
H	-3.54732	-0.27355	1.941086
H	-3.41743	0.856368	0.568334
H	-1.98154	1.452707	-1.51344
H	-2.85915	3.750887	-1.56841
H	-1.86841	5.495757	-0.11429

H	4.323954	1.414368	-0.68169	H	0.010973	4.904482	1.38799
H	3.585699	0.424695	-1.97712	H	0.865341	2.600431	1.453966

[1·Au]+

B	0.00627142	0.37334399	-0.16835130	H	-3.29128343	0.55088833	-2.83855777
C	1.83020960	-2.45530511	-1.93215798	H	-4.14835352	1.42163788	-1.53292533
C	2.97218831	-1.96451637	-1.03986064	H	3.92808882	-2.45633245	0.84236936
N	2.35878596	-0.69697430	-0.55529041	H	2.27104341	-3.06644702	0.68889916
C	1.06178983	-0.69785644	-0.72270134	H	3.59892336	-3.84278652	-0.19636500
O	0.66216464	-1.73295814	-1.45126537	H	5.07177666	-1.43566812	-1.18519180
C	-3.40983823	0.66065634	-1.76152659	H	4.53274006	-2.64250478	-2.34105915
C	-3.65990908	-0.67325721	-1.05805147	H	4.09231948	-0.94409154	-2.58314660
N	-2.25678698	-0.98567260	-0.65826196	H	-4.59452775	-1.41554803	0.76283961
C	-1.49712429	0.08006835	-0.70288548	H	-4.08549602	0.28193965	0.83753670
O	-2.13315236	1.10441713	-1.23470312	H	-5.51715845	-0.18716185	-0.09560606
C	3.20436981	-2.88832871	0.15202751	H	-5.18612261	-1.39849097	-2.37697880
C	4.24572567	-1.72076751	-1.83444428	H	-3.55169569	-1.90387634	-2.83250073
C	-4.51134293	-0.48993020	0.19536880	H	-4.38543779	-2.67245715	-1.46549728
C	-4.22290046	-1.73225409	-1.99132410	H	-1.75591166	-3.00080233	-0.90667642
C	-1.91046646	-2.28361502	-0.10386735	H	-1.00732765	-2.19113118	0.49057517
C	3.08554116	0.21880824	0.31677224	H	-2.72298286	-2.61807261	0.53761012
Au	-0.05127056	-0.02631580	1.89149418	H	4.12897251	0.22341735	0.01546671
C	0.45290798	1.87218626	-0.52860240	H	2.67499895	1.21726518	0.21054834
C	0.38784214	2.94253939	0.36692933	H	2.99238450	-0.10613500	1.35390292
C	0.77574175	4.22387383	-0.00897076	H	0.03237606	2.77203597	1.37501675
C	1.23643375	4.46464027	-1.29641741	H	0.71815669	5.03394802	0.70513959
C	1.30036197	3.41808826	-2.20850562	H	1.54102482	5.45986808	-1.58867743
C	0.91206826	2.14151901	-1.82420723	H	1.65019035	3.59768266	-3.21596825
H	1.97034876	-2.18015319	-2.97606302	H	0.96306211	1.34084753	-2.55551240
H	1.63122472	-3.51808269	-1.83829878				

IPr

C	0.67327002	-0.03128683	1.91301524	H	2.70949907	3.17783841	-1.47318263
C	-0.67331618	0.03176812	1.91299713	H	1.15312645	-2.40366316	0.22778244
N	-1.05584553	0.04143953	0.57707676	H	1.05973929	-3.73106829	-1.86664985
C	-0.00000074	0.00019531	-0.28348385	H	1.18265648	-1.99301026	-2.20117387
N	1.05582412	-0.04095935	0.57708890	H	2.60530009	-3.03480931	-2.37611829
C	2.42552776	-0.11876962	0.16303420	H	2.04693680	-4.65913934	0.30841519
C	-2.42557899	0.11887501	0.16304798	H	3.06243870	-3.62541341	1.32441318
C	-2.94132722	1.35401325	-0.23494105	H	3.62064017	-4.13338948	-0.27336691
C	-4.28220082	1.40342058	-0.61460972	H	-1.77500144	-2.20923011	1.25912344
C	-5.07266374	0.26708036	-0.58284323	H	-3.00782339	-4.25632091	1.50923533
C	-4.53585365	-0.94898130	-0.18278474	H	-4.08503396	-2.94231704	2.00228956

C	-3.20017338	-1.04813769	0.19371955
C	3.20045915	1.04801844	0.19367465
C	4.53610952	0.94846979	-0.18282063
C	5.07255749	-0.26774852	-0.58291789
C	4.28174685	-1.40383827	-0.61472067
C	2.94089830	-1.35404574	-0.23500108
C	2.57213583	2.38557503	0.53566148
C	3.55313731	3.37928627	1.15753216
C	1.93553339	2.97769550	-0.72942370
C	2.07497759	-2.59498169	-0.32091199
C	1.70444857	-2.85438398	-1.78599270
C	2.74645713	-3.82235606	0.29990083
C	-2.57144048	-2.38548762	0.53575830
C	-3.55211581	-3.37956989	1.15756156
C	-1.93452236	-2.97742325	-0.72925257
C	-2.07573281	2.59518017	-0.32079983
C	-1.70478662	2.85446430	-1.78579999
C	-2.74769323	3.82248454	0.29961476
H	1.38568040	-0.06597786	2.71709996
H	-1.38573926	0.06647676	2.71707262
H	-4.70951246	2.34228628	-0.93848445
H	1.21436469	2.28470445	-1.16144057

H	-4.28545330	-3.72560900	0.42801515
H	-1.42748177	-3.91599935	-0.49649089
H	-1.21368285	-2.28413888	-1.16134311
H	-2.70837862	-3.17801492	-1.47300792
H	-1.15399419	2.40421246	0.22821336
H	-1.06045892	3.73144296	-1.86636386
H	-1.18248703	1.99325468	-2.20067499
H	-2.60552854	3.03437530	-2.37625938
H	-2.04831260	4.65937853	0.30837560
H	-3.06407473	3.62556293	1.32401032
H	-3.62168278	4.13336343	-0.27403431
H	-6.11224403	0.32570984	-0.87566779
H	-5.16331679	-1.82847663	-0.17072372
H	5.16385437	1.82776780	-0.17074844
H	6.11212074	-0.32666042	-0.87574270
H	4.70871192	-2.34282151	-0.93871068
H	1.77557122	2.20959581	1.25895386
H	3.00915930	4.25623806	1.50918933
H	4.08583624	2.94179793	2.00227618
H	4.28665684	3.72505903	0.42803880
H	1.42894662	3.91653670	-0.49676477

[IPr·Au]$^{+}$

Au	-0.00000782	-0.00006083	-1.58283940
N	1.07680325	0.01412385	1.17038062
C	0.00000045	-0.00003535	0.37464744
C	0.67811637	0.01035965	2.49160929
H	1.39125831	0.02010948	3.29544981
C	2.43705225	0.03063436	0.68899656
C	3.08441560	-1.19542757	0.51001395
C	4.38118604	-1.15318851	0.00460660
H	4.92041424	-2.07626676	-0.15626966
C	4.98885360	0.05682876	-0.29729176
H	5.99696050	0.06722693	-0.68754285
C	4.31618309	1.25340139	-0.09985427
H	4.80631652	2.18751918	-0.33703301
C	3.01663025	1.26920138	0.40091608
C	2.41858464	-2.52555849	0.80865272
H	1.47941705	-2.33642595	1.32920177
C	2.09305296	-3.26275492	-0.49576623
H	1.43610499	-2.66739950	-1.13273702
H	1.59840864	-4.21048591	-0.28169456

H	4.02840686	3.76317404	1.18071273
N	-1.07679633	-0.01413358	1.17038815
C	-0.67810081	-0.01037325	2.49161415
C	-2.43704771	-0.03059962	0.68900983
H	-1.39123747	-0.02010392	3.29545949
C	-3.08436487	1.19548525	0.51001675
C	-3.01666919	-1.26914666	0.40093218
C	-4.38113636	1.15329098	0.00460886
C	-2.41848764	2.52559330	0.80865582
C	-4.31622131	-1.25330196	-0.09983862
C	-2.29200448	-2.58636363	0.60237766
H	-4.92033093	2.07638796	-0.15627354
C	-4.98884826	-0.05670572	-0.29728232
H	-1.47932825	2.33642742	1.32920751
C	-2.09292375	3.26278010	-0.49576039
C	-3.28361316	3.39764672	1.72399066
H	-4.80638807	-2.18740365	-0.33701239
H	-1.31110741	-2.38474462	1.03467649
C	-2.07458452	-3.29710617	-0.73764694

H	3.00571385	-3.47397968	-1.05394493
C	3.28373769	-3.39758414	1.72398824
H	4.20226915	-3.70491188	1.22470678
H	2.73928820	-4.30124734	1.99716490
H	3.55432400	-2.86733530	2.63650595
C	2.29191867	2.58639315	0.60236020
H	1.31103145	2.38473952	1.03466516
C	2.07446653	3.29712357	-0.73766655
H	3.02720072	3.54152693	-1.20769375
H	1.52425262	4.22594184	-0.58666191
H	1.50854695	2.66969736	-1.42874767
C	3.05255765	3.48805464	1.58052413
H	3.20831686	2.99056073	2.53741879
H	2.49267823	4.40666770	1.75496865

C	-3.05267563	-3.48799057	1.58054851
H	-5.99695573	-0.06706935	-0.68753275
H	-1.43599489	2.66740098	-1.13272897
H	-1.59824415	4.21049165	-0.28168440
H	-3.00557294	3.47404180	-1.05394397
H	-4.20213876	3.70499636	1.22471185
H	-2.73913849	4.30129637	1.99716173
H	-3.55420981	2.86740841	2.63651135
H	-3.02732868	-3.54148155	-1.20766828
H	-1.52440073	-4.22594206	-0.58664076
H	-1.50864798	-2.66970151	-1.42873375
H	-3.20840345	-2.99048988	2.53744474
H	-2.49283843	-4.40663036	1.75498760
H	-4.02854181	-3.76306166	1.18074487

AuCl

Au	0.00000000	0.00000000	0.39767000
Cl	0.00000000	0.00000000	-1.84799400

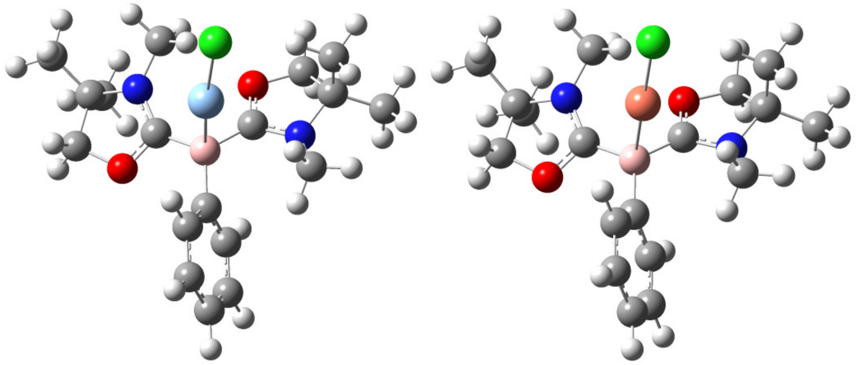

1·AgCl **1·CuCl (7)**

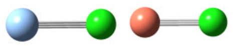

AgCl **CuCl**

1·AgCl

Ag	-0.50364897	-0.69818256	1.51424569
B	0.22890348	0.64685747	-0.25110057
C	-0.78676094	-0.06986047	-1.12158770
C	-2.77491956	-0.97276010	-1.89943876
C	-1.59015628	-1.53256717	-2.67416307

H	-1.51374379	-1.08808642	-3.66794012
H	-2.76832606	0.62106051	0.66526105
H	-3.88513015	1.04577440	-0.65752470
H	-2.45370411	2.03777111	-0.35240210
H	-4.77578679	-0.22020217	-2.26047641

C	-2.84251710	1.02544719	-0.35047291
C	-3.91182634	-0.56878440	-2.82488778
C	-3.23917062	-1.94306461	-0.81327046
C	1.70937052	0.20507954	-0.34444035
C	3.78391469	-0.78639876	-0.69641082
C	3.92821880	0.59786891	-0.07664273
C	1.72139341	-2.28372553	-0.54570037
C	4.07860908	-0.77169190	-2.19509514
C	4.62623918	-1.82072723	0.03356672
C	0.01816065	2.19463685	0.09166139
C	0.01132459	2.70794434	1.39227811
C	-0.16259649	4.06475327	1.64255798
C	-0.33375030	4.95180865	0.58734605
C	-0.31829538	4.47122143	-0.71669774
C	-0.13889675	3.11353512	-0.95377787
Cl	-1.39435299	-2.28142617	3.06692512
N	-2.10329231	0.19976208	-1.29265510
N	2.33052633	-0.96997304	-0.48160165
O	-0.44487745	-1.12095448	-1.90926874
O	2.62535701	1.18102300	-0.23410462
H	-1.59118810	-2.61755241	-2.73913418

H	-4.23079435	-1.43211577	-3.41052680
H	-3.58708938	0.22040088	-3.50219561
H	-2.40602048	-2.25673443	-0.18077319
H	-3.68527055	-2.82702707	-1.27044792
H	-3.98742397	-1.47887515	-0.17137364
H	4.15041084	0.54050749	0.99024119
H	4.64809478	1.23259284	-0.58465056
H	0.75083527	-2.25322644	-0.05739300
H	2.35540767	-2.98751990	-0.01150719
H	1.58074833	-2.60817717	-1.57473180
H	3.78739007	-1.71863876	-2.64938310
H	5.14512042	-0.62107813	-2.36676347
H	3.52508440	0.03359201	-2.67894237
H	4.30342967	-1.92049528	1.06978240
H	5.66861728	-1.50020565	0.02294806
H	4.57504297	-2.79374317	-0.45329757
H	0.13786380	2.03068349	2.22888956
H	-0.16952335	4.42783642	2.66179742
H	-0.47544426	6.00687659	0.77908346
H	-0.44303211	5.15420415	-1.54690992
H	-0.12910474	2.75155386	-1.97556405

1·CuCl (7)

Cu	-0.41220839	-0.45223516	1.54116173
B	0.13404506	0.57177410	-0.28394740
C	-0.87123522	-0.36678205	-0.90300153
C	-2.82848663	-1.54247078	-1.31860056
C	-1.65989885	-2.15571988	-2.07798904
C	-2.94908317	0.69130866	-0.14592792
C	-4.06190807	-1.40575046	-2.19746541
C	-3.12474207	-2.31850509	-0.03530284
C	1.63149341	0.20473114	-0.34792888
C	3.75065800	-0.72905282	-0.56147656
C	3.82940924	0.75915824	-0.24388520
C	1.77989566	-2.26834175	-0.04264097
C	4.01288357	-1.01134107	-2.03962834
C	4.66799989	-1.54250118	0.33853788
C	-0.18218123	2.12754591	-0.11899056
C	-0.15301268	2.78425088	1.11545565
C	-0.42573121	4.14320798	1.22626467
C	-0.73315955	4.88619840	0.09334304
C	-0.75692288	4.26120377	-1.14785987
C	-0.47943154	2.90278530	-1.24630396

H	-1.69792791	-1.90881716	-3.14050868
H	-2.76655863	0.46929837	0.91222247
H	-4.01083045	0.59202168	-0.35703651
H	-2.64320126	1.71229421	-0.34626086
H	-4.90243530	-0.99928774	-1.63616676
H	-4.36011880	-2.38760669	-2.56755683
H	-3.85232347	-0.75363330	-3.04473538
H	-2.22610036	-2.43032273	0.57465859
H	-3.51174356	-3.30849733	-0.28015192
H	-3.87107739	-1.80268788	0.56831771
H	4.08332843	0.93473778	0.80274512
H	4.49733404	1.31156651	-0.89809792
H	0.84554565	-2.19399321	0.50904453
H	2.48785329	-2.83116029	0.56039937
H	1.58861913	-2.78247307	-0.98194205
H	3.77270382	-2.04785568	-2.27712631
H	5.06437245	-0.83986045	-2.27378915
H	3.40041897	-0.35853899	-2.66229528
H	4.36554487	-1.45020860	1.38152045
H	5.68706509	-1.16771899	0.23695426

Cl	-0.95704549	-1.62876688	3.29588716
N	-2.22138201	-0.22875815	-1.00682032
N	2.31608811	-0.93936299	-0.26413791
O	-0.50019522	-1.52593095	-1.51043262
O	2.49100440	1.22999566	-0.46657445
H	-1.57314369	-3.23022997	-1.93788509

H	4.67419527	-2.59521164	0.05844146
H	0.07943355	2.21823887	2.01083100
H	-0.40270233	4.61988712	2.19721314
H	-0.95067066	5.94250858	0.17670887
H	-0.98919205	4.83217620	-2.03753874
H	-0.50349369	2.42513955	-2.21883370

AgCl

Ag	0.00000000	-0.00000000	0.62434310
Cl	-0.00000000	0.00000000	-1.72612504

CuCl

Cu	0.00000000	0.00000000	0.78896228
Cl	0.00000000	-0.00000000	-1.34587684

Supplementary Table 1 Raw Energy Data.

Compounds	E [au]	ZPE corr[a] [au]	H corr[b] [au]	G corr[c] [au]
**1**	-987.08561029	0.436311	0.460375	0.384212
**8**	-1137.46531924	0.439152	0.467303	0.37969
**6**	-2282.62554858	1.01839	1.075617	0.926304
**IPr**	-1160.19028039	0.576939	0.607816	0.514739
**[1·Au]$^+$**	-1122.32050237	0.438776	0.4653	0.380607
**[IPr·Au]$^+$**	-1295.39238294	0.579118	0.612167	0.512966
**AuCl**	-150.254844441	0.000815	0.004473	-0.024170

[a] Zero Point Energy Correction. [b] Enthalpy Correction. [c] Free Energy Correction.

Supplementary Table 2 Calculated bond dissociation energies D_e [kcal·mol^{-1}].

	D_e	D_e with ZPE corr[a]	D_e with H corr[b]	D_e with G corr[c]
B:→Au in **8**	78.3536617397	77.0823275555	76.8131259932	66.0243556928
B:→Au in **6**	92.5923793269	90.7343237891	90.6627877096	74.3155385325
C:→Au in **6**	72.0166387677	70.3380509380	70.4472375858	52.5902006262

[a] Zero Point Energy Correction. [b] Enthalpy Correction. [c] Free Energy Correction.

Supplementary Table 3 Raw Energy Data.

Compounds	E [au]	ZPE corr[a] [au]	H corr[b] [au]	G corr[c] [au]
**1**	-987.08561029	0.436311	0.460375	0.384212
**1·AgCl**	-1147.78136245	0.438603	0.466942	0.378608
**1·CuCl**	-1198.26913683	0.438408	0.466833	0.377844
**AgCl**	-160.606807730	0.000713	0.004422	-0.023600
**CuCl**	-211.081114545	0.000857	0.004495	-0.022532

[a] Zero Point Energy Correction. [b] Enthalpy Correction. [c] Free Energy Correction.

Supplementary Table 4 Calculated bond dissociation energies D_e [kcal·mol^{-1}].

	D_e	D_e with ZPE corr[a]	D_e with H corr[b]	D_e with G corr[c]
B:→Ag in **1·AgCl**	55.8134720399	54.8226345882	54.4674642288	44.5208116357
B:→Cu in **1·CuCl**	64.2644966017	63.4863848601	63.0326955141	54.1214335447

[a] Zero Point Energy Correction. [b] Enthalpy Correction. [c] Free Energy Correction.

Supplementary Table 5 The NPA charges of **6**.

Atom	No	Charge	Core	Natural Valence	Rydberg	Total
Au	1	0.12209	67.98685	10.87825	0.01281	78.87791
B	2	-0.22760	1.99851	3.19871	0.03037	5.22760
C	3	0.13597	1.99924	3.82018	0.04462	5.86403
C	4	-0.05706	1.99924	4.03878	0.01904	6.05706
C	5	-0.05421	1.99924	4.03589	0.01908	6.05421
C	6	0.12051	1.99882	3.85942	0.02125	5.87949
C	7	0.00024	1.99903	3.98115	0.01958	5.99976
C	8	-0.22599	1.99937	4.21202	0.01460	6.22599
C	9	-0.58828	1.99951	4.57943	0.00934	6.58828
C	10	-0.59743	1.99952	4.58850	0.00941	6.59743
C	11	-0.22286	1.99920	4.20869	0.01497	6.22286
C	12	-0.16933	1.99931	4.15399	0.01603	6.16933
C	13	-0.21816	1.99920	4.20387	0.01510	6.21816
C	14	0.00345	1.99903	3.97843	0.01908	5.99655
C	15	-0.23027	1.99937	4.21680	0.01410	6.23027
C	16	-0.59238	1.99951	4.58334	0.00953	6.59238
C	17	-0.59145	1.99952	4.58219	0.00975	6.59145
C	18	0.12532	1.99883	3.85463	0.02123	5.87468
C	19	0.00211	1.99905	3.97991	0.01894	5.99789
C	20	-0.22884	1.99938	4.21533	0.01414	6.22884
C	21	-0.59350	1.99951	4.58477	0.00921	6.59350
C	22	-0.59599	1.99952	4.58679	0.00969	6.59599
C	23	-0.22511	1.99920	4.21084	0.01507	6.22511
C	24	-0.18424	1.99931	4.16875	0.01618	6.18424
C	25	-0.22893	1.99920	4.21471	0.01502	6.22893
C	26	0.00647	1.99903	3.97486	0.01964	5.99353
C	27	-0.22770	1.99937	4.21394	0.01440	6.22770
C	28	-0.58872	1.99951	4.57982	0.00939	6.58872
C	29	-0.59433	1.99952	4.58493	0.00988	6.59433
C	30	-0.23838	1.99908	4.21329	0.02601	6.23838
C	31	-0.20885	1.99922	4.19373	0.01589	6.20885
C	32	-0.21322	1.99930	4.19856	0.01536	6.21322

C	33	-0.21423	1.99931	4.19926	0.01566	6.21423
C	34	-0.21207	1.99930	4.19674	0.01602	6.21207
C	35	-0.21354	1.99922	4.19816	0.01615	6.21354
C	36	0.53416	1.99886	3.42562	0.04136	5.46584
C	37	-0.04588	1.99921	4.02550	0.02117	6.04588
C	38	0.14826	1.99941	3.83380	0.01853	5.85174
C	39	-0.62101	1.99945	4.61011	0.01145	6.62101
C	40	-0.62171	1.99944	4.61037	0.01189	6.62171
C	41	-0.39053	1.99948	4.37679	0.01425	6.39053
C	42	0.55596	1.99895	3.40255	0.04255	5.44404
C	43	-0.04624	1.99920	4.02632	0.02072	6.04624
C	44	0.14717	1.99941	3.83532	0.01810	5.85283
C	45	-0.61920	1.99945	4.60820	0.01154	6.61920
C	46	-0.62545	1.99944	4.61430	0.01171	6.62545
C	47	-0.38651	1.99947	4.37225	0.01479	6.38651
N	48	-0.39964	1.99925	5.38566	0.01473	7.39964
N	49	-0.40266	1.99926	5.38884	0.01456	7.40266
N	50	-0.44584	1.99917	5.43134	0.01533	7.44584
N	51	-0.43839	1.99918	5.42429	0.01491	7.43839
O	52	-0.52829	1.99973	6.51832	0.01025	8.52829
O	53	-0.49304	1.99973	6.48305	0.01026	8.49304
H	54	0.24867	0.00000	0.75007	0.00126	0.75133
H	55	0.24962	0.00000	0.74910	0.00128	0.75038
H	56	0.21684	0.00000	0.78055	0.00260	0.78316
H	57	0.21270	0.00000	0.78602	0.00127	0.78730
H	58	0.21142	0.00000	0.78721	0.00137	0.78858
H	59	0.20522	0.00000	0.79338	0.00140	0.79478
H	60	0.20215	0.00000	0.79613	0.00172	0.79785
H	61	0.21054	0.00000	0.78767	0.00178	0.78946
H	62	0.21822	0.00000	0.77969	0.00209	0.78178
H	63	0.22194	0.00000	0.77572	0.00234	0.77806
H	64	0.22494	0.00000	0.77372	0.00134	0.77506
H	65	0.22607	0.00000	0.77167	0.00226	0.77393
H	66	0.21420	0.00000	0.78336	0.00245	0.78580
H	67	0.21456	0.00000	0.78410	0.00134	0.78544
H	68	0.21341	0.00000	0.78532	0.00127	0.78659
H	69	0.20370	0.00000	0.79490	0.00140	0.79630
H	70	0.21861	0.00000	0.77957	0.00182	0.78139
H	71	0.21039	0.00000	0.78792	0.00170	0.78961
H	72	0.21095	0.00000	0.78724	0.00181	0.78905
H	73	0.21945	0.00000	0.77817	0.00239	0.78055
H	74	0.20309	0.00000	0.79514	0.00177	0.79691
H	75	0.21955	0.00000	0.77918	0.00126	0.78045
H	76	0.21143	0.00000	0.78686	0.00171	0.78857

H	77	0.21622	0.00000	0.78248	0.00131	0.78378
H	78	0.21620	0.00000	0.78254	0.00125	0.78380
H	79	0.20205	0.00000	0.79654	0.00140	0.79795
H	80	0.22676	0.00000	0.77108	0.00216	0.77324
H	81	0.22486	0.00000	0.77382	0.00132	0.77514
H	82	0.22394	0.00000	0.77371	0.00235	0.77606
H	83	0.22083	0.00000	0.77628	0.00289	0.77917
H	84	0.21151	0.00000	0.78718	0.00130	0.78849
H	85	0.21420	0.00000	0.78445	0.00135	0.78580
H	86	0.20427	0.00000	0.79432	0.00141	0.79573
H	87	0.19722	0.00000	0.80100	0.00179	0.80278
H	88	0.21913	0.00000	0.77926	0.00160	0.78087
H	89	0.21782	0.00000	0.77984	0.00233	0.78218
H	90	0.21944	0.00000	0.77788	0.00268	0.78056
H	91	0.21156	0.00000	0.78660	0.00184	0.78844
H	92	0.21670	0.00000	0.78205	0.00125	0.78330
H	93	0.21520	0.00000	0.78346	0.00134	0.78480
H	94	0.20157	0.00000	0.79589	0.00254	0.79843
H	95	0.19583	0.00000	0.80223	0.00194	0.80417
H	96	0.21256	0.00000	0.78594	0.00150	0.78744
H	97	0.22407	0.00000	0.77469	0.00124	0.77593
H	98	0.22249	0.00000	0.77609	0.00143	0.77751
H	99	0.22080	0.00000	0.77785	0.00135	0.77920
H	100	0.22410	0.00000	0.77432	0.00157	0.77590
H	101	0.22853	0.00000	0.77021	0.00126	0.77147
H	102	0.22280	0.00000	0.77571	0.00148	0.77720
H	103	0.24497	0.00000	0.75295	0.00208	0.75503
H	104	0.20844	0.00000	0.78994	0.00162	0.79156
H	105	0.22967	0.00000	0.76851	0.00182	0.77033
H	106	0.19995	0.00000	0.79830	0.00175	0.80005
H	107	0.21454	0.00000	0.78400	0.00146	0.78546
H	108	0.21814	0.00000	0.78043	0.00143	0.78186
H	109	0.22311	0.00000	0.77545	0.00144	0.77689
H	110	0.22123	0.00000	0.77744	0.00133	0.77877
H	111	0.22464	0.00000	0.77368	0.00168	0.77536
H	112	0.22980	0.00000	0.76892	0.00127	0.77020
H	113	0.22236	0.00000	0.77629	0.00135	0.77764
H	114	0.24069	0.00000	0.75720	0.00211	0.75931
H	115	0.22024	0.00000	0.77784	0.00192	0.77976
H	116	0.20922	0.00000	0.78907	0.00171	0.79078

===

* Total *	1.00000	171.94943	287.04942	1.00116	460.0000

Supplementary Table 6 The NPA charges of **8**.

Atom	No	Charge	Core	Natural Valence	Rydberg	Total
Au	1	0.17304	67.98763	10.82751	0.01182	78.82696
B	2	-0.14606	1.99830	3.11071	0.03705	5.14606
C	3	0.49510	1.99890	3.46383	0.04216	5.50490
C	4	0.14680	1.99942	3.83449	0.01930	5.85320
C	5	-0.04434	1.99919	4.02356	0.02158	6.04434
C	6	-0.39198	1.99947	4.37870	0.01381	6.39198
C	7	-0.61726	1.99944	4.60559	0.01223	6.61726
C	8	-0.63033	1.99945	4.61951	0.01137	6.63033
C	9	0.56013	1.99902	3.39801	0.04284	5.43987
C	10	0.14606	1.99942	3.83662	0.01789	5.85394
C	11	-0.04761	1.99920	4.02749	0.02091	6.04761
C	12	-0.37989	1.99945	4.36577	0.01467	6.37989
C	13	-0.61975	1.99945	4.60884	0.01145	6.61975
C	14	-0.62427	1.99944	4.61293	0.01190	6.62427
C	15	-0.24234	1.99913	4.21660	0.02661	6.24234
C	16	-0.19599	1.99923	4.18081	0.01595	6.19599
C	17	-0.21168	1.99931	4.19670	0.01568	6.21168
C	18	-0.22310	1.99932	4.20824	0.01554	6.22310
C	19	-0.21855	1.99931	4.20330	0.01594	6.21855
C	20	-0.21793	1.99923	4.20238	0.01632	6.21793
Cl	21	-0.60731	10.00000	7.60000	0.00731	17.60731
N	22	-0.47130	1.99921	5.45629	0.01581	7.47130
N	23	-0.43813	1.99921	5.42399	0.01493	7.43813
O	24	-0.54051	1.99972	6.53009	0.01070	8.54051
O	25	-0.49661	1.99973	6.48656	0.01032	8.49661
H	26	0.20348	0.00000	0.79493	0.00159	0.79652
H	27	0.18952	0.00000	0.80847	0.00200	0.81048
H	28	0.22586	0.00000	0.77263	0.00151	0.77414
H	29	0.20216	0.00000	0.79641	0.00143	0.79784
H	30	0.24184	0.00000	0.75670	0.00146	0.75816
H	31	0.21844	0.00000	0.77999	0.00156	0.78156
H	32	0.21891	0.00000	0.77969	0.00140	0.78109
H	33	0.21946	0.00000	0.77847	0.00206	0.78054
H	34	0.24069	0.00000	0.75820	0.00110	0.75931
H	35	0.21454	0.00000	0.78393	0.00153	0.78546
H	36	0.22314	0.00000	0.77557	0.00129	0.77686
H	37	0.19831	0.00000	0.79980	0.00189	0.80169
H	38	0.20788	0.00000	0.79063	0.00149	0.79212
H	39	0.25079	0.00000	0.74784	0.00137	0.74921

H	40	0.20557	0.00000	0.79313	0.00130	0.79443
H	41	0.20476	0.00000	0.79371	0.00154	0.79524
H	42	0.21704	0.00000	0.78148	0.00148	0.78296
H	43	0.21677	0.00000	0.78171	0.00152	0.78323
H	44	0.22084	0.00000	0.77780	0.00136	0.77916
H	45	0.22802	0.00000	0.77036	0.00162	0.77198
H	46	0.22380	0.00000	0.77486	0.00134	0.77620
H	47	0.21976	0.00000	0.77879	0.00146	0.78024
H	48	0.22264	0.00000	0.77560	0.00176	0.77736
H	49	0.21137	0.00000	0.78730	0.00132	0.78863
H	50	0.20961	0.00000	0.78913	0.00126	0.79039
H	51	0.20787	0.00000	0.79073	0.00140	0.79213
H	52	0.20074	0.00000	0.79679	0.00247	0.79926
* Total *		0.00000	123.97120	141.53321	0.49559	266.00000

Supplementary Table 7 X-ray data for compounds **2**, **3**, **4**, **6** and **8**.

Compounds	**2**	**3**	**4·(Tol)**
Formula	$C_{19}H_{27}BF_3LiN_2O_5S$	$C_{26}H_{39}BClN_2O_2Rh$	$C_{33}H_{47}BClIrN_2O_2$
Fw	470.23	560.76	742.18
Crystsyst	triclinic	monoclinic	monoclinic
Space group	P -1	$P2_1/c$	P 1 2/c 1
Size (mm^3)	0.040 x 0.080 x 0.120	0.16 × 0.12 × 0.02	0.120 x 0.160 x 0.200
T/K	103(2)	103(2)	103(2)
a, Å	5.9513(6)	16.460(2)	35.4699(14)
b, Å	10.9562(11)	13.8072(16)	10.6670(5)
c, Å	17.3953(15)	11.9483(18)	18.1337(8)
α, deg	96.176(5)	90	90
β, deg	93.242(5)	104.628(9)	115.404(2)
γ, deg	97.646(6)	90	90
V, A^3	1114.73(19)	2627.4(6)	6197.6(5)
Z	2	4	8
d_{calcd} g·cm^{-3}	1.401	1.418	1.591
μ, mm^{-1}	0.203	0.777	4.427
Refl collected	15655	5132	74988

T_{max}/ T_{min}	0.9920/0.9760	0.985/0.886	0.6190/0.4710
N_{measd}	4579	4810	9999
[R int]	0.1403	0.1241	0.0876
R [I>2sigma(I)]	0.0685	0.0886	0.0285
R_w [I>2sigma(I)]	0.1408	0.1934	0.0583
GOF	0.996	1.005	1.017
Largest diff. peak/ hole[e. $Å^{-3}$]	0.347/-0.459	3.30/-0.91	1.136/-0.736

Compounds	**6·(THF)$_2$**	**8**
Formula	$C_{53}H_{79}AuBClN_4O_4$	$C_{18}H_{27}AuBClN_2O_2$
Fw	1079.42	546.64
Crystsyst	monoclinic	monoclinic
Space group	P 1 21/c 1	P 1 21/n 1
Size (mm^3)	0.020 x 0.120 x 0.360	0.100 x 0.160 x 0.180
T/K	103(2)	103(2)
a, Å	15.396(2)	11.2950(11)
b, Å	14.4925(19)	10.5120(8)
c, Å	24.966(4)	16.9583(16)
α, deg	90	90
β, deg	100.935(6)	102.328(4)
γ, deg	90	90
V, A^3	5469.4(13)	1967.1(3)
Z	4	4
d_{calcd} $g\cdot cm^{-3}$	1.311	1.846
μ, mm^{-1}	2.783	7.628
Refl collected	107465	3863
T_{max}/ T_{min}	0.9460/0.4340	0.5160/0.3410

N_{measd}	9566	3863
[R int]	0.1218	0.0680
R [I>2sigma(I)]	0.0756	0.0378
R_w [I>2sigma(I)]	0.1768	0.1297
GOF	1.126	1.151
Largest diff. peak/ hole[e. Å^{-3}]	3.610/-2.353	2.094/-3.545

Supplementary References

1. M. N. Cheemala and P. Knochel, New P, *Org. Lett*., 2007, **9**, 3089.
2. J. S. M. Samec and Jan-E. Bäckvall, *Chem.–Eur. J*., 2002, **8**, 2955.
3. A. Lühl, L. Hartenstein, S. Blechert and P. W. Roesky, *Organometallics*, 2012, **31**, 7109.
4. E. Kumaran and W. K. Leong, *Organometallics,* 2012, **31**, 1068.
5. N. Senthilkumar, P. Gandeepan, J. Jayakumar and C.-H. Cheng, *Chem. Commun.,* 2014, **50**, 3106.
6. V. Lavallo, J. H. Wright II, F. S. Tham and S. Quinlivan, *Angew. Chem., Int. Ed.,* 2013, **52**, 3172.
7. P. Zhao, C. Krug and J. F. Hartwig, *J. Am. Chem. Soc*., 2005, **127**, 12066**.**
8. J. M. Carney, P. J. Donoghue, W. M. Wuest, O. Wiest and P. Helquist, *Org. Lett*., 2008, **10**, 3903.
9. A. V. Butin, M. G. Uchuskin, A. S. Pilipenko, O. V. Serdyuk and I. V. Trushkov, *Tetrahedron Lett.,* 2011, **52**, 5255.
10. T. Kinoshita and Koji. Ichinose, *Heterocycles*, 2005, **65**, 1641.
11. M. Kumar, M. Scobie, M. S. Mashuta, G. B. Hammond and B. Xu, *Org. Lett*., 2013, **15**, 724.
12. C. Efe, I. N.vLykakis and M. Stratakis, *Chem. Commu.,* 2011, **47**, 803.
13. J. J. Kennedy-Smith, S. T. Staben, and F. D. Toste, *J. Am. Chem. Soc*., 2004, **126**, 4526.
14. Bruker AXS SHELXTL, Madison, WI; *SHELX-97G*. M. Sheldrick, ActaCrystallogr. A, 2008, **64**, 112; *SHELX-2013*, http://shelx.uni-ac. gwdg.de/SHELX/index.php.
15. Gaussian 09, Revision B.01, M. J. Frisch, G. W. Trucks, H. B. Schlegel, G. E. Scuseria, M. A. Robb, J. R. Cheeseman, G. Scalmani, V. Barone, B. Mennucci, G. A. Petersson, H. Nakatsuji, M. Caricato, X. Li, H. P. Hratchian, A. F. Izmaylov, J. Bloino, G. Zheng, J. L. Sonnenberg, M. Hada, M. Ehara, K. Toyota, R. Fukuda, J. Hasegawa, M. Ishida, T. Nakajima, Y. Honda, O. Kitao, H. Nakai, T. Vreven, J. A. Montgomery, Jr., J. E. Peralta, F. Ogliaro, M. Bearpark, J. J. Heyd, E. Brothers, K. N. Kudin, V. N. Staroverov, T. Keith, R. Kobayashi, J. Normand, K. Raghavachari, A. Rendell, J. C. Burant, S. S. Iyengar, J. Tomasi, M. Cossi, N. Rega, J. M. Millam, M. Klene, J. E. Knox, J. B. Cross, V. Bakken, C. Adamo, J. Jaramillo, R. Gomperts, R. E. Stratmann, O. Yazyev, A. J. Austin, R. Cammi, C. Pomelli, J. W. Ochterski, R. L. Martin, K. Morokuma, V. G. Zakrzewski, G.

A. Voth, P. Salvador, J. J. Dannenberg, S. Dapprich, A. D. Daniels, O. Farkas, J. B. Foresman, J. V. Ortiz, J. Cioslowski, D. J. Fox, Gaussian, Inc., Wallingford CT, 2010.

16. (a) G. Mazzone, N. Russo and E. Sicilia, *J. Chem. Theory Comput*., 2015, **11**, 581; (b) A. S. K. Hashmi, *Acc. Chem. Res.,* 2014, **47**, 864; (c) A. Gómez-Suárez and S. P. Nolan, *Angew. Chem., Int. Ed.*, 2012, **51**, 8156.
